# Interaction between Trinuclear Regium Complexes of Pyrazolate and Anions, a Computational Study

**DOI:** 10.3390/ijms21218036

**Published:** 2020-10-28

**Authors:** Ibon Alkorta, José Elguero, Cristina Trujillo, Goar Sánchez-Sanz

**Affiliations:** 1 Instituto de Química Médica, CSIC, Juan de la Cierva, 3, E-28006 Madrid, Spain; iqmbe17@iqm.csic.es; 2 Trinity Biomedical Sciences Institute, School of Chemistry, Trinity Dublin College, D02 R590 Dublin 2, Ireland; trujillc@tcd.ie; 3 Irish Centre of High-End Computing, Grand Canal Quay, Dublin 2, Ireland; 4 School of Chemistry, University College Dublin, Belfield, D02 HP83 Dublin 4, Ireland

**Keywords:** pyrazolate, regium bonds, non covalent interactions, electron density

## Abstract

The geometry, energy and electron density properties of the 1:1, 1:2 and 1:3 complexes between cyclic (Py-M)_3_ (M = Au, Ag and Cu) and halide ions (F^−^, Cl^−^ and Br^−^) were studied using Møller Plesset (MP2) computational methods. Three different configurations were explored. In two of them, the anions interact with the metal atoms in planar and apical dispositions, while in the last configuration, the anions interact with the CH(4) group of the pyrazole. The energetic results for the 1:2 and 1:3 complexes are a combination of the specific strength of the interaction plus a repulsive component due to the charge:charge coulombic term. However, stable minima structures with dissociation barriers for the anions indicate that those complexes are stable and (Py-M)_3_ can hold up to three anions simultaneously. A search in the CSD confirmed the presence of (Pyrazole-Cu)_3_ systems with two anions interacting in apical disposition.

## 1. Introduction

In biochemistry, supramolecular chemistry, molecular recognition and materials science, non-covalent interactions are of utmost importance; examples of their importance are their role in protein shapes [1], protein–protein interactions [2], anion recognition [3,4], drug recognition [5,6] and absorption on surfaces [7]. The oldest and most important non-covalent interaction is the hydrogen bond [8,9,10,11,12] but other interactions associated with atoms of columns 17–14 of the periodic table were described in the literature [13] such as halogen [14], chalcogen [15,16], pnictogen [17,18] and tetrel bonds [19,20], respectively. They were rationalized based on positive regions of the electrostatic potential surrounding the atoms acting as Lewis acids, with these regions being known as σ-holes [21].

More recently, the possibility to find σ-hole regions within atoms of column 11 of the periodic table (the coinage metals Cu, Ag and Au) in organometallic molecules or small nanoclusters was described [22,23,24,25]. However, only a limited number of complexes between derivatives of coinage atoms acting as Lewis acids and electron donors were explored in the literature [26]. This interaction was named the regium bond or metal-coinage bond [27]. However, it is worth noting that gold is an inert metal in bulk but in small nanostructures or in organometallic clusters it becomes a powerful catalyst [28,29].

Focusing on complexes with gold derivatives, the structures of the OC⋯AuX (with X = F, Cl and Br) [30], H_2_S⋯AuI [31] and H_2_⋯AuCl [32] complexes were determined by microwave spectroscopy. Additionally, the interaction of iodoperfluorobenzene derivatives with gold nanoparticles was investigated by Obenchain et al. using different spectroscopic techniques [33]. Besides, the NMR properties of tri(3,5-bis-trifluoromethyl-1*H*-pyrazole-silver) were determined in solution and in the solid state and compared with GIAO and ZORA calculated chemical shifts [34].

From the theoretical perspective, several computational studies were carried out in order to characterize this new type of interaction, mainly focusing on Au derivatives. Some examples can be: B⋯AuX complexes, with B = OC, H_2_O, H_2_S, C_2_H_2_ and C_2_H_4_ and X = F, Cl and Br, which were studied using the density functional theory at the BP86 level [35]. Additionally, the complexes between acetylene and AuX (X = OH, F, Cl, Br, CH_3_, CCH, CN and NC) presenting a π regium bond were also characterized at the MP2 computational level [36]. In addition, the strength and characteristics of regium bonded complexes were compared with other non-covalent interactions, for instance with halogen bond complexes by means of MP2 and coupled cluster (CCSD(T)) computational methods [37]. Furthermore, using atoms in molecules (AIM) and natural bond orbital (NBO) methods, the nature of the complexes between H_2_O and H_2_S acting as the Lewis basis and AuCl as a Lewis acid was characterized [38], and the intermolecular interaction between phosphines (XH_2_P with X = H, CH_3_, F, CN and NO_2_) and MY molecules (M = Cu, Ag, Au and Y = F, Cl, Br and I) [39]. Cooperativity in ternary complexes involving the regium bond was also explored [40,41,42,43]. The properties of complexes of Au(I) and Au(III) were compared using the CCSD(T)/CBS computational level [44]. Additionally, the spectroscopy of Au(CN)_3_ anions was described [45].

Focusing on diatomic Au_2_ and Au_n_ clusters; the spectroscopic properties of the Au_2_ complexes were calculated at the density functional theory (DFT) and coupled cluster levels [46,47]. Puru et al. studied the role of superatom model in gold clusters and nanoparticles [48]. Besides, the complexes of neutral and charged Au_2_ and Au with CO were examined at DFT (BP86, PW91 and B3LYP) and ab initio (MP2 and CCSD(T)) levels [49]. Non-conventional hydrogen bonds were established between small gold clusters (Au_3–7_) and formamide, formic acid, hydrogen fluoride and DNA bases [50,51,52]. In addition, the complexes between Au_n_ clusters (*n* = 2–6) with NH_3_ and NCH were characterized at the MP2 level [25] and the competition between halogen and regium bonds in binary complexes between CF_3_X (X = Cl, Br) and Au_n_ (*n*  =  2, 3 and 4) clusters was also explored [53]. In fact, the electrostatic properties of Au_n_ clusters with n = 2, 13, 55 and 147 were compared with the interaction energy of the mentioned complexes with CO and H_2_O [22]. It is also worth noting that different clusters of Au and AuCl can form regium-π bonds with aromatic systems [23,24]. In a recent paper, we studied the problem of regium vs. hydrogen bonds in M_2_⋯HX complexes, with M = Au, Ag and Cu, in which we found that regium bonds are not only competitive but in most of the cases stronger that hydrogen bonds [54,55].

**Figure 1:**
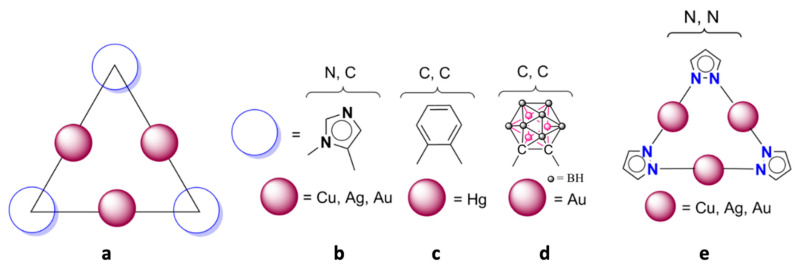
Structure of the studied compounds. (**a**) General structure, full red circles the metal; empty blue circles the ligand. (**b**) Imidazolate ligands linked by N and C atoms. (**c**) Phenylene ligands linked by C atoms. (**d**) 1,2-Dicarba-closo-dodecaborane ligands linked by C atoms; the structure of 1,2-dicarba-closo-dodecaborane was simplified. (**e**) Pyrazolate ligands linked by N atoms.

Triangular structures corresponding to nine-membered rings (three metal atoms and six atoms of the ligands) are well-known and they were reported in the literature (Figure 1, left hand side), for instance, imidazolates with coinage metals [56], phenylenes (most examples correspond to *o*-tetrafluorophenylene) with mercury [57,58] and 1,2-dicarba-*closo*-dodecaboranes with gold(I) [59,60]. Pyrazolate ligands (Py) with coinage metals, forming regium bonds, are also common, in any oxidation state: Cu(I)/Cu(II), Ag(I) and Au(I)/Au(III) [56,61,62,63,64,65,66]. Particularly, cyclic [Py-M(I)]_3_ systems with M = Cu, Ag and Au were studied [67] and the experimental evidences of the (Py-Cu)_3_ systems with simultaneous interactions of the three copper atoms with one hydroxyl group were recently reviewed [68]. So, these aforementioned systems present very interesting features, which make them good candidates for exhibiting regium bonds.

On a different topic, interaction between charged systems, i.e., anion–anion and cation–cation are very interesting, and were found both within the gas phase [69,70,71,72,73,74,75,76,77,78,79] and crystal structures [80,81]. This rare type of interaction, which, in principle should be repulsive, should lead to unstable complexes, i.e., they will revert into the separated ions. However, in the last decade, it was demonstrated that complexes exhibiting positive interaction energies are indeed minima and therefore stable structures [82,83]. Some examples can be found in the literature in which two charged conducting spheres can attract each other when they are in a close range distance [84]. The F⋯F interaction in negatively charged dimers was studied at MP2 level and using symmetry-adapted perturbation theory [85,86].

**Figure 2:**
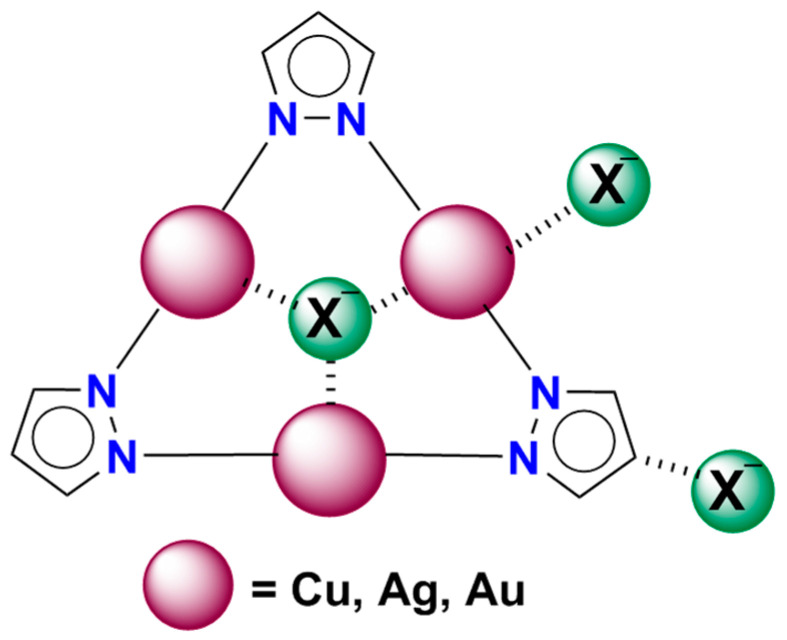
Schematic view of the possible complexes with X^−^ (X = F, Cl and Br). The scheme shows the possible interaction sites.

In the present work we will focus our efforts in the study of trinuclear regium pyrazolate systems interacting with one, two and three anions simultaneously to infer whether regium bonds or hydrogen bonds can be established and stabilize structures, which in principle should not be stable. We will investigate the interaction of (Pz-M)_3_, M = Cu, Ag and Au, with three anions (F^−^, Cl^−^ and Br^−^) limiting to the lowest oxidation states (I) of the three coinage metals (Figure 2).

## 2. Results and Discussion

### 2.1. Isolated (Pz-M)_3_ Monomers

The isolated (Pz-M)_3_ compounds (M = Cu, Ag and Au) were optimized at the MP2/aug’-cc-pVDZ computational level. All three of them showed a *D*_3*h*_ symmetry with each metal atom (M) located equidistantly between the two nitrogen atoms of the adjacent pyrazole rings. In fact, the calculated M-N distances are 1.817, 2.039 and 1.970, Å for the copper, silver and gold derivatives, respectively.

**Figure 3:**
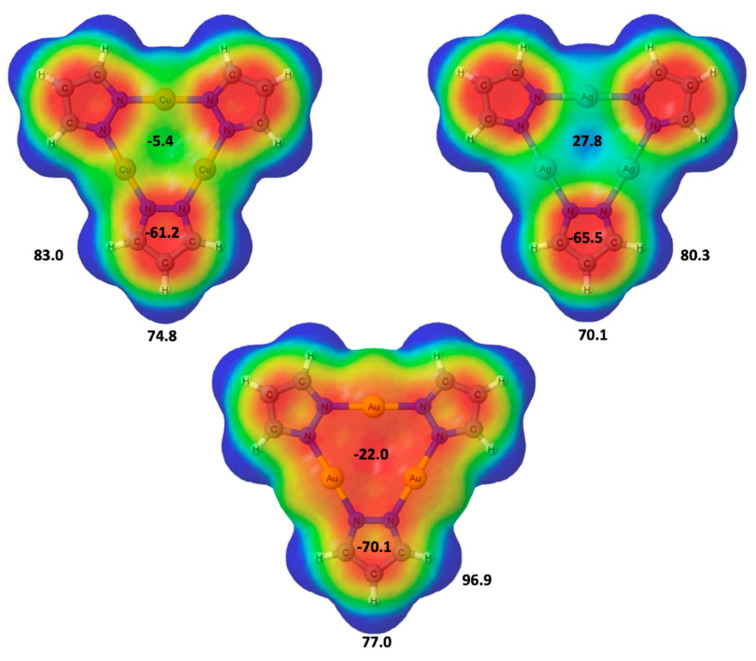
Molecular electrostatic potential of the (Pz-M)_3_ compound on the 0.001 au electron density isosurface. The color range associated to the MESP is between red ≤ −0.020 au and blue ≥ 0.015. Stationary values are given in kJ/mol.

To evaluate the areas of possible electrophilic attack, the molecular electrostatic potential (MESP) was calculated and plotted on the 0.001 au electron density isosurface in Figure 3. MESP showed negative (red) regions above and below of the pyrazole rings while the positive (blue) regions were associated to the hydrogen atoms in the periphery. The values were associated with four stationary points one minimum (over the pyrazole ring) and three maxima: two corresponding to the C-H bonds and one over the center of the system (*C*_3_ axis), for each of the three systems were also indicated in Figure 3 by their corresponding value. It is interesting to notice the dependence of the MESP sign along the *C*_3_ axis with the metal considered. While for the Au derivative the maximum shows a negative value (−22 kJ/mol), it becomes positive for silver (+28 kJ/mol) and very small and negative for copper (−5 kJ/mol) derivatives. Regarding the maxima associated to the CH groups of the pyrazole, in all cases the CH(4) exhibits is less positive MESP values than the CH(3) one, being the values of the former between 70 and 77 kJ/mol and those for the latter between 97 and 80 kJ/mol.

### 2.2. 1:1 Complexes

**Figure 4:**
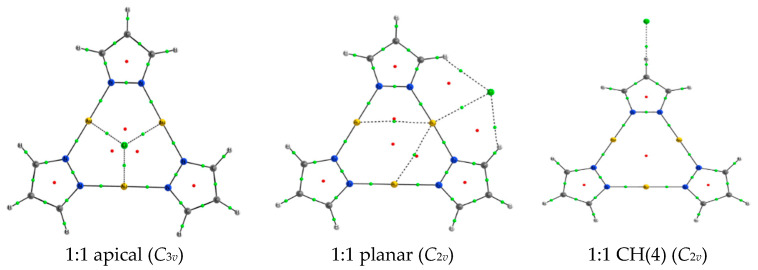
Molecular graph of the three minima located for the 1:1 (Pz-Au)_3_: Cl^−^. The symmetry of each complex is indicated. Small green and red dots indicate the position of the bond and ring critical points, respectively.

We began by studying the complexes established between a (Pz-M)_3_ unit and a single halide anion. All the molecular graphs have been included in Tables S1–S3. Three different energetic minima were found for each coinage metal derivative (Figure 4): (a) 1:1 apical, where the anion is located along the *C*_3_ symmetry axes and interacting simultaneously with the three metal atoms, (b) 1:1 planar, where the anion is within the molecular plane and simultaneously interacting with a metal atom and two CH(3) groups and (c) 1:1 CH(4), in which the anion interacts directly with a single hydrogen atom from the CH(4) group.

**Figure 5:**
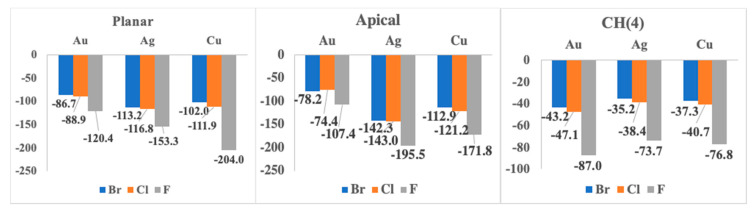
Binding energies in kJ/mol for each type of complex and anions with respect to the metal involved at the MP2/aug’-cc-pVDZ computational level.

The binding energies, E_b_, obtained as the difference of the energy of the complex minus the energy of the isolated monomers, for the 1:1 complexes were gathered in Table 1 and plotted in Figure 5. All the binding energies are shown to be large and negative as expected for the interaction between an anion with a neutral molecule, ranging between −204 and −35 kJ mol^−1^. The most stable complex of each configuration highly depends on the metal atom and on the anion considered. For instance, 1:1 apical complexes were found to be the most stable for silver and copper derivatives, while for gold derivatives 1:1 planar complexes are the most stable. The only exception is the copper complex with F^–^ where the planar complex is even more stable than the apical one. In all cases, the complexes showing only the interaction with the CH(4) group are the least stables ones.

Considering the same configuration, 1:1 apical and planar complexes, the most stable complex for a given anion corresponds to the silver derivative, followed by the copper and the gold derivative. The only exception corresponds to the 1:1 planar complexes with F^−^ where the copper complex is more stable than the silver one and the trend is Au < Ag < Cu. Similar trends, involving the metal atom, were already described in the literature for M⋯O interactions [87] and ethylene coinage metal complexes [88]. For all of the different 1:1 CH(4) complexes, in order of the most to least stable derivative we found gold, followed by copper and lastly silver.

Concerning the geometrical parameters, the M⋯X^−^ distances within the 1:1 apical and planar configurations were influenced by the size of the metal (Au >Ag > Cu) and the anion (F < Cl < Br) considered (Table 2). Focusing on configurations for the same metal and anion, in all the cases the M-X distance in the planar configuration was about 0.12 Å shorter than the apical one. This can be due to the fact that in the apical configuration, the anion was simultaneously interacting with three metals and thus the interaction was weakening (as observed for the binding energies) and therefore the M⋯X^−^ distance became longer. In the case of planar configuration, the anion was interacting with only one metal, plus there was a geometrical constraint due to the interactions with the CH(3) groups. Regarding the CH(4) configurations, the H⋯X^−^ distances found were shorter than those found for the CH(3)⋯X^−^ ones. The dependence on H(3)⋯X distances for a given anion change with the metal atom shows the following trend: Ag > Au > Cu, while for the H(4)⋯X^−^ it is Ag > Cu > Au.

No clear relationships between the binding energies and intermolecular distances were found for any of the complexes, except for the CH(4) complexes. This can highlight the complexity of the M⋯X^−^ interaction. The lack of correlation between those quantities can be associated with the electronic repulsion between the anion and the rest of the atoms in the (Pz-M)_3_ system. Additionally, the number of simultaneous interactions acting in 1:1 apical and planar configurations can play a role deviating the correlation between E_b_ and M⋯X^−^ distances.

Finally, the QTAIM analysis of the electron density (Figure 4 and Tables S1–S3) indicates the presence of three symmetrical bond paths in the apical configuration connecting the X^−^ anion and the three metal atoms. In the planar configuration, another three bond paths were found too, but in this case only one of them connects the anion with the metal atom while the other two corresponded to CH(3)⋯X^−^ interactions. 

Regarding, CH(4) configuration only one bond path was found between the anion and the CH(4) group. The electron density values of the intermolecular bond critical points (BCP; Table S4) present positive values of the Laplacian and negative values of the total energy density for the anion–metal bonds in the apical and planar configurations as an indication of the partial covalent nature of the interaction [89,90].

### 2.3. 1:2 Complexes

**Figure 6:**
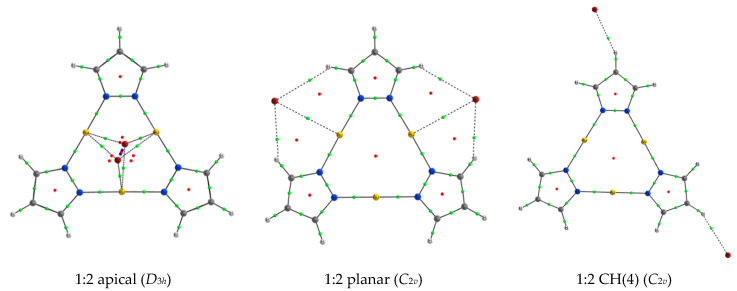
Molecular graph of the three minima located for the 1:2 (Pz-Au)_3_ and Br^−^. The symmetry of each complex is indicated. Small green, red and blue dots indicate the position of the bond, ring and cage critical points, respectively.

After analyzing the 1:1 complexes and their binding energies, the arising question was: can those negatively charged 1:1 complexes interact with another anion and produce stable structures? To address this question, three different 1:2 configurations were considered using the 1:1 complexes configurations as parental structures, i.e., 1:2 apical, 1:2 planar and 1:2 CH(4). There were a large number of possible combinations for the second anion to interact with the 1:1 complex, but for the sake of simplicity only two similar interactions will be explored simultaneously. In Figure 6 the structures corresponding to the 1:2 (Pz-M)_3_: Br_2_ complex were depicted as an illustrative example. In all the cases, the 1:2 apical complexes show *D*_3*h*_ symmetry while the planar and CH(4) ones present *C*_2*v*_ symmetry.

The binding energies for the 1:2 complexes (Table 3) varied from configuration to configuration ranging from +140 to −82 kJ mol^−1^. In the case of complexes with the largest halogen anions, Br^−^ and Cl^−^, the binding energies are found to be positive indicating a repulsive force within the complex. Only one exception was found for those anions, the 1:2 planar complex with silver were the binding energies were negative (−24 and −26 kJ mol^−1^, respectively). In contrast, the complexes with F^−^ present negative binding energies save for the 1:2 apical complexes with gold, which was positive (+113 kJ mol^−1^). As occurred for the 1:1 complexes, for each anion and configuration, the most stable complex corresponded to the silver one, except for the 1:2 planar and CH(4) configuration with F^−^ where the 1:2 planar with copper and gold were a more stable complex.

However, the existence of a stable minimum with positive values of the binding energy resembles to those minima in complexes between molecules with the same charge (anion–anion or cation–cation). When an energy scan corresponding to the separation of one of the anions from the rest of the system (X^−^⋯Pz-M-X^−^) in the apical complexes is done, it is observed a maximum in the potential energy surface that prevents the spontaneous dissociation of the anion (Figure S1 and Table S5). This fact has been previously described for anion–anion and cation–cation complexes in the literature [69,82,83]. The largest barriers were found for the F^−^ anion, reaching up to 96 kJ mol−1 for the 1:2 (Pz-Cu)_3_:F_2_. In contrast, the dissociation barriers for the 1:2 (Pz-Au)_3_:Cl_2_. and 1:2 (Pz-Au)_3_:Br_2_ were found to be small, 9 and 7 kJ mol−1, respectively. 

The energetic differences within the 1:2 complexes were clearly influenced by the electronic repulsion between the two X^−^ anions, which was essentially related to their X⋯X interatomic distance (Table S6). The shortest interatomic distances in the anions were found in the 1:2 apical complexes (between 2.7 and 5.5 Å), followed by the 1:2 planar complexes (between 7.4 and 12.2 Å) being the longest distances in the 1:2 CH(4) ones (between 13.2 and 16.0 Å), this means that the repulsive interaction between anions within the 1:2 apical configuration will be larger than those in the planar and CH(4) configuration. This is clearly observed in the binding energies for apical and planar configurations. However, when CH(4) complexes were taken into account, they did not follow the trend. This can be explained in terms of the type of interaction involved, since the binding energy depended both on the type/strength of the interaction involved plus the electronic repulsion between the anions. It is clear that while in the CH(4) the repulsion between the anions would be very small, the interaction C-H⋯X was also very weak.

One way to analyze the repulsion, or in other words, to narrow down the binding energy contribution of the anion–anion repulsion is to correct the binding energy by subtracting the charge–charge repulsion using the location of the anions with a charge of -1e [82]. As observed for the value in Table S7, corrected binding energies were in all the cases negative, which was aligned with the existence of those minima. However, in planar and CH(4) configurations, these values were smaller, in the absolute value, than twice of those for the 1:1 complexes, as expected for two identical charge transfer interactions over a neutral molecule. In contrast, in most of the 1:2 apical complexes the corrected energy was larger (in absolute value) than twice the 1:1 complexes binding energies, highlighting the importance of the charge transfer and polarization in these complexes.

Regarding the interatomic M⋯X and H⋯X distances (Table 4) in the 1:2 complexes, those were found to be slightly longer than the ones found for 1:1 complexes. This was consistent with the fact that the anions tend to separate from each other (due to Coulombic repulsion) while maintaining the same interaction patterns shown in the 1:1 complexes. The only exception corresponded to the planar 1:2(Pz-Au)_3_:F_2_ where each anion exclusively interacted with one of the H(3) atoms as shown in its molecular graph (Table S1).

Regarding the QTAIM analysis, the electron density properties (Table S8) show similar bond paths between the anions and the (Pz-M)_3_ systems in 1:2 complexes than those found for 1:1 complexes, with the aforementioned exception of the planar 1:2(Pz-Au)_3_:F_2_ complex. Another interesting feature of the QTAIM analysis in 1:2(Pz-Au)_3_:F_2_ complex was the presence of a bond path linking the two F^−^ anions.

As it is clear from the discussed result, the (Pz-Au)_3_ was capable of forming stable complexes with two anions simultaneously, but what is the limit of those complexes? Can 1:2(Pz-M)_3_:X_2_ form stable complexes with a third anion?

### 2.4. 1:3 Complexes

**Figure 7:**
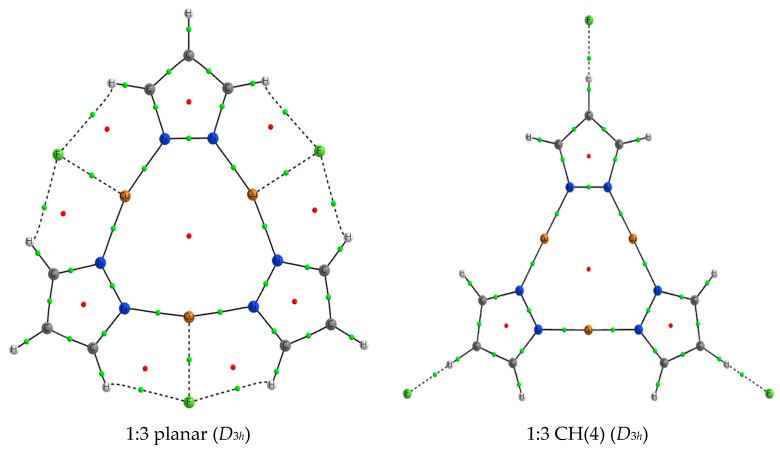
Molecular graph of the two minima located for the 1:3 (Pz-Au)_3_:F^−^. The symmetry of each complex is indicated. Small green and red dots indicate the position of the bond and ring critical points, respectively.

Following the same premises as that of 1:2 complexes, 1:3 complexes for the planar and CH(4) configuration were explored. Energetic minima structures were found for all the 1:3 CH(4) complexes and also for four of the nine 1:3 planar complexes. However, for the rest of the possible complexes, the anions dissociated spontaneously from the (Pz-M)_3_ molecule. In those stable cases, all the systems present *D*_3*h*_ symmetry. Two illustrative examples are shown in Figure 7.

The binding energies for the 1:3 complexes are gathered in Table 5. As occurred for 1:2 complexes, 1:3 complexes exhibit positive binding energies ranging between 160 and 260 kJ mol^−1^, much larger than those for the 1:2 complexes, although the structures found are still minima in the potential energy surface. Binding energies suggest that CH(4) configurations were more stable than the corresponding planar ones, while the other way around happened for the 1:2 complexes. In fact, in the latter, the shorter X^−^⋯X^−^ distances (Table S9) in the planar configuration (between 7.5 and 9.4 Å) penalize the binding energy versus those in CH(4) configuration where the anions were further away (between 13.2 and 15.6 Å). As for the 1:2 cases, removing the X^−^⋯X^−^ electrostatic repulsion term (Table S10) provided with a better picture of the binding energies. Corrected E_b_ values were negative but smaller than three times the binding energies found for the 1:1 complexes indicating a certain degree of anti-cooperativity in the 1:3 complexes.

Regarding the intermolecular M⋯X and H⋯X distances, selected geometrical parameters of the 1:3 minima are listed in Table 6. The comparison of these parameters with the corresponding in the 1:1 and 1:2 complexes ( Table 1; Table 3), indicates that within the (Pz-Ag)_3_X^−^ planar configuration there was a lengthening in the M⋯X^−^ distance from 1:3 vs. the 1:2 (∆R_M⋯X_ = 0.084, 0.291 and 0.389 Å for F^−^, Cl^−^ and Br^−^ respectively), which was larger than the difference between 1:2 and 1:1(Pz-Ag)_3_X^−^ complexes (0.071, 0.111 and 0.127 Å). This is an indication of a larger anti-cooperativity effect in the former than in the later complexes. Additionally, these effects were more acute for heavier halogens than for lighter ones. In case of CH(4) configuration, a similar pattern was found but the increment was milder across the halogen series. Curiously, the metal M also has influence on the H⋯X^−^ along the 1:1, 1:2 and 1:3 series for the CH(4) configuration, with a much larger increase for the (Pz-Cu)_3_X^−^ > (Pz-Ag)_3_X^−^ > (Pz-Au)_3_X^−^.

The molecular graph of the 1:3 planar complexes show three BCP between each anion and the (Pz-M)_3_ system, two of them with the CH(3) and one with the metal. In the case of the CH complexes a single bond path per anion was found. The BCPs (Table S11) exhibited similar electron density characteristics to those found for the 1:1 and 1:2 complexes.

A general analysis of all the BCPs extracted from this article shows excellent correlations between the electron density and the interatomic distance for each pair of atoms involved in the interactions (Figure S2). These results were in agreement with previous reports that have shown similar relationships [91,92,93].

### 2.5. CSD Search

**Figure 8:**
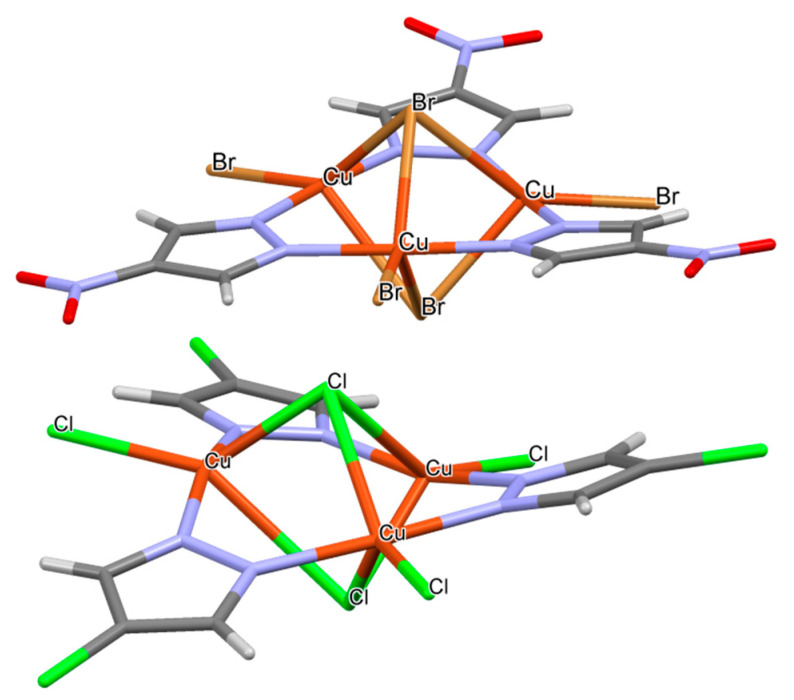
Structure of refcodes ELODOY (up) and OBOQAY (down). Only the (Pz-M)_3_: anions are shown for clarity.

A search in the CSD looking for (Pz-M)_3_ interacting with halides was carried out to investigate the number of crystals structures available. The search shows a total of thirteen crystal structures with the presence of halogen atoms in apical disposition interacting with copper (II) atoms linking the pyrazole rings. Of those structures, only one exhibited fluoride anions (CCDC refcode HUXWUU [94]), chloride is present in 10 crystal structures (JALKIT [95], OBOQAY [96], OBOQEC [96], OBOQIG [96], RETQIR [97], RUYGUN [98], RUYHAU [98], UWOMAW [99], VADYAB [100] and VAZCUX [101]) and bromide in two (ELODIS [102] and ELODOY [102]). It was observed that, in all these cases, two of the anions were simultaneously interacting with a single (Pz-M)_3_ molecule in the apical position as shown in Figure 8.

Ten of these structures (OBOQAY, OBOQEC, OBOQIG, RETQIR, RUYGUN, RUYHAU, UWOMAW, VAZCUX, ELODIS and ELODOY) present an additional halogen atom interacting with each copper atoms in planar configuration (see Figure 8 for two examples). The charge of the systems was compensated by the presence of bulky cations in the crystal (1-butyl-3-methyl-1H-imidazol-3-ium in VAZCUX and tetra-n-butylammonium in the rest).

The discrepancies between the interaction energies found for the 1:2 complexes in which planar complexes exhibited more negative interaction energies than apical ones could be due to the presence of counterions in the crystal structures that compensate the charge of the systems and the the repulsion of the anions that are absent in the gas phase calculations and due to crystal packing constraints.

The metal–halogen intermolecular distances were gathered in Table S12. As observed, those distances ranged between 2.38 and 2.61 (Cu-F), 2.34 and 3.06 (Cu-Cl) and 2.50 and 3.06 Å (Cu-Br), being the average distances 2.51, 2.61 and 2.72 Å for the Cu-F, Cu-Cl and Cu-Br interactions, respectively. In the case of Cu-F, the computational distance (2.11 Å) was shorter than the crystal one, while for the Cu-Cl and Cu-Br the computational distances were in fair agreement with the experimental ones.

## 3. Materials and Methods

The geometries of systems were fully optimized at the MP2 computational level [103] with a combination of the aug’-cc-pVDZ and the aug-cc-pVDZ-PP basis sets [104,105]. The aug’-cc-pVDZ basis set is built using aug-cc-pVDZ for C, N, F and Cl atoms and cc-pVDZ for the H atoms. For the heavy (coinage) atoms (Cu, Ag and Au) the effective core potential basis set, aug-cc-pVDZ-PP was used. Frequency calculations at the same computational level were carried out to confirm that the structures obtained correspond to energetic minima. These calculations were carried out with the Gaussian-16 program [106]. The electronic energy and geometry of all systems were gathered in Tables S1–S3.

The binding energy was calculated as the difference of the electronic energy of the complexes minus the sum of the energies of the isolated monomers in their minimum energy. Positive and negative values of the binding energies correspond to unfavorable (repulsive) and favorable (attractive) interactions. 

The topological characteristics of the electron density were studied within the quantum theory of atoms in molecules (QTAIM) [107,108] framework with the AIMAll program [109]. The molecular electrostatic potential (MESP) of the isolated monomers was represented with the Jmol program [110] and analyzed on the 0.001 au electron density isosurface with the Multiwfn program [111].

A search in the Cambridge Structural Database (CSD) [112] (Version 5.41 with updates of March, May and August 2020) was carried in order to find crystal structures of (Py-M)_3_ structures with halides. It should be noted that complementary studies focused on the analysis of the crystal structures that show the interaction between (Py-M)_3_ molecules and the hydroxyl anion are available in the literature [68].

## 4. Conclusions

The interactions between trinuclear regium complexes of pyrazolate with anions (F, Cl and Br) were studied by means of MP2 theory.

It was found that pyrazolate complexes could establish three type of modes of interactions, apical, planar and CH(4) involving different type of interactions and the strength of each interactions was highly dependent on the type of metal and anion considered. 

Considering the 1:1 complexes, (Pz-Ag)_3_F^−^ stood out as the strongest ones particularly within the planar configuration.

The most interesting question, which lay beneath the study, was: can those negatively charged (PzM)_3_X^−^ complexes interact and form stable complexes with another anion? Energetically speaking, stable 1:2 complexes were found but the binding energy was shown to be positive, which indicates a repulsive interaction. However, once the repulsion between anions was subtracted, the resulting corrected binding energies were negative. Potential energy surfaces corresponding to the removal of one of the anions indicated the existence of a barrier that prevented the anion dissociation.

Going one step forward, and trying to see what is the maximum number of anions that a pyrazolate complex can hold, the 1:3 complexes were also explored, finding that (Pz-Ag)_3_X (F, Cl and Br) were stable with large positive binding energies for both planar and CH(4) configurations.

A search in the CSD shows the presence of thirteen crystal structures of (Py-Cu)_3_ systems with two anions interacting in apical disposition.

This study involving anion⋯anion interactions will be very useful to analyze future interaction with transition metals and can bring more insight on these types of interactions, particularly in the crystal structure domain.

## Figures and Tables

**Table 1:** Binding energy, E_b_, (kJ mol^−1^) for the 1:1 complexes.

F^−^	1:1 Apical	1:1 Planar	1:1 CH (4)
Au	−107.4	−120.4	−87.0
Ag	−195.5	−153.3	−73.7
Cu	−171.8	−204.0	−76.8
Cl^−^			
Au	−74.4	−88.9	−47.1
Ag	−143.0	−116.8	−38.4
Cu	−121.2	−111.9	−40.7
Br^−^			
Au	−78.2	−86.7	−43.2
Ag	−142.3	−113.2	−35.2
Cu	−112.9	−102.0	−37.3

**Table 2:** Geometrical intermolecular M⋯X^−^ and H⋯X^−^ distances (Å) in the 1:1 complexes.

		F^−^			Cl^−^			Br^−^		
Complex	Dist.	Au	Ag	Cu	Au	Ag	Cu	Au	Ag	Cu
Apical	M⋯X	2.558	2.379	2.135	3.017	2.758	2.556	3.127	2.856	2.692
Planar	M⋯X	2.434	2.256	2.016	2.956	2.623	2.430	3.088	2.722	2.575
	H(3)⋯X	2.336	2.483	2.279	2.614	2.790	2.575	2.706	2.889	2.665
CH(4)	H(4)⋯X	1.508	1.554	1.551	2.317	2.366	2.357	2.507	2.556	2.547

**Table 3:** Binding energies (kJ mol^−1^) of the 1:2 complexes, (Pz-M)_3_: X_2_^−^ at the MP2/aug’-cc-pVDZ computational level.

	1:2 Apical	1:2 Planar	1:2 CH(4)
**F(-)**			
Au	113.1	−35.7	−39.6
Ag	−46.4	−79.0	−18.6
Cu	−34.7	−82.3	−20.3
**Cl(-)**			
Au	139.5	23.9	15.8
Ag	23.3	−25.8	29.2
Cu	93.5	2.1	28.1
**Br(-)**			
Au	120.4	20.2	18.8
Ag	15.3	−24.0	31.2
Cu	94.1	10.6	30.3

**Table 4:** Intermolecular M⋯X^−^ and H⋯X^−^ distances (Å) in the 1:2 complexes.

		F^−^			Cl^−^			Br^−^		
Complex	Dist.	Au	Ag	Cu	Au	Ag	Cu	Au	Ag	Cu
Apical	M⋯X	2.613	2.437	2.211	3.226	2.845	2.707	3.339	2.949	2.892
Planar	M⋯X	4.424 ^a^	2.327	2.090	3.325	2.734	2.639	3.455	2.849	2.962
	H(3)⋯·X ^b^	1.592	2.179	2.260	2.591	2.720	2.550	2.727	2.827	2.640
CH(4)	H(4)⋯·X	1.619	1.660	1.659	2.478	2.538	2.532	2.696	2.775	2.756

^a^ In this complex, the F^−^ ions interact only with one H(3) atoms. ^b^ The shorter of the two X⋯H(3) distances is listed.

**Table 5:** Binding energies (kJ mol^−1^) for the 1:3 planar and CH(4) complexes.

	F^−^		Cl^−^		Br^−^	
	1:3 Planar	1:3 CH(4)	1:3 Planar	1:3 CH(4)	1:3 Planar	1:3 CH(4)
Au	Dissociation	136.2	Dissociation	188.1	Dissociation	188.2
Ag	224.6	160.3	260.2	203.1	252.3	201.9
Cu	252.1	163.9	Dissociation	206.3	Dissociation	205.0

**Table 6:** Geometrical intermolecular distances (Å) in the 1:3 complexes.

		F^−^			Cl^−^			Br^−^		
Complex	Dist.	Au	Ag	Cu	Au	Ag	Cu	Au	Ag	Cu
Planar	M⋯X		2.411	2.211		3.025			3.238	
	H(3)⋯·X		2.461	2.387		2.863			2.942	
CH(4)	H(4)⋯·X	1.735	1.777	1.781	2.651	2.740	2.743	2.864	2.969	2.970

## Supplementary Materials

Supplementary Materials can be found at https://www.mdpi.com/1422-0067/21/21/8036/s1. Table S1. Molecular graph, electronic energy and geometry of the (Pz-Au)_3_:X^−^_n_ complexes; Table S2. Molecular graph, electronic energy and geometry of the (Pz-Ag)_3_:X^−^_n_ complexes; Table S3. Molecular graph, electronic energy and geometry of the (Pz-Cu)_3_:X^−^_n_ complexes; Table S4. Electron density properties at the intermolecular BCPs in the 1:1 complexes; Table S5. Barriers (kJ mol^−1^) and interhalogen distance (Å) in the maximum of the dissociation curve (Figure S1) of the 1:2 apical complexes; Table S6. Interatomic distances (Å) of the anions in the 1:2 complexes; Table S7. Anion-Anion repulsion corrected binding energies (kJ mol^−1^) in the 1:2 complexes; Table S8. Electron density properties at the intermolecular BCPs in the 1:2 complexes; Table S9. Interatomic distances (Å) of the anions in the 1:3 complexes; Table S10. Anion-Anion repulsion corrected binding energies (kJ/mol) in the 1:3 complexes; Table S11. Electron density properties at the intermolecular BCPs in the 1:3 complexes; Table S12. Anion-Metal distances in the CSD search; Figure S1. Energy profiles (kJ mol^−1^) as a function of the X-X distance (Å) in the 1:2 apical complexes. Only one of the X anions in move further away from the (Pz-M)_3_ system; Figure S2. Electron density at the BCP (au) vs. the interatomic distance (Å).

## References

[1] Frieden E. (1975). Non-covalent interactions: Key to biological flexibility and specificity. J. Chem. Educ..

[2] Zhu H., Sommer I., Lengauer T., Domingues F.S. (2008). Alignment of Non-Covalent Interactions at Protein-Protein Interfaces. PLoS ONE.

[3] Busschaert N., Caltagirone C., Van Rossom W., Gale P.A. (2015). Applications of Supramolecular Anion Recognition. Chem. Rev..

[4] Molina P., Zapata F., Caballero A. (2017). Anion Recognition Strategies Based on Combined Noncovalent Interactions. Chem. Rev..

[5] Cockroft S.L., Hunter C.A. (2007). Chemical double-mutant cycles: Dissecting non-covalent interactions. Chem. Soc. Rev..

[6] Maharramov A.M., Mahmudov K.T., Kopylovich M.N., Pombeiro A.J.L. (2016). Non-Covalent Interactions in the Synthesis and Design of New Compounds.

[7] Mahadevi A.S., Sastry G.N. (2013). Cation−π Interaction: Its Role and Relevance in Chemistry, Biology, and Material Science. Chem. Rev..

[8] Pimentel G., McClellan A. (1960). The Hydrogen Bond.

[9] Hadzi D. (1957). Hydrogen Bonding. Proceedings of the Symposium on Hydrogen Bonding.

[10] Speakman J.C. (1975). The Hydrogen Bond and other Inter-Molecular Forces.

[11] Jeffrey G.A., Saenger W. (1994). Hydrogen Bonding in Biological Structures.

[12] Desiraju G.R., Steiner T. (1999). The Weak Hydrogen Bond.

[13] Alkorta I., Elguero J., Frontera A. (2020). Not Only Hydrogen Bonds: Other Noncovalent Interactions. Crystals.

[14] Cavallo G., Metrangolo P., Milani R., Pilati T., Priimagi A., Resnati G., Terraneo G. (2016). The Halogen Bond. Chem. Rev..

[15] Wang W., Ji B., Zhang Y. (2009). Chalcogen Bond: A Sister Noncovalent Bond to Halogen Bond. J. Phys. Chem. A.

[16] Minyaev R.M., Minkin V.I. (1998). Theoretical study of O - > X (S, Se, Te) coordination in organic compounds. Can. J. Chem..

[17] Zahn S., Frank R., Hey-Hawkins E., Kirchner B. (2011). Pnicogen Bonds: A New Molecular Linker?. Chem. Eur. J..

[18] Del Bene J.E., Alkorta I., Elguero J., Scheiner S. (2015). The Pnicogen Bond in Review. Structures, Binding Energies, Bonding Properties, and Spin-Spin Coupling Constants of Complexes Stabilized by Pnicogen Bonds.

[19] Alkorta I., Rozas I., Elguero J. (2001). Molecular Complexes between Silicon Derivatives and Electron-Rich Groups. J. Phys. Chem. A.

[20] Bauzá A., Mooibroek T.J., Frontera A. (2013). Tetrel-Bonding Interaction: Rediscovered Supramolecular Force?. Angew. Chem. Int. Ed..

[21] Politzer P., Murray J.S., Clark T. (2013). Halogen bonding and other [sigma]-hole interactions: A perspective. Phys. Chem. Chem. Phys..

[22] Stenlid J.H., Brinck T. (2017). Extending the σ-Hole Concept to Metals: An Electrostatic Interpretation of the Effects of Nanostructure in Gold and Platinum Catalysis. J. Am. Chem. Soc..

[23] Bauzá A., Frontera A. (2018). Regium-π vs Cation-π Interactions in M_2_ and MCl (M = Cu, Ag and Au) Complexes with Small Aromatic Systems: An ab Initio Study. Inorganics.

[24] Frontera A., Bauzá A. (2018). Regium–π bonds: An Unexplored Link between Noble Metal Nanoparticles and Aromatic Surfaces. Chem. Eur. J..

[25] Zierkiewicz W., Michalczyk M., Scheiner S. (2018). Regium bonds between Mn clusters (M = Cu, Ag, Au and n = 2–6) and nucleophiles NH3 and HCN. Phys. Chem. Chem. Phys..

[26] Legon A.C., Walker N.R. (2018). What’s in a name? ‘Coinage-metal’ non-covalent bonds and their definition. Phys. Chem. Chem. Phys..

[27] Halldin Stenlid J., Johansson A.J., Brinck T. (2018). σ-Holes and σ-lumps direct the Lewis basic and acidic interactions of noble metal nanoparticles: Introducing regium bonds. Phys. Chem. Chem. Phys..

[28] Assadollahzadeh B., Schwerdtfeger P. (2009). A systematic search for minimum structures of small gold clusters Au_n_ (n = 2–20) and their electronic properties. J. Chem. Phys..

[29] Slaughter L.M. (2015). Homogeneous Gold Catalysis.

[30] Evans C.J., Reynard L.M., Gerry M.C.L. (2001). Pure Rotational Spectra, Structures, and Hyperfine Constants of OC−AuX (X = F, Cl, Br). Inorg. Chem..

[31] Medcraft C., Bittner D.M., Tew D.P., Walker N.R., Legon A.C. (2016). Geometries of H_2_S⋯MI (M = Cu, Ag, Au) complexes studied by rotational spectroscopy: The effect of the metal atom. J. Chem. Phys..

[32] Obenchain D.A., Frank D.S., Grubbs G.S., Pickett H.M., Novick S.E. (2017). The covalent interaction between dihydrogen and gold: A rotational spectroscopic study of H_2_–AuCl. J. Chem. Phys..

[33] Blakey I., Merican Z., Rintoul L., Chuang Y.-M., Jack K.S., Micallef A.S. (2012). Interactions of iodoperfluorobenzene compounds with gold nanoparticles. Phys. Chem. Chem. Phys..

[34] Alkorta I., Elguero J., Dias H.V.R., Parasar D., Martín-Pastor M. (2020). An experimental and computational NMR study of organometallic nine-membered rings: Trinuclear silver(I) complexes of pyrazolate ligands. Mag. Res. Chem..

[35] Zhang G., Yue H., Weinhold F., Wang H., Li H., Chen D. (2015). Resonance Character of Copper/Silver/Gold Bonding in Small Molecule⋅⋅⋅M-X (X=F, Cl, Br, CH_3_, CF_3_) Complexes. ChemPhysChem.

[36] Li H., Li Q., Li R., Li W., Cheng J. (2011). Prediction and characterization of HCCH⋅⋅⋅AuX (X = OH, F, Cl, Br, CH_3_, CCH, CN, and NC) complexes: A π Au-bond. J. Chem. Phys..

[37] Gao M., Li Q., Li H.-B., Li W., Cheng J. (2015). How do organic gold compounds and organic halogen molecules interact? Comparison with hydrogen bonds. RSC Adv..

[38] Zhang G., Zhao X., Chen D. (2013). Dual Bonding between H_2_O/H_2_S and AgCl/CuCl: Cu/Ag Bond, Sister Bond to Au Bond. J. Phys. Chem. A.

[39] Wang Z., Liu Y., Zheng B., Zhou F., Jiao Y., Liu Y., Ding X., Lu T. (2018). A theoretical investigation on Cu/Ag/Au bonding in XH_2_P⋯MY(X = H, CH_3_, F, CN, NO_2_; M = Cu, Ag, Au; Y = F, Cl, Br, I) complexes. J. Chem. Phys..

[40] Gao M., Yang X., Cheng J., Li Q., Li W., Loffredo R.E. (2013). Interplay between Metal⋯π Interactions and Hydrogen Bonds: Some Unusual Synergetic Effects of Coinage Metals and Substituents. ChemPhysChem.

[41] Gao M., Li Q., Li W., Cheng J. (2015). Interplay between Cation–π and Coinage-Metal–Oxygen Interactions: An Ab Initio Study and Cambridge Structural Database Survey. ChemPhysChem.

[42] Wei Y., Cheng J., Li W., Li Q. (2017). Regulation of coin metal substituents and cooperativity on the strength and nature of tetrel bonds. RSC Adv..

[43] Zheng B., Liu Y., Huang L., Wang Z., Liu H., Liu Y. (2018). Cooperative effects between F … Ag bonded and X … Br (Cl) halogen-bonded interaction in BrF(ClF) … AgX … BrF(ClF) (X = F, Cl, Br) complexes: A theoretical study. Mol. Phys..

[44] Trujillo C., Sánchez-Sanz G., Elguero J., Alkorta I. (2020). The Lewis acidities of gold(I) and gold(III) derivatives: A theoretical study of complexes of AuCl and AuCl_3_. Struct. Chem..

[45] Samanta D., Wu M.M., Jena P. (2011). Unique Spectroscopic Signature of Nearly Degenerate Isomers of Au(CN)_3_ Anion. J. Phys. Chem. Lett..

[46] Wang X., Wan X., Zhou H., Takami S., Kubo M., Miyamoto A. (2002). Electronic structures and spectroscopic properties of dimers Cu_2_, Ag_2_, and Au_2_ calculated by density functional theory. J. Mol. Struct. THEOCHEM.

[47] Wesendrup R., Laerdahl J.K., Schwerdtfeger P. (1999). Relativistic effects in gold chemistry. VI. Coupled cluster calculations for the isoelectronic series AuPt^−^, Au_2_, and AuHg^+^. J. Chem. Phys..

[48] Jena P. (2013). Beyond the Periodic Table of Elements: The Role of Superatoms. J. Phys. Chem. Lett..

[49] Schwerdtfeger P., Lein M., Krawczyk R.P., Jacob C.R. (2008). The adsorption of CO on charged and neutral Au and Au_2_: A comparison between wave-function based and density functional theory. J. Chem. Phys..

[50] Kryachko E.S., Remacle F. (2005). Complexes of DNA Bases and Gold Clusters Au3 and Au4 Involving Nonconventional N−H⋯Au Hydrogen Bonding. Nano Lett..

[51] Kryachko E.S., Remacle F. (2005). Three-gold clusters form nonconventional hydrogen bonds O–H⋯Au and N–H⋯Au with formamide and formic acid. Chem. Phys. Lett..

[52] Kryachko E.S., Karpfen A., Remacle F. (2005). Nonconventional Hydrogen Bonding between Clusters of Gold and Hydrogen Fluoride. J. Phys. Chem. A.

[53] Zhao Q. (2014). The X⋯Au interactions in the CF_3_X (X = Cl, Br) ⋯Au_n_ (n = 2, 3, and 4) complexes. J. Mol. Model..

[54] Sánchez-Sanz G., Trujillo C., Alkorta I., Elguero J. (2019). Understanding Regium Bonds and their Competition with Hydrogen Bonds in Au_2_:HX Complexes. ChemPhysChem.

[55] Sanchez-Sanz G., Trujillo C., Alkorta I., Elguero J. (2020). Rivalry between regium and hydrogen bonds established within diatomic coinage molecules and Lewis acids/bases. ChemPhysChem.

[56] Tekarli S.M., Cundari T.R., Omary M.A. (2008). Rational Design of Macrometallocyclic Trinuclear Complexes with Superior π-Acidity and π-Basicity. JACS.

[57] Awad S.B., Brown D.S., Cohen S.C., Humphries R.E., Massey A.G. (1977). A reinvestigation of phenylene- and polyphenylene-mercurials. J. Organomet. Chem..

[58] Tsunoda M., Gabbaï F.P. (2000). μ6-η2:η2:η2:η2:η2:η2 As a New Bonding Mode for Benzene. J. Am. Chem. Soc..

[59] Aullón G., Laguna A., Oliva J.M. (2012). Electronic structure and geometries of o-carborane derived cyclic structures [{μ-1,2-(C_2_B_10_H_10_)_n_Mn}Ag_m_]^z−^, M = {Au, Hg}, n = {3, 4}, m = {0, 1, 2}, z = {n − m, −m}. Dalton Trans..

[60] Aullón G., Laguna A., Filippov O.A., Oliva-Enrich J.M. (2019). Trinuclear Gold–Carborane Cluster as a Host Structure. Eur. J. Inorg. Chem..

[61] Yang G., Raptis R.G. (2003). Supramolecular Assembly of Trimeric Gold(I) Pyrazolates through Aurophilic Attractions. Inorg. Chem..

[62] Yang G., Martínez J.R., Raptis R.G. (2009). Dinuclear gold(III) pyrazolato complexes—Synthesis, structural characterization and transformation to their trinuclear gold(I) and gold(I/III) analogues. Inorg. Chim. Acta.

[63] Osuga T., Murase T., Hoshino M., Fujita M. (2014). A Tray-Shaped, PdII-Clipped Au_3_ Complex as a Scaffold for the Modular Assembly of [3×n] Au Ion Clusters. Angew. Chem. Int. Ed..

[64] Rasika Dias H.V., Palehepitiya Gamage C.S. (2007). Arene-Sandwiched Silver(I) Pyrazolates. Angew. Chem. Int. Ed..

[65] Saotome M., Shimizu D., Itagaki A., Young D.J., Fujisawa K. (2019). Structures and Photoluminescence of Silver(I) and Gold(I) Cyclic Trinuclear Complexes with Aryl Substituted Pyrazolates. Chem. Lett..

[66] Zheng J., Lu Z., Wu K., Ning G.-H., Li D. (2020). Coinage-Metal-Based Cyclic Trinuclear Complexes with Metal–Metal Interactions: Theories to Experiments and Structures to Functions. Chem. Rev..

[67] Caramori G.F., Piccoli R.M., Segala M., Muñoz-Castro A., Guajardo-Maturana R., Andrada D.M., Frenking G. (2015). Cyclic trinuclear copper(i), silver(i), and gold(i) complexes: A theoretical insight. Dalton Trans..

[68] Pandolfo L., Pettinari C. (2017). Trinuclear copper(ii) pyrazolate compounds: A long story of serendipitous discoveries and rational design. CrystEngComm.

[69] Mata I., Alkorta I., Molins E., Espinosa E. (2012). Electrostatics at the Origin of the Stability of Phosphate-Phosphate Complexes Locked by Hydrogen Bonds. ChemPhysChem.

[70] Mata I., Alkorta I., Molins E., Espinosa E. (2013). Tracing environment effects that influence the stability of anion–anion complexes: The case of phosphate–phosphate interactions. Chem. Phys. Lett..

[71] Weinhold F., Klein R.A. (2014). Anti-Electrostatic Hydrogen Bonds. Angew. Chem. Int. Ed..

[72] Frenking G., Caramori G.F. (2015). No Need for a Re-examination of the Electrostatic Notation of the Hydrogen Bonding: A Comment. Angew. Chem. Int. Ed..

[73] Mata I., Molins E., Alkorta I., Espinosa E. (2015). The paradox of hydrogen-bonded anion-anion aggregates in oxoanions: A fundamental electrostatic problem explained in terms of electrophilic...nucleophilic interactions. J. Phys. Chem. A.

[74] Wang G., Chen Z., Xu Z., Wang J., Yang Y., Cai T., Shi J., Zhu W. (2016). Stability and Characteristics of the Halogen Bonding Interaction in an Anion-Anion Complex: A Computational Chemistry Study. J. Phys. Chem. B.

[75] Chalanchi S.M., Alkorta I., Elguero J., Quinonero D. (2017). Hydrogen Bond versus Halogen Bond in Cation-Cation Complexes: Effect of the Solvent. ChemPhysChem.

[76] Prohens R., Portell A., Font-Bardia M., Bauzá A., Frontera A. (2018). H-Bonded anion–anion complex trapped in a squaramido-based receptor. Chem. Comm..

[77] Iribarren Í., Montero-Campillo M.M., Alkorta I., Elguero J., Quiñonero D. (2019). Cations brought together by hydrogen bonds: The protonated pyridine–boronic acid dimer explained. Phys. Chem. Chem. Phys..

[78] Azofra L.M., Elguero J., Alkorta I. (2020). A Conceptual DFT Study of Phosphonate Dimers: Dianions Supported by H-Bonds. J. Phys. Chem. A.

[79] Azofra L.M., Elguero J., Alkorta I. (2020). Stabilisation of dianion dimers trapped inside cyanostar macrocycles. Phys. Chem. Chem. Phys.

[80] Chesman A.S.R., Hodgson J.L., Izgorodina E.I., Urbatsch A., Turner D.R., Deacon G.B., Batten S.R. (2014). Anion-Anion Interactions in the Crystal Packing of Functionalized Methanide Anions: An Experimental and Computational Study. Crys.Growth Des..

[81] Martínez-Camarena Á., Savastano M., Bazzicalupi C., Bianchi A., García-España E. (2020). Stabilisation of Exotic Tribromide (Br_3_^−)^ Anions via Supramolecular Interaction with a Tosylated Macrocyclic Pyridinophane. A Serendipitous Case. Molecules.

[82] Alkorta I., Mata I., Molins E., Espinosa E. (2016). Charged versus Neutral Hydrogen-Bonded Complexes: Is There a Difference in the Nature of the Hydrogen Bonds?. Chem. Eur. J..

[83] Quinonero D., Alkorta I., Elguero J. (2016). Cation-cation and anion-anion complexes stabilized by halogen bonds. Phys. Chem. Chem. Phys.

[84] Varadwaj A., Varadwaj P.R., Yamashita K. (2018). Do surfaces of positive electrostatic potential on different halogen derivatives in molecules attract? like attracting like!. J. Comput. Chem..

[85] Varadwaj P.R., Varadwaj A., Marques H.M., Yamashita K. (2018). Can Combined Electrostatic and Polarization Effects Alone Explain the F⋯F Negative-Negative Bonding in Simple Fluoro-Substituted Benzene Derivatives? A First-Principles Perspective. Computation.

[86] Varadwaj A., Marques H.M., Varadwaj P.R. (2019). Is the Fluorine in Molecules Dispersive? Is Molecular Electrostatic Potential a Valid Property to Explore Fluorine-Centered Non-Covalent Interactions?. Molecules.

[87] Dau T.M., Asamoah B.D., Belyaev A., Chakkaradhari G., Hirva P., Jänis J., Grachova E.V., Tunik S.P., Koshevoy I.O. (2016). Adjustable coordination of a hybrid phosphine–phosphine oxide ligand in luminescent Cu, Ag and Au complexes. Dalton Trans..

[88] Lu W., Kinjo R. (2018). Coordination of Asymmetric Diborenes towards Cationic Coinage Metals (Au, Ag, Cu). Chem. Eur. J..

[89] Rozas I., Alkorta I., Elguero J. (2000). Behavior of Ylides Containing N, O, and C Atoms as Hydrogen Bond Acceptors. J. Am. Chem. Soc..

[90] Cremer D., Kraka E. (1984). A description of the chemical bond in terms of local properties of electron density and energy. Croat. Chem. Acta.

[91] Mata I., Alkorta I., Molins E., Espinosa E. (2010). Universal Features of the Electron Density Distribution in Hydrogen-Bonding Regions: A Comprehensive Study Involving H⋅⋅⋅X (X=H, C, N, O, F, S, Cl, π) Interactions. Chem. Eur. J..

[92] Sanchez-Sanz G., Alkorta I., Elguero J. (2011). Theoretical study of the HXYH dimers (X, Y = O, S, Se). Hydrogen bonding and chalcogen-chalcogen interactions. Mol. Phys..

[93] Alkorta I., Solimannejad M., Provasi P., Elguero J. (2007). Theoretical Study of Complexes and Fluoride Cation Transfer between N_2_F^+^ and Electron Donors. J. Phys. Chem. A.

[94] Mathivathanan L., Al-Ameed K., Lazarou K., Trávníček Z., Sanakis Y., Herchel R., McGrady J.E., Raptis R.G. (2015). A trigonal prismatic Cu6-pyrazolato complex containing a μ6-F ligand. Dalton Trans..

[95] Shi K., Mathivathanan L., Raptis R.G. (2017). Crystal structure of [mu]6-chlorido-nonakis([mu]-4-chloropyrazolato)bis-[mu]3-methoxo-hexacopper(II). Acta Crystallogr. E.

[96] Mezei G., Raptis R.G. (2004). Effect of pyrazole-substitution on the structure and nuclearity of Cu(II)-pyrazolato complexes. Inorg. Chim. Acta.

[97] Mezei G., Raptis R.G., Telser J. (2006). Trinuclear, Antiferromagnetically Coupled CuII Complex with an EPR Spectrum of Mononuclear CuII:  Effect of Alcoholic Solvents. Inorg. Chem..

[98] Angaridis P.A., Baran P., Boča R., Cervantes-Lee F., Haase W., Mezei G., Raptis R.G., Werner R. (2002). Synthesis and Structural Characterization of Trinuclear CuII−Pyrazolato Complexes Containing μ3-OH, μ3-O, and μ3-Cl Ligands. Magnetic Susceptibility Study of [PPN]2[(μ3-O)Cu3(μ-pz)3Cl3]. Inorg. Chem..

[99] Surmann S.A., Mezei G. (2016). Halogen-bonded network of trinuclear copper(II) 4-iodopyrazolate complexes formed by mutual breakdown of chloroform and nanojars. Acta Crystallogr. E.

[100] Kamiyama A., Kajiwara T., Ito T. (2002). Cage-Type Hexacopper(II) Complex Formed by Chloride Template. Chem. Lett..

[101] Boudalis A.K., Rogez G., Heinrich B., Raptis R.G., Turek P. (2017). Towards ionic liquids with tailored magnetic properties: Bmim+ salts of ferro- and antiferromagnetic CuII3 triangles. Dalton Trans..

[102] Boča R., Dlháň L., Mezei G., Ortiz-Pérez T., Raptis R.G., Telser J. (2003). Triangular, Ferromagnetically-Coupled CuII3−Pyrazolato Complexes as Possible Models of Particulate Methane Monooxygenase (pMMO). Inorg. Chem..

[103] Møller C., Plesset M.S. (1934). Note on an Approximation Treatment for Many-Electron Systems. Phys. Rev..

[104] Dunning T.H. (1989). Gaussian-Basis Sets for Use in Correlated Molecular Calculations .1. The Atoms Boron through Neon and Hydrogen. J. Chem. Phys..

[105] Peterson K.A., Puzzarini C. (2005). Systematically convergent basis sets for transition metals. II. Pseudopotential-based correlation consistent basis sets for the group 11 (Cu, Ag, Au) and 12 (Zn, Cd, Hg) elements. Theor. Chem. Acc..

[106] Frisch M.J., Trucks G.W., Schlegel H.B., Scuseria G.E., Robb M.A., Cheeseman J.R., Scalmani G., Barone V., Petersson G.A., Nakatsuji H. (2016). Gaussian 16 c01.

[107] Bader R.F.W. (1990). Atoms in Molecules: A Quantum Theory.

[108] Popelier P.L.A. (2000). Atoms In Molecules.

[109] Keith T.A. In TK Gristmill Software, Overland Park, KS, USA. aim.tkgristmill.com.

[110] Jmol: An open-Source Java Viewer for Chemical Structures in 3D. http://www.jmol.org/.

[111] Lu T., Chen F. (2012). Multiwfn: A multifunctional wavefunction analyzer. J. Comput. Chem..

[112] Groom C.R., Bruno I.J., Lightfoot M.P., Ward S.C. (2016). The Cambridge Structural Database. Acta Crystallogr. Sect. B.

