# Supplementary material for: Interaction between Trinuclear Regium Complexes of Pyrazolate and Anions, a Computational Study
Source: Int J Mol Sci. 2020 Oct 28;21(21):8036. doi: 10.3390/ijms21218036 (PMC7663457; doi:10.3390/ijms21218036)
Supplement: Supplementary file 1 [file ijms-21-08036-s001.pdf]

Supporting Information

A Computational Study of the Interaction of Trinuclear Regium Complexes of Pyrazolate with Anions

Ibon Alkorta, José Elguero, Cristina Trujillo and Goar Sánchez-Sanz

Pg. S2-19 Table S1. Molecular graph, electronic energy and geometry of the $(Pz\text{-}Au)_3{:}X^-{}_n$ complexes

Pg. S20-43 Table S2. Molecular graph, electronic energy and geometry of the $(Pz\text{-}Ag)_3{:}X^-{}_n$ complexes

Pg. S44-65 Table S3. Molecular graph, electronic energy and geometry of the $(Pz\text{-}Cu)_3{:}X^-{}_n$ complexes

Pg. S66-67 Table S4. Electron density properties at the intermolecular BCPs in the 1:1 complexes.

Pg. S68 Table S5 .Barriers (kJ mol^{-1}) and interhalogen distance (Å) in the maximum of the dissociation curve (Fig. S1) of the 1:2 apical complexes.

Pg. S69 Table S6. Interatomic distances (Å) of the anions in the 1:2 complexes.

Pg. S70 Table S7. Anion-Anion repulsion corrected binding energies (kJ mol^{-1}) in the 1:2 complexes.

Pg. S71-72 Table S8. Electron density properties at the intermolecular BCPs in the 1:2 complexes.

Pg. S73 Table S9. Interatomic distances (Å) of the anions in the 1:3 complexes.

Pg. S74 Table S10. Anion-Anion repulsion corrected binding energies (kJ/mol) in the 1:3 complexes.

Pg. S75 Table S11. Electron density properties at the intermolecular BCPs in the 1:3 complexes.

Pg. S76 Table S12. Anion-Metal distances in the CSD search.

Pg. S77. Figure S1. Energy profiles (kJ mol^{-1}) as a function of the X-X distance (Å) in the 1:2 apical complexes. Only one of the X anions in move further away from the $(Pz\text{-}M)_3$ system.

Pg. S78-79 Figure S2. Electron density at the BCP (au) vs. the interatomic distance (Å).

Table S1. Molecular graph, electronic energy and geometry of the $(Pz\text{-}Au)_3{:}X^{-}{}_n$ complexes.

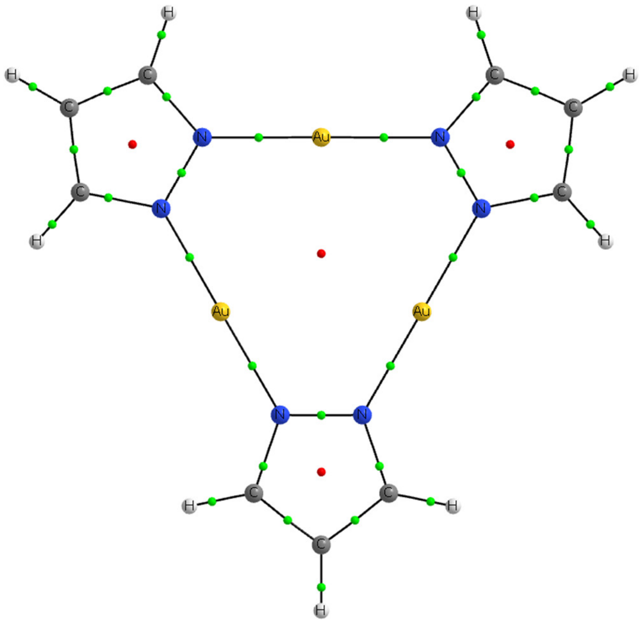	MP2= -1081.14994583 NIMAG= 0<br>X<br>X,1,r21<br>X,1,r21,2,120.<br>X,1,r21,2,120.,3,180.,0<br>N,2,r52,1,90.,3,0.,0<br>N,2,r52,1,90.,4,0.,0<br>N,3,r52,1,90.,2,0.,0<br>N,3,r52,1,90.,4,0.,0<br>N,4,r52,1,90.,3,0.,0<br>N,4,r52,1,90.,2,0.,0<br>C,5,r115,2,a1152,1,180.,0<br>C,6,r115,2,a1152,1,180.,0<br>C,7,r115,3,a1152,1,180.,0<br>C,8,r115,3,a1152,1,180.,0<br>C,9,r115,4,a1152,1,180.,0<br>C,10,r115,4,a1152,1,180.,0<br>C,1,r171,3,120.,2,0.,0<br>C,1,r171,4,120.,3,0.,0<br>C,1,r171,2,120.,4,0.,0<br>H,11,r2011,5,a20115,2,180.,0<br>H,12,r2011,6,a20115,2,180.,0<br>H,13,r2011,7,a20115,3,180.,0<br>H,14,r2011,8,a20115,3,180.,0<br>H,15,r2011,9,a20115,4,180.,0<br>H,16,r2011,10,a20115,4,180.,0<br>H,1,r261,3,120.,2,0.,0<br>H,1,r261,4,120.,3,0.,0<br>H,1,r261,2,120.,4,0.,0<br>Au,1,r291,3,60.,2,0.,0<br>Au,1,r291,4,60.,3,0.,0<br>Au,1,r291,2,60.,4,0.,0<br><br>r21=2.66728329<br>r52=0.68024126<br>r115=1.36591004<br>r171=4.81517842<br>r2011=1.08842895<br>r261=5.9035919<br>r291=1.92172936<br>a1152=108.5958088<br>a20115=119.93401813

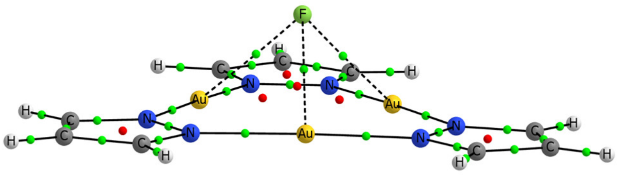	MP2= -1180.85680735 NIMAG= 0<br>N,0.6800672465,0.020703612,2.6772411945<br>N,-0.680095464,0.0207131258,2.6772512226<br>N,2.6586210257,0.0206972805,-0.7496551245<br>N,1.9785326059,0.0207548899,-1.9275787427<br>N,-1.9785217127,0.0207556484,-1.9275652164<br>N,-2.6586171652,0.0207078801,-0.7496434328<br>C,1.1147408995,0.0304221767,3.9714240123<br>C,-1.1147582901,0.0304488726,3.9714373215<br>C,3.9967489033,0.030356647,-1.0203278597<br>C,2.8819754952,0.0304540065,-2.9511228376<br>C,-2.8819643717,0.0304368725,-2.9511088262<br>C,-3.9967450028,0.0303532959,-1.0203171563<br>C,-0.0000040458,0.0334121437,4.8297495989<br>C,4.1826833671,0.0333523787,-2.4148937257<br>C,-4.1826745365,0.0333362176,-2.4148830743<br>H,2.182800768,0.0350775944,4.1802642143<br>H,-2.1828163869,0.0351310632,4.1802883734<br>H,4.7116521253,0.0349696889,-0.1997915025<br>H,2.5287952349,0.0351721305,-3.9805058387<br>H,-2.5287819455,0.0351462594,-3.9804911429<br>H,-4.7116493384,0.0349724337,-0.1997821784<br>H,0.0000025247,0.0364999114,5.9189289853<br>H,5.1259343636,0.036405956,-2.9594938798<br>H,-5.1259237452,0.0363813632,-2.9594865883<br>Au,1.662557897,-0.0089498858,0.9598766623<br>Au,0.0000025529,-0.0087043843,-1.919637215<br>Au,-1.6625461381,-0.0088957083,0.9598745037<br>F,-0.0000168667,-1.7001114701,-0.0000517468

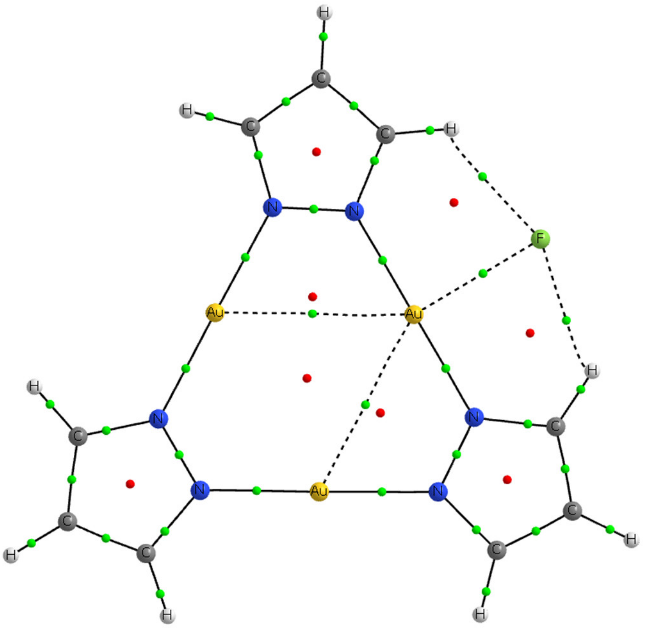	MP2= -1180.86175663 NIMAG= 0<br>N,-0.6805381842,0.,-2.901753165<br>N,0.6805381842,0.,-2.901753165<br>N,-2.7122653698,0.,0.4838306746<br>N,-1.9647988648,0.,1.6182925702<br>N,1.9647988648,0.,1.6182925702<br>N,2.7122653698,0.,0.4838306746<br>C,-1.1146071046,0.,-4.1970082244<br>C,1.1146071046,0.,-4.1970082244<br>C,-4.0343153338,0.,0.8370160136<br>C,-2.7866567754,0.,2.7035545746<br>C,2.7866567754,0.,2.7035545746<br>C,4.0343153338,0.,0.8370160136<br>C,0.,0.,-5.0538388065<br>C,-4.1188090253,0.,2.2428321966<br>C,4.1188090253,0.,2.2428321966<br>H,-2.1825935155,0.,-4.4065198157<br>H,2.1825935155,0.,-4.4065198157<br>H,-4.8038071091,0.,0.0663918324<br>H,-2.3088235269,0.,3.6870411416<br>H,2.3088235269,0.,3.6870411416<br>H,4.8038071091,0.,0.0663918324<br>H,0.,0.,-6.1426948987<br>H,-5.0269783083,0.,2.8442301969<br>H,5.0269783083,0.,2.8442301969<br>Au,-1.7063229334,0.,-1.2124144914<br>Au,0.,0.,1.6110242171<br>Au,1.7063229334,0.,-1.2124144914<br>F,0.,0.,4.0452749591

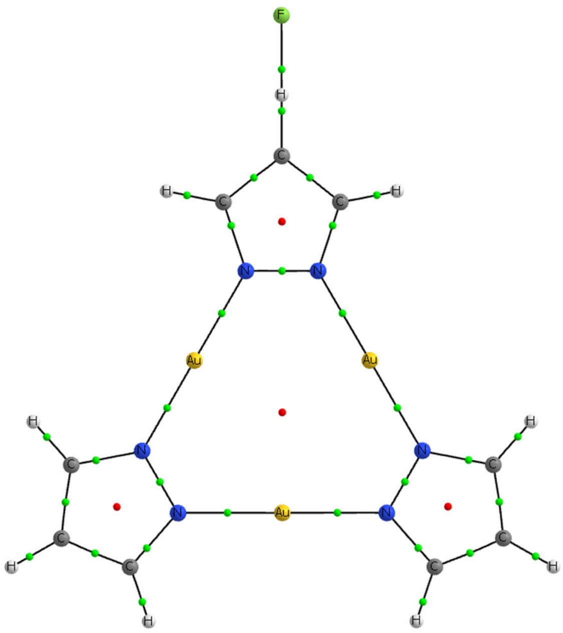

MP2= -1180.84901612 NIMAG= 0
N,-0.6817423849,0.,-10.9846909446
N,0.6817423849,0.,-10.9846909446
N,-2.6450200914,0.,-7.570781069
N,-1.9701430592,0.,-6.3902411174
N,1.9701430592,0.,-6.3902411174
N,2.6450200914,0.,-7.570781069
C,-1.1036318386,0.,-12.2929643051
C,1.1036318386,0.,-12.2929643051
C,-3.9830397822,0.,-7.3026797254
C,-2.8749881659,0.,-5.3656722725
C,2.8749881659,0.,-5.3656722725
C,3.9830397822,0.,-7.3026797254
C,0.,0.,-13.167228275
C,-4.1705610247,0.,-5.9087607527
C,4.1705610247,0.,-5.9087607527
H,-2.1732183065,0.,-12.502199928
H,2.1732183065,0.,-12.502199928
H,-4.6978362287,0.,-8.1235961284
H,-2.5268992704,0.,-4.3343555262
H,2.5268992704,0.,-4.3343555262
H,4.6978362287,0.,-8.1235961284
H,0.,0.,-14.3281221762
H,-5.1152255124,0.,-5.3677774477
H,5.1152255124,0.,-5.3677774477
Au,-1.6564911013,0.,-9.2853007195
Au,0.,0.,-6.3923842803
Au,1.6564911013,0.,-9.2853007195
F,0.,0.,-15.8361418537

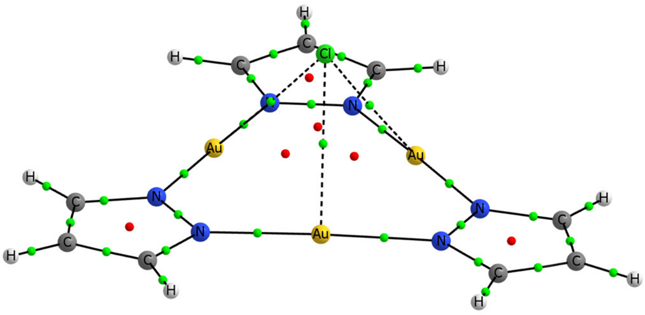

MP2= -1540.90104623 NIMAG= 0
N,0.6791334591,0.0409145135,2.6761106288
N,-0.6791501776,0.0408991688,2.6761255902
N,2.6571328069,0.0409235545,-0.7499190583
N,1.9780206023,0.0408520272,-1.9262518374
N,-1.9780230269,0.0408557384,-1.9262450601
N,-2.6571486988,0.0409127746,-0.7499170696
C,1.1151320133,0.0559309231,3.9696159772
C,-1.1151166048,0.0559084439,3.9696415395
C,3.9953457447,0.0559952982,-1.0190608908
C,2.8802851891,0.0559033678,-2.9505411115
C,-2.8802765797,0.055923383,-2.9505443855
C,-3.9953570613,0.0559928059,-1.0190786326

	C,0.0000179467,0.0630167916,4.8272089768<br>C,4.1805156178,0.0630737982,-2.4135672066<br>C,-4.1805122525,0.0630873388,-2.4135866222<br>H,2.1831228715,0.0569284301,4.1790195581<br>H,-2.1831024408,0.0568815921,4.1790708547<br>H,4.7106758671,0.0570321441,-0.1988427799<br>H,2.5276890563,0.0568408038,-3.9801673074<br>H,-2.5276700727,0.0568740461,-3.9801672169<br>H,-4.7106962147,0.0570189297,-0.1988687233<br>H,0.0000308826,0.0672048695,5.9162705407<br>H,5.1236880471,0.0672933134,-2.9580678348<br>H,-5.1236785517,0.067313222,-2.9580979009<br>Au,1.6566973497,0.0049542163,0.9564907548<br>Au,-0.0000023911,0.0047202939,-1.9132023361<br>Au,-1.6567541426,0.0049052654,0.9565217335<br>Cl,0.000000761,-2.3281570539,0.0000498196
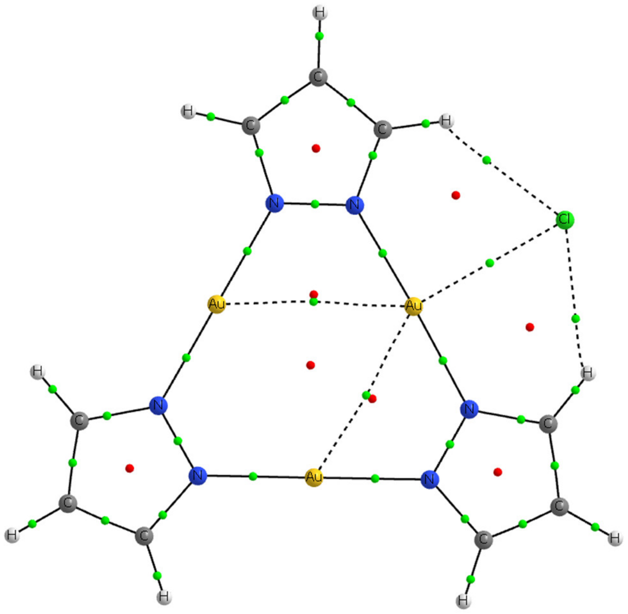	MP2= -1540.90658571 NIMAG= 0<br>N,-0.6800295939,0.,-2.900904195<br>N,0.6800295939,0.,-2.900904195<br>N,-2.6755074668,0.,0.4990747209<br>N,-1.974810961,0.,1.6656834373<br>N,1.974810961,0.,1.6656834373<br>N,2.6755074668,0.,0.4990747209<br>C,-1.1152629553,0.,-4.1954744323<br>C,1.1152629553,0.,-4.1954744323<br>C,-4.0117139144,0.,0.7916056519<br>C,-2.8519101834,0.,2.7094948231<br>C,2.8519101834,0.,2.7094948231<br>C,4.0117139144,0.,0.7916056519<br>C,0.,0.,-5.0515590849<br>C,-4.1605125647,0.,2.1900271864<br>C,4.1605125647,0.,2.1900271864<br>H,-2.1831261166,0.,-4.4057981779<br>H,2.1831261166,0.,-4.4057981779<br>H,-4.7440168782,0.,-0.0144258131<br>H,-2.4435372761,0.,3.7224037654<br>H,2.4435372761,0.,3.7224037654<br>H,4.7440168782,0.,-0.0144258131<br>H,0.,0.,-6.1403279546<br>H,-5.0929455418,0.,2.7525490093<br>H,5.0929455418,0.,2.7525490093<br>Au,-1.6786380996,0.,-1.197140686<br>Au,0.,0.,1.6962735372

	Au,1.6786380996,0.,-1.197140686<br>Cl,0.,0.,4.6521754008
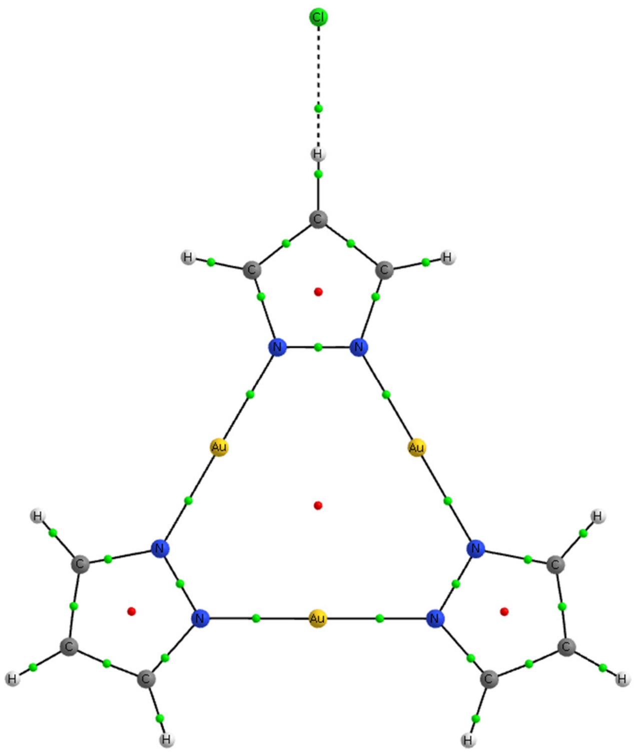	MP2= -1540.89064026 NIMAG= 0<br>N,-0.6813723354,0.,-10.9619819163<br>N,0.6813723354,0.,-10.9619819163<br>N,-2.6454968029,0.,-7.5483528014<br>N,-1.9700539529,0.,-6.3680210556<br>N,1.9700539529,0.,-6.3680210556<br>N,2.6454968029,0.,-7.5483528014<br>C,-1.1099880385,0.,-12.2642207561<br>C,1.1099880385,0.,-12.2642207561<br>C,-3.9839844357,0.,-7.2809302459<br>C,-2.8754330233,0.,-5.3441366658<br>C,2.8754330233,0.,-5.3441366658<br>C,3.9839844357,0.,-7.2809302459<br>C,0.,0.,-13.1247662051<br>C,-4.1708466642,0.,-5.8874204229<br>C,4.1708466642,0.,-5.8874204229<br>H,-2.1768670309,0.,-12.4816004725<br>H,2.1768670309,0.,-12.4816004725<br>H,-4.6988958259,0.,-8.1017221205<br>H,-2.5277622086,0.,-4.3127190274<br>H,2.5277622086,0.,-4.3127190274<br>H,4.6988958259,0.,-8.1017221205<br>H,0.,0.,-14.2276318657<br>H,-5.1154776823,0.,-5.346509899<br>H,5.1154776823,0.,-5.346509899<br>Au,-1.6583011531,0.,-9.2604460865<br>Au,0.,0.,-6.3687095013<br>Au,1.6583011531,0.,-9.2604460865<br>Cl,0.,0.,-16.5446860632

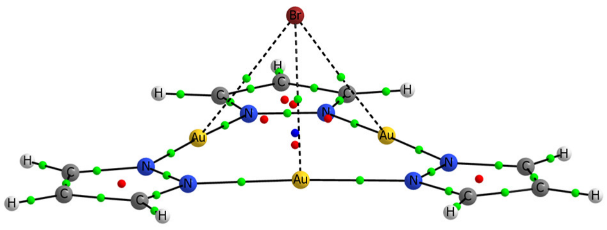	MP2= -1496.89392137 NIMAG= 0<br>N,0.6790790892,0.0441667356,2.6764815116<br>N,-0.6790438176,0.0441885625,2.6764636728<br>N,2.6574329683,0.0441593065,-0.7501488635<br>N,1.9783705054,0.0441484121,-1.9263198118<br>N,-1.9783761278,0.0441511438,-1.9263296663<br>N,-2.6574264654,0.0441712254,-0.7501539364<br>C,1.1151915874,0.0622167338,3.9698900571<br>C,-1.1151929162,0.062275825,3.9698573218<br>C,3.9956145716,0.062178103,-1.0191561349<br>C,2.8804177812,0.0622851773,-2.950725146

<table>
<tr><td></td><td>C,-2.8804305287,0.0622400209,-2.9507289929
C,-3.9956118529,0.0621969962,-1.0191451916
C,-0.0000132154,0.0712658848,4.8272752646
C,4.1805457366,0.071260564,-2.4136430009
C,-4.1805538903,0.0712298817,-2.4136330946
H,2.1831693482,0.0627609479,4.179370444
H,-2.1831772571,0.0628752128,4.1793053581
H,4.7110056696,0.0627079819,-0.1989895608
H,2.5278236272,0.0629140782,-3.9803536402
H,-2.5278435449,0.0628317387,-3.9803603786
H,-4.7109977733,0.0627502617,-0.1989740611
H,-0.0000285735,0.0774969336,5.9163278347
H,5.1236910163,0.0775078276,-2.9581719932
H,-5.1237037935,0.077453385,-2.9581546161
Au,1.6546940839,0.0033546417,0.9553364625
Au,0.0000004386,0.0033692737,-1.9106406359
Au,-1.6546275076,0.0034061066,0.9553014204
Br,-0.0000091593,-2.4715629615,0.0000193774</td></tr>
<tr><td>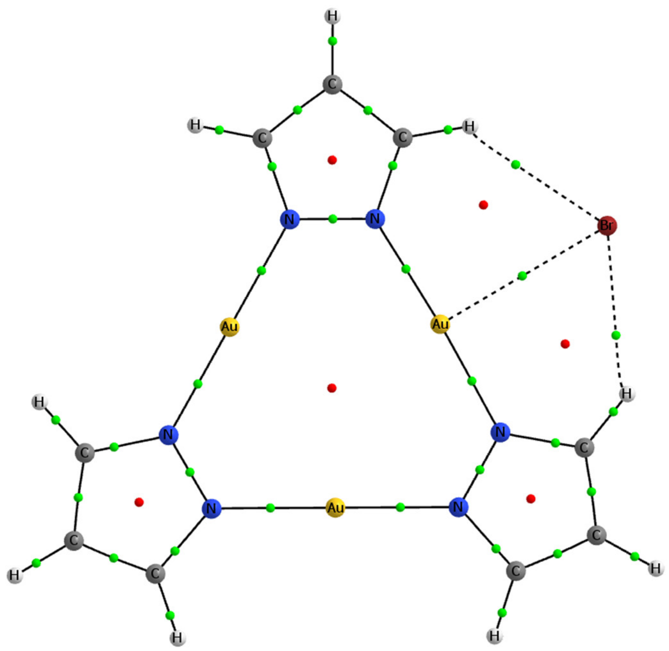</td><td>MP2= -1496.89712782 NIMAG= 0
N,-0.6799415778,0.,-2.9041356498
N,0.6799415778,0.,-2.9041356498
N,-2.6647602565,0.,0.500187961
N,-1.977698692,0.,1.6752873451
N,1.977698692,0.,1.6752873451
N,2.6647602565,0.,0.500187961
C,-1.1154601618,0.,-4.1984920349
C,1.1154601618,0.,-4.1984920349
C,-4.0046532122,0.,0.7753721996
C,-2.87011039,0.,2.7067754625
C,2.87011039,0.,2.7067754625
C,4.0046532122,0.,0.7753721996
C,0.,0.,-5.0543777982
C,-4.1716473105,0.,2.1711684112
C,4.1716473105,0.,2.1711684112
H,-2.1832913314,0.,-4.4090257566
H,2.1832913314,0.,-4.4090257566
H,-4.7259684313,0.,-0.0405062555
H,-2.4809834614,0.,3.7265672563
H,2.4809834614,0.,3.7265672563
H,4.7259684313,0.,-0.0405062555
H,0.,0.,-6.1431297378
H,-5.1106122131,0.,2.7225981654
H,5.1106122131,0.,2.7225981654</td></tr>
</table>

	Au,-1.6696137474,0.,-1.195647327<br>Au,0.,0.,1.720120019<br>Au,1.6696137474,0.,-1.195647327<br>Br,0.,0.,4.8078404413
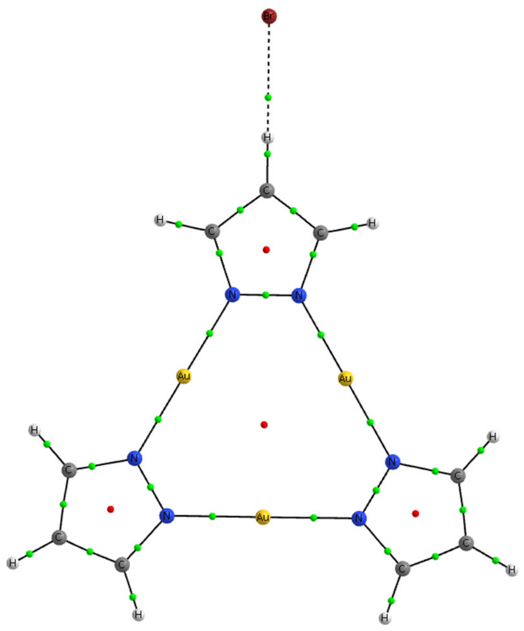	MP2= -1496.88058761 NIMAG= 0<br>N,-0.6813253646,0.,-10.9556529581<br>N,0.6813253646,0.,-10.9556529581<br>N,-2.6456935127,0.,-7.5422218716<br>N,-1.9700219437,0.,-6.3619681489<br>N,1.9700219437,0.,-6.3619681489<br>N,2.6456935127,0.,-7.5422218716<br>C,-1.110692007,0.,-12.2570548574<br>C,1.110692007,0.,-12.2570548574<br>C,-3.9841658867,0.,-7.2747882984<br>C,-2.8753366232,0.,-5.3381525634<br>C,2.8753366232,0.,-5.3381525634<br>C,3.9841658867,0.,-7.2747882984<br>C,0.,0.,-13.1161399088<br>C,-4.1708017307,0.,-5.8813133964<br>C,4.1708017307,0.,-5.8813133964<br>H,-2.1773302748,0.,-12.4752413468<br>H,2.1773302748,0.,-12.4752413468<br>H,-4.6992931158,0.,-8.0953920697<br>H,-2.5277677101,0.,-4.3067023202<br>H,2.5277677101,0.,-4.3067023202<br>H,4.6992931158,0.,-8.0953920697<br>H,0.,0.,-14.2153091476<br>H,-5.1153582886,0.,-5.3403102295<br>H,5.1153582886,0.,-5.3403102295<br>Au,-1.6587344432,0.,-9.2539238953<br>Au,0.,0.,-6.3626603619<br>Au,1.6587344432,0.,-9.2539238953<br>Br,0.,0.,-16.7223632501

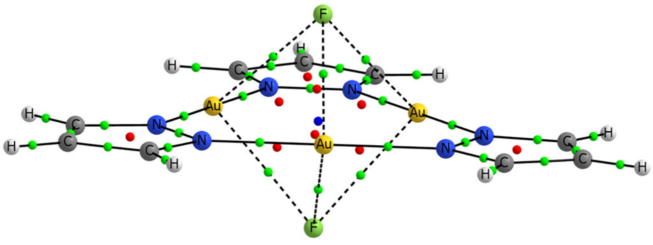	MP2= -1280.43876196 NIMAG= 0<br>N,0.680801075,0.0000406471,2.6877643192<br>N,-0.6807698315,0.0000411467,2.687742417<br>N,2.6680693575,0.0000407693,-0.7542951074<br>N,1.9872696181,0.0000415996,-1.9334423943<br>N,-1.987273836,0.0000420215,-1.9334505207<br>N,-2.668059839,0.0000417293,-0.7543011606<br>C,1.1139576191,-0.0000251536,3.9823463441<br>C,-1.1139678772,-0.0000286708,3.9823111553

	C,4.0057890355,-0.0000232722,-1.0264568545<br>C,2.891803457,-0.0000290332,-2.9558839041<br>C,-2.8918183103,-0.0000249281,-2.9558823558<br>C,-4.0057842087,-0.0000232565,-1.0264423616<br>C,-0.000019002,0.0000029403,4.8460881301<br>C,4.1968284494,0.000003037,-2.4230593196<br>C,-4.1968379807,0.0000024247,-2.423042945<br>H,2.1829850712,-0.0000447475,4.1869902976<br>H,-2.1830015169,-0.0000514707,4.1869217983<br>H,4.7175255824,-0.000041509,-0.2029707301<br>H,2.5344902965,-0.0000526033,-3.9840018799<br>H,-2.5345141068,-0.0000456594,-3.9840030488<br>H,-4.7175124353,-0.0000424794,-0.2029486278<br>H,-0.0000362438,-0.0000021423,5.9368029583<br>H,5.1414087385,-0.0000022734,-2.9684277263<br>H,-5.1414244494,-0.0000041071,-2.968400908<br>Au,1.6672768816,0.000047114,0.9626033234<br>Au,-0.0000042749,0.0000481888,-1.9251569303<br>Au,-1.6672334028,0.0000477198,0.962571627<br>F,0.0000258279,-1.7671969703,0.0000121021<br>F,0.0000263054,1.7672389389,0.0000123023
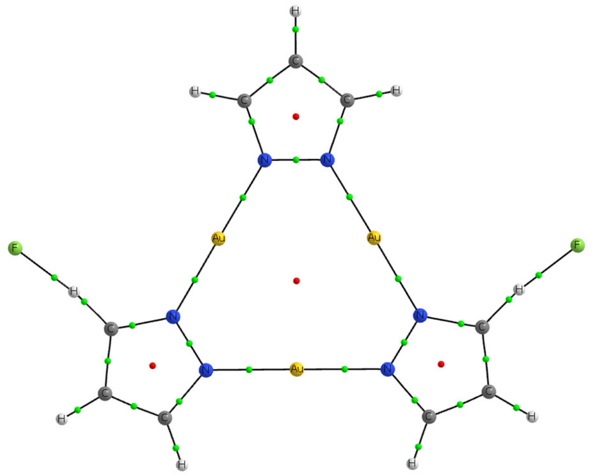	MP2= -1280.49543687 NIMAG= 0<br>N,0.8110494397,0.0009357636,2.6314489576<br>N,-0.5560072514,0.0005511404,2.5995958816<br>N,2.7482441568,0.0004781782,-0.8012985781<br>N,2.0680368612,-0.0004804328,-1.9794276147<br>N,-1.8733901846,-0.0009441908,-2.0180940749<br>N,-2.5293363916,-0.0005965742,-0.8182648933<br>C,1.2596909374,0.0008968791,3.9267948815<br>C,-1.0032576064,0.0003520185,3.8920897097<br>C,4.0870094962,0.0006729443,-1.0698518248<br>C,2.9699768079,-0.0006879893,-3.0045698388<br>C,-2.7708654841,-0.0008564559,-3.0543075284<br>C,-3.8722920461,-0.0003532781,-1.0771866565<br>C,0.1109931762,0.0004679965,4.7489572372<br>C,4.2694786577,-0.0000155611,-2.4650281732<br>C,-4.0572301868,-0.0005239205,-2.4705928735<br>H,2.3656089943,0.0012286548,4.2168200694<br>H,-2.075143005,0.0001974573,4.0881048577<br>H,4.7993215774,0.0013585471,-0.2460967329<br>H,2.6127145185,-0.0013738005,-4.0333206619<br>H,-2.4690294809,-0.0011494178,-4.1570632178<br>H,-4.5779911728,-0.000200862,-0.2469166115

	H,0.1059506772,0.0003593825,5.838531812<br>H,5.2126563048,-0.0000184226,-3.0095839055<br>H,-5.0033506744,-0.0004508993,-3.0110130793<br>Au,1.7807547389,0.0007475611,0.9225631244<br>Au,0.0913937432,-0.0007244466,-2.0034927909<br>Au,-1.5334333899,-0.0000342421,0.8853289813<br>F,3.8282555425,0.001239914,4.8442881858<br>F,-2.2808435487,-0.0010759437,-5.7374404209
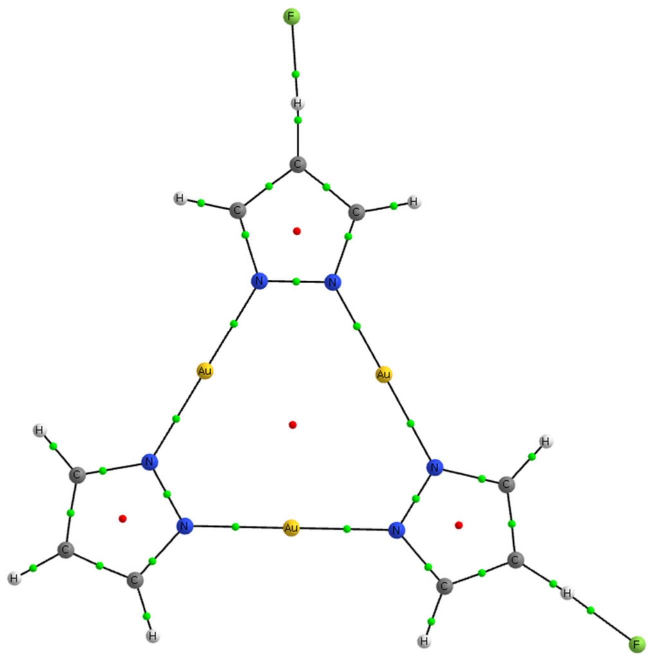	MP2= -1280.49691184 NIMAG= 0<br>N,0.6797695543,0.,2.6247512181<br>N,-0.6797695543,0.,2.6247512181<br>N,2.6530586093,0.,-0.7840201386<br>N,1.9735077384,0.,-1.9654095233<br>N,-1.9735077384,0.,-1.9654095233<br>N,-2.6530586093,0.,-0.7840201386<br>C,1.1157371754,0.,3.9189321511<br>C,-1.1157371754,0.,3.9189321511<br>C,3.9987441967,0.,-1.0627757567<br>C,2.8999013703,0.,-2.9790886579<br>C,-2.8999013703,0.,-2.9790886579<br>C,-3.9987441967,0.,-1.0627757567<br>C,0.,0.,4.775532096<br>C,4.2083214005,0.,-2.4540273994<br>C,-4.2083214005,0.,-2.4540273994<br>H,2.1839552952,0.,4.1284608808<br>H,-2.1839552952,0.,4.1284608808<br>H,4.7120422376,0.,-0.2390412002<br>H,2.5496945308,0.,-4.0109710322<br>H,-2.5496945308,0.,-4.0109710322<br>H,-4.7120422376,0.,-0.2390412002<br>H,0.,0.,5.8645075721<br>H,5.2099943509,0.,-2.9981302799<br>H,-5.2099943509,0.,-2.9981302799<br>Au,1.6681122205,0.,0.9104484719<br>Au,0.,0.,-1.967448798<br>Au,-1.6681122205,0.,0.9104484719<br>F,6.6800955163,0.,-3.6754241619<br>F,-6.6800955163,0.,-3.6754241619

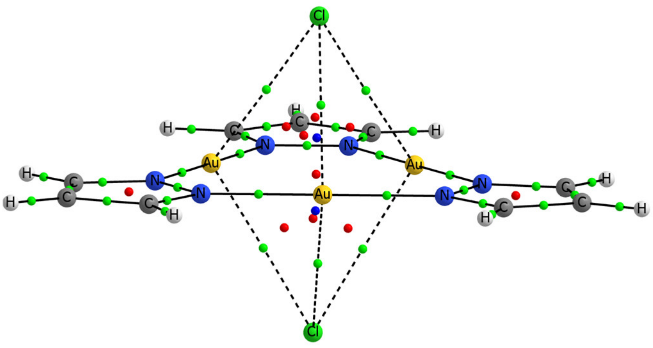	MP2= -2000.54233916 NIMAG= 0<br>N,0.6785309248,0.0003302024,2.6789712236<br>N,-0.6785344739,0.0003290561,2.6789732484<br>N,2.659350029,0.0003290934,-0.7518481976<br>N,1.9808034823,0.0003290921,-1.927103221<br>N,-1.9808037444,0.0003291708,-1.9271095307<br>N,-2.6593390742,0.0003286271,-0.7518538834<br>C,1.1146007668,-0.0000477272,3.9718196556<br>C,-1.1146125066,-0.0000481927,3.9718193347<br>C,3.9970235422,-0.0000502917,-1.0206350747<br>C,2.8823999683,-0.0000484631,-2.9511844214<br>C,-2.8824058055,-0.0000449124,-2.9511863222<br>C,-3.99701388,-0.0000531031,-1.0206309493<br>C,-0.000006813,-0.0002911325,4.8331485481<br>C,4.1856395537,-0.0002932144,-2.4165793417<br>C,-4.1856420839,-0.0002920691,-2.4165735405<br>H,2.1833068222,-0.0000762689,4.1781341891<br>H,-2.1833185108,-0.0000770926,4.1781300793<br>H,4.7100585622,-0.0000800736,-0.1982735793<br>H,2.5267143972,-0.0000768117,-3.9798660211<br>H,-2.5267266871,-0.0000699783,-3.9798701746<br>H,-4.7100409899,-0.0000856077,-0.1982629463<br>H,-0.0000071352,-0.0006106709,5.9232601136<br>H,5.1296989183,-0.0006137176,-2.9616430653<br>H,-5.1297062396,-0.0006116459,-2.9616293345<br>Au,1.6535157977,0.0004485543,0.9546538862<br>Au,0.0000008864,0.0004476528,-1.9092965694<br>Au,-1.653495878,0.0004473604,0.9546445105<br>Cl,0.000005191,-2.5993979209,-0.0000037783<br>Cl,0.00000498,2.5995500848,-0.0000048376

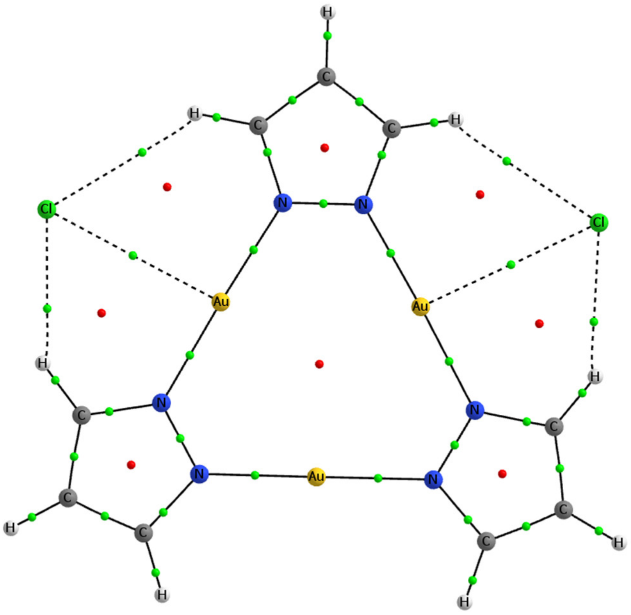	MP2= -2000.58638473 NIMAG= 0<br>N,0.679844882,0.,2.6645211497<br>N,-0.681662146,0.,2.6750318589<br>N,2.6537616825,0.,-0.7459004026<br>N,1.9728495386,0.,-1.9252748313<br>N,-1.9676205636,0.,-1.9210235133<br>N,-2.6574766187,0.,-0.7471791942<br>C,1.1402074536,0.,3.9474736577<br>C,-1.1022962184,0.,3.9770683242<br>C,3.9931574115,0.,-1.007086633<br>C,2.8687413137,0.,-2.9546324432<br>C,-2.8485087415,0.,-2.9611854493<br>C,-3.9953903105,0.,-1.0339176345<br>C,0.0291514412,0.,4.8144956461<br>C,4.1670570042,0.,-2.4058514831<br>C,-4.1548998154,0.,-2.4324937117<br>H,2.2236709548,0.,4.1019994462<br>H,-2.1682756859,0.,4.2006186325<br>H,4.6871343892,0.,-0.1640810516<br>H,2.4856655535,0.,-3.9771369262<br>H,-2.4406002494,0.,-3.9767552596<br>H,-4.7219802903,0.,-0.2225274899<br>H,0.0426637282,0.,5.9039298866<br>H,5.1106732541,0.,-2.950648579<br>H,-5.0916213998,0.,-2.9889128158<br>Au,1.6909049046,0.,0.9721813211<br>Au,0.0035187311,0.,-1.9504572633<br>Au,-1.6698370054,0.,0.9640808446<br>Cl,4.4658659502,0.,2.8043829841<br>Cl,-0.1957339311,0.,-5.2697448549

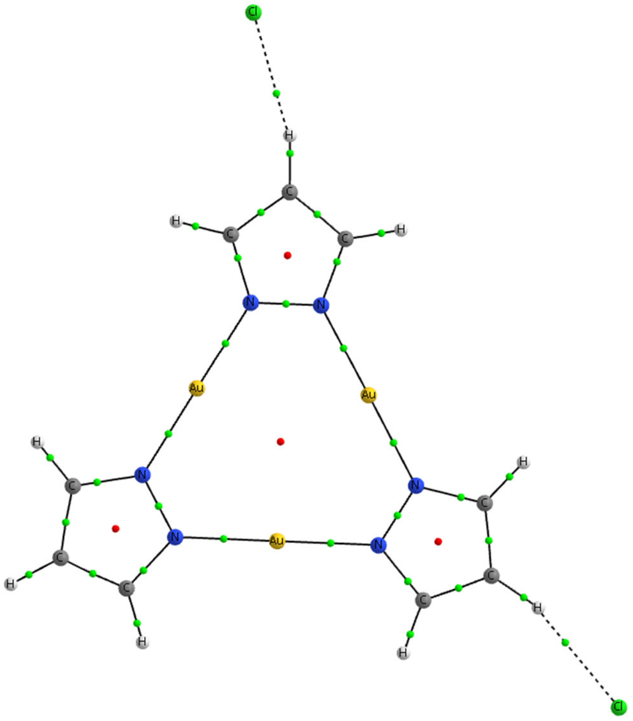	MP2= -2000.58945267 NIMAG= 0<br>N,0.6798275862,0.,2.6059666934<br>N,-0.6798275862,0.,2.6059666934<br>N,2.6524598057,0.,-0.8032712627<br>N,1.9725453828,0.,-1.9838947602<br>N,-1.9725453828,0.,-1.9838947602<br>N,-2.6524598057,0.,-0.8032712627<br>C,1.1158976055,0.,3.9000387726<br>C,-1.1158976055,0.,3.9000387726<br>C,3.9955995458,0.,-1.0751908367<br>C,2.8903879858,0.,-3.0015818101<br>C,-2.8903879858,0.,-3.0015818101<br>C,-3.9955995458,0.,-1.0751908367<br>C,0.,0.,4.7556351544<br>C,4.1906058329,0.,-2.4654578701<br>C,-4.1906058329,0.,-2.4654578701<br>H,2.1839443694,0.,4.1102560926<br>H,-2.1839443694,0.,4.1102560926<br>H,4.7182899512,0.,-0.261135622<br>H,2.5434327854,0.,-4.0336615929<br>H,-2.5434327854,0.,-4.0336615929<br>H,-4.7182899512,0.,-0.261135622<br>H,0.,0.,5.8444029805<br>H,5.1608168895,0.,-2.9803102775<br>H,-5.1608168895,0.,-2.9803102775<br>Au,1.66689024,0.,0.8941438039<br>Au,0.,0.,-1.9861368901<br>Au,-1.66689024,0.,0.8941438039<br>Cl,7.591113127,0.,-3.4628519509<br>Cl,-7.591113127,0.,-3.4628519509

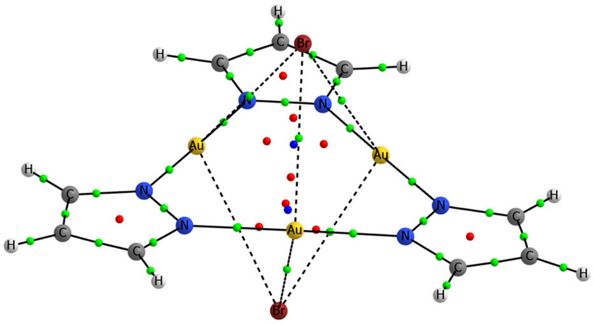	MP2= -1912.53243794 NIMAG= 0<br>N,0.6783435936,-0.0001340144,2.6788140394<br>N,-0.6783394473,-0.0001322153,2.6788112177<br>N,2.6590856906,-0.0001235768,-0.751954584<br>N,1.9807566943,-0.000117549,-1.9268792413<br>N,-1.980761903,-0.0001167982,-1.9268734286<br>N,-2.6590977213,-0.0001286461,-0.7519508221<br>C,1.114776051,0.000027442,3.9714324148<br>C,-1.1147699011,0.0000071632,3.9714289798<br>C,3.9967463756,0.0000469967,-1.0202858381<br>C,2.8819972877,0.0000229508,-2.9511350362<br>C,-2.8819930104,-0.0000251285,-2.9511359636

	C,-3.9967560911,0.0000871162,-1.0202855144<br>C,0.0000029875,0.0000943106,4.8322873768<br>C,4.1849032482,0.0001152348,-2.4161315852<br>C,-4.1849013853,0.0001102815,-2.4161341614<br>H,2.1834471119,0.0000512079,4.1778131506<br>H,-2.1834406132,0.0000121366,4.1778071788<br>H,4.7098012946,0.0000735097,-0.1979710099<br>H,2.5264013141,0.0000275493,-3.9798234914<br>H,-2.5263922479,-0.0000614881,-3.979822922<br>H,-4.7098159221,0.0001539003,-0.197973756<br>H,0.0000029009,0.000204246,5.9223122338<br>H,5.1288986643,0.0002296685,-2.9611346486<br>H,-5.1288936438,0.0002233463,-2.9611413547<br>Au,1.6504818999,-0.0001578454,0.9528997361<br>Au,-0.0000033128,-0.0001470967,-1.9058459253<br>Au,-1.6504790816,-0.0001587563,0.952894484<br>Br,0.0000001176,-2.7416251275,-0.000019256<br>Br,-0.000000951,2.7414411814,-0.0000022727
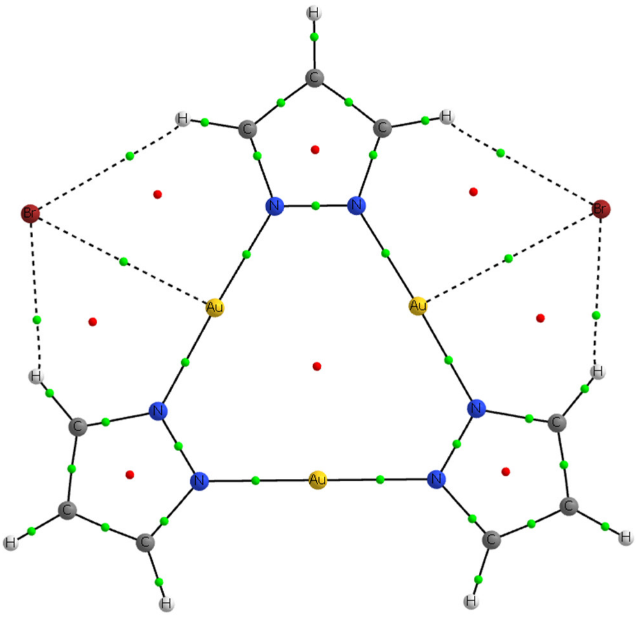	MP2= -1912.57058889 NIMAG= 0<br>N,0.6788265939,0.,2.6714444986<br>N,-0.6826812415,0.,2.6741653113<br>N,2.6498344328,0.,-0.7433273148<br>N,1.9686575543,0.,-1.9231602773<br>N,-1.9741255036,0.,-1.9236033245<br>N,-2.6572357143,0.,-0.7458633578<br>C,1.128938146,0.,3.9583618181<br>C,-1.1124373799,0.,3.9729870007<br>C,3.989456589,0.,-1.0053628925<br>C,2.8653980994,0.,-2.9522893071<br>C,-2.8635728188,0.,-2.9568700228<br>C,-3.9969263614,0.,-1.0230944693<br>C,0.0127954484,0.,4.8180176191<br>C,4.1629379957,0.,-2.4034733724<br>C,-4.1661279299,0.,-2.420089993<br>H,2.2089632766,0.,4.1282435834<br>H,-2.1799410458,0.,4.1888723187<br>H,4.6857744044,0.,-0.1645603222<br>H,2.4854006217,0.,-3.9757195095<br>H,-2.4706821779,0.,-3.9771401053<br>H,-4.7176403641,0.,-0.206551835<br>H,0.0198746446,0.,5.9073987589<br>H,5.1064469711,0.,-2.9482085334<br>H,-5.1060200732,0.,-2.9709113267

	Au,1.6909096052,0.,0.9775995718<br>Au,-0.0011712613,0.,-1.9531704595<br>Au,-1.6658560965,0.,0.9617824657<br>Br,4.6071927542,0.,2.8308873143<br>Br,-0.1480239522,0.,-5.4053896224
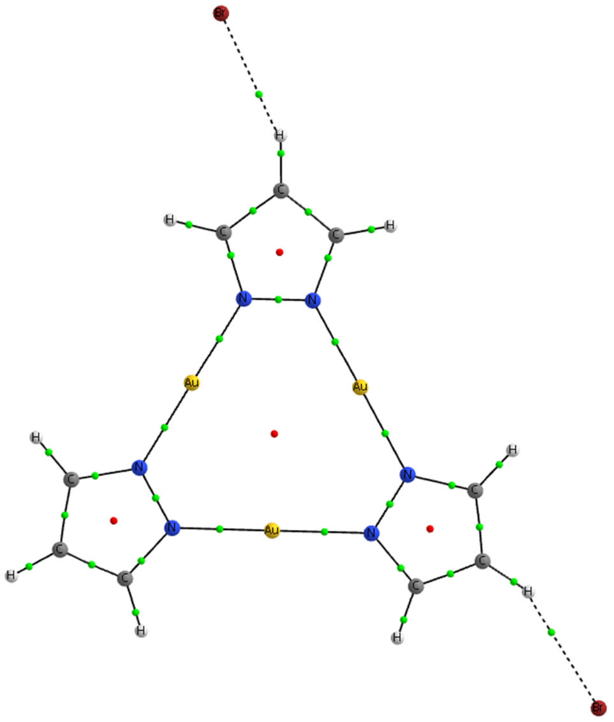	MP2= -1912.57112067 NIMAG= 0<br>N,0.6798731457,0.,2.5800906973<br>N,-0.6798731457,0.,2.5800906973<br>N,2.6542162787,0.,-0.828334765<br>N,1.9721604397,0.,-2.0077635252<br>N,-1.9721604397,0.,-2.0077635252<br>N,-2.6542162787,0.,-0.828334765<br>C,1.1159287103,0.,3.8741531808<br>C,-1.1159287103,0.,3.8741531808<br>C,3.9965665053,0.,-1.100971359<br>C,2.8871191642,0.,-3.0274678956<br>C,-2.8871191642,0.,-3.0274678956<br>C,-3.9965665053,0.,-1.100971359<br>C,0.,0.,4.7295617914<br>C,4.1867672155,0.,-2.491476628<br>C,-4.1867672155,0.,-2.491476628<br>H,2.1839667005,0.,4.084443362<br>H,-2.1839667005,0.,4.084443362<br>H,4.7241893787,0.,-0.2914294956<br>H,2.5379982211,0.,-4.0587453654<br>H,-2.5379982211,0.,-4.0587453654<br>H,-4.7241893787,0.,-0.2914294956<br>H,0.,0.,5.8182948907<br>H,5.1560172542,0.,-3.0006458446<br>H,-5.1560172542,0.,-3.0006458446<br>Au,1.6681040204,0.,0.8694980197<br>Au,0.,0.,-2.0089299144<br>Au,-1.6681040204,0.,0.8694980197<br>Br,7.8494902795,0.,-3.1208137654<br>Br,-7.8494902795,0.,-3.1208137654

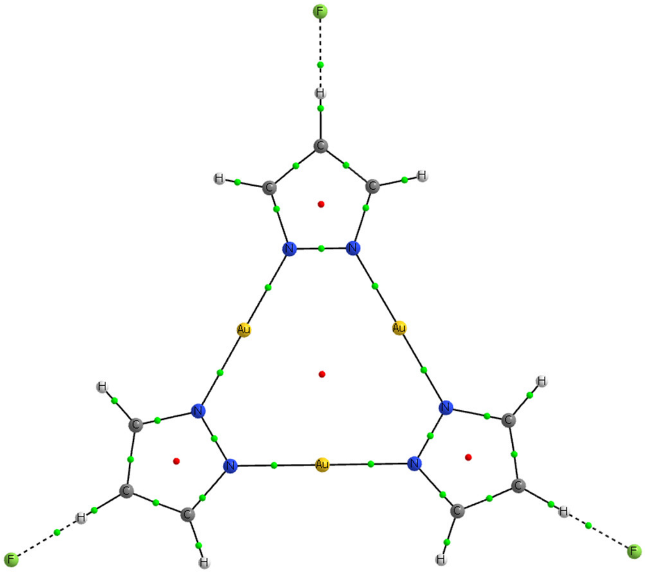	MP2= -1380.09592555 NIMAG= 0<br>X<br>X,1,r21<br>X,1,r21,2,120.<br>X,1,r21,2,120.,3,180.,0<br>N,2,r52,1,90.,3,0.,0<br>N,2,r52,1,90.,4,0.,0<br>N,3,r52,1,90.,2,0.,0<br>N,3,r52,1,90.,4,0.,0<br>N,4,r52,1,90.,3,0.,0<br>N,4,r52,1,90.,2,0.,0<br>C,5,r115,2,a1152,1,180.,0<br>C,6,r115,2,a1152,1,180.,0<br>C,7,r115,3,a1152,1,180.,0<br>C,8,r115,3,a1152,1,180.,0<br>C,9,r115,4,a1152,1,180.,0<br>C,10,r115,4,a1152,1,180.,0<br>C,1,r171,3,120.,2,0.,0<br>C,1,r171,4,120.,3,0.,0<br>C,1,r171,2,120.,4,0.,0<br>H,11,r2011,5,a20115,2,180.,0<br>H,12,r2011,6,a20115,2,180.,0<br>H,13,r2011,7,a20115,3,180.,0<br>H,14,r2011,8,a20115,3,180.,0<br>H,15,r2011,9,a20115,4,180.,0<br>H,16,r2011,10,a20115,4,180.,0<br>H,1,r261,3,120.,2,0.,0<br>H,1,r261,4,120.,3,0.,0<br>H,1,r261,2,120.,4,0.,0<br>Au,1,r291,3,60.,2,0.,0<br>Au,1,r291,4,60.,3,0.,0<br>Au,1,r291,2,60.,4,0.,0<br>F,1,rf,29,60.,2,180.,0<br>F,1,rf,30,60.,3,180.,0<br>F,1,rf,31,60.,4,180.,0<br><br>r21=2.67223497<br>r52=0.68119758<br>r115=1.37313143<br>r171=4.8522309<br>r2011=1.08955811<br>r261=5.97840667<br>r291=1.9254901

	a1152=107.98694139<br>a20115=119.02515627<br>rf=7.71367068
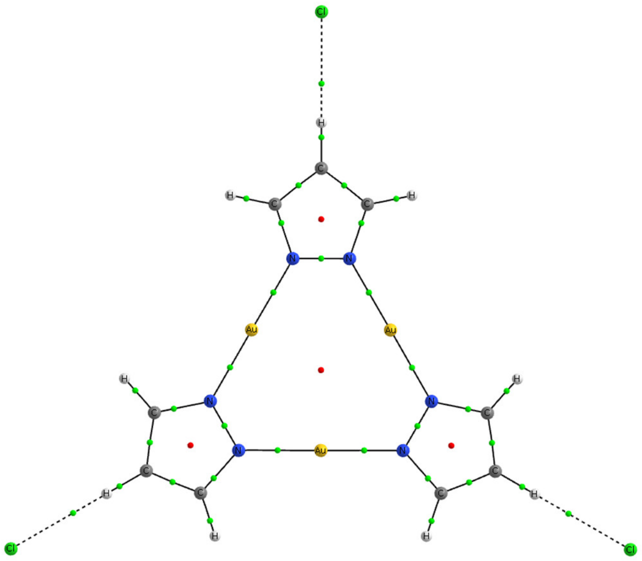	MP2= -2460.24657873 NIMAG= 0<br>N,0.6809545795,0.,2.6708887047<br>N,-0.6809545795,0.,2.6708887047<br>N,2.6535343801,0.,-0.7457160323<br>N,1.9725707821,0.,-1.9251550129<br>N,-1.9725707821,0.,-1.9251550129<br>N,-2.6535343801,0.,-0.7457160323<br>C,1.1104970093,0.,3.9718823012<br>C,-1.1104970093,0.,3.9718823012<br>C,3.9949963361,0.,-1.0242276463<br>C,2.884482493,0.,-2.9476558113<br>C,-2.884482493,0.,-2.9476558113<br>C,-3.9949963361,0.,-1.0242276463<br>C,0.,0.,4.8345613364<br>C,4.1868381436,0.,-2.4172886348<br>C,-4.1868381436,0.,-2.4172886348<br>H,2.1779046615,0.,4.1862413399<br>H,-2.1779046615,0.,4.1862413399<br>H,4.7143456034,0.,-0.2070098031<br>H,2.5364101413,0.,-3.9792348591<br>H,-2.5364101413,0.,-3.9792348591<br>H,-4.7143456034,0.,-0.2070098031<br>H,0.,0.,5.931778746<br>H,5.1370516344,0.,-2.9659053312<br>H,-5.1370516344,0.,-2.9659053312<br>Au,1.6666696574,0.,0.9622554284<br>Au,0.,0.,-1.9244509373<br>Au,-1.6666696574,0.,0.9622554284<br>Cl,7.4330396688,0.,-4.2915000533<br>Cl,-7.4330396688,0.,-4.2915000533<br>Cl,0.,0.,8.5829626372

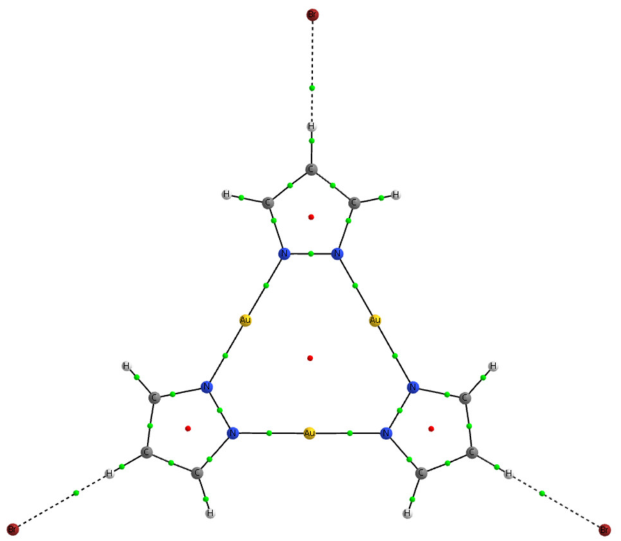

MP2= -2328.22079785 NIMAG= 0
N,0.6809022065,0.,2.6706431794
N,-0.6809022065,0.,2.6706431794
N,2.6532521419,0.,-0.7456156703
N,1.972361616,0.,-1.9249712234
N,-1.972361616,0.,-1.9249712234
N,-2.6532521419,0.,-0.7456156703
C,1.111158652,0.,3.9709343812
C,-1.111158652,0.,3.9709343812
C,3.9944678786,0.,-1.0231373157
C,2.8833283452,0.,-2.9477237733
C,-2.8833283452,0.,-2.9477237733
C,-3.9944678786,0.,-1.0231373157
C,0.,0.,4.832264871
C,4.1848387154,0.,-2.416090735
C,-4.1848387154,0.,-2.416090735
H,2.178382374,0.,4.1858492613
H,-2.178382374,0.,4.1858492613
H,4.7141934085,0.,-0.2063440108
H,2.535852057,0.,-3.9794301828
H,-2.535852057,0.,-3.9794301828
H,-4.7141934085,0.,-0.2063440108
H,0.,0.,5.927579303
H,5.1334136388,0.,-2.9637394148
H,-5.1334136388,0.,-2.9637394148
Au,1.6665568846,0.,0.9622098837
Au,0.,0.,-1.9243494063
Au,-1.6665568846,0.,0.9622098837
Br,7.613167262,0.,-4.3959369323
Br,-7.613167262,0.,-4.3959369323
Br,0.,0.,8.7912113296

Table S2. Molecular graph, electronic energy and geometry of the (Pz-Ag)$_3$:X$^-{}_n$ complexes.

<table>
<tr><td>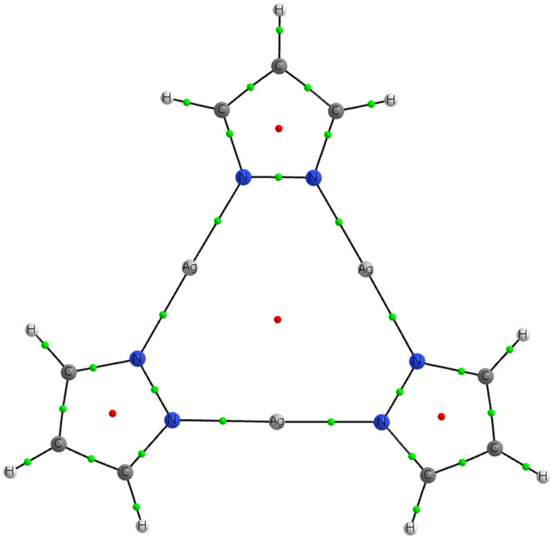
</td><td>MP2= -1114.42179833 NIMAG= 0
X
X,1,r21
X,1,r21,2,120.
X,1,r21,2,120.,3,180.,0
N,2,r52,1,90.,3,0.,0
N,2,r52,1,90.,4,0.,0
N,3,r52,1,90.,2,0.,0
N,3,r52,1,90.,4,0.,0
N,4,r52,1,90.,3,0.,0
N,4,r52,1,90.,2,0.,0
C,5,r115,2,a1152,1,180.,0
C,6,r115,2,a1152,1,180.,0
C,7,r115,3,a1152,1,180.,0
C,8,r115,3,a1152,1,180.,0
C,9,r115,4,a1152,1,180.,0
C,10,r115,4,a1152,1,180.,0
C,1,r171,3,120.,2,0.,0
C,1,r171,4,120.,3,0.,0
C,1,r171,2,120.,4,0.,0
H,11,r2011,5,a20115,2,180.,0
H,12,r2011,6,a20115,2,180.,0
H,13,r2011,7,a20115,3,180.,0
H,14,r2011,8,a20115,3,180.,0
H,15,r2011,9,a20115,4,180.,0
H,16,r2011,10,a20115,4,180.,0
H,1,r261,3,120.,2,0.,0
H,1,r261,4,120.,3,0.,0
H,1,r261,2,120.,4,0.,0
Ag,1,r291,3,60.,2,0.,0
Ag,1,r291,4,60.,3,0.,0
Ag,1,r291,2,60.,4,0.,0

r21=2.7498381
r52=0.68417589
r115=1.36645246
r171=4.90601527
r2011=1.08948985
r261=5.99465491
r291=1.97902891
a1152=108.26155634
a20115=120.12601866</td></tr>
</table>

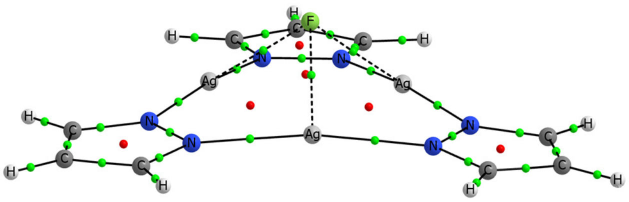

MP2= -1214.16221409 NIMAG= 0

N,0.6845284454,2.8042020019,0.0421704418

N,-0.684526555,2.8042021985,0.0421715786

N,2.7707743953,-0.8092817168,0.0421704776

N,2.0862473603,-1.994918628,0.0421709719

N,-2.0862471865,-1.9949205114,0.0421687052

N,-2.7707745391,-0.8092833154,0.0421703206

C,1.1110672261,4.0981657838,0.1374009299

C,-1.1110666698,4.0981647808,0.137418986

C,4.1046491968,-1.0868701725,0.1374066072

C,2.9935817886,-3.0112944084,0.1374181977

C,-2.9935828094,-3.011295924,0.1374074125

C,-4.1046495614,-1.086870289,0.1374051493

C,0.0000001169,4.9617570267,0.1975247899

C,4.2970081392,-2.4808783707,0.1975225257

C,-4.2970086809,-2.4808786975,0.1975172272

H,2.1784734139,4.3185487358,0.1561605331

H,-2.1784728878,4.3185464551,0.156196709

H,4.8292091753,-0.2726606238,0.156170899

H,2.6507349467,-4.0458862111,0.1561942778

H,-2.6507367519,-4.045888069,0.1561776038

	H,-4.8292089073,-0.2726601621,0.1561734535<br>H,-0.00000013,6.0491520773,0.2722216543<br>H,5.2387199518,-3.0245760572,0.272217255<br>H,-5.2387207482,-3.0245759886,0.2722112075<br>Ag,1.6702750741,0.9643341767,-0.1147617396<br>Ag,0.0000003317,-1.928665709,-0.1147559912<br>Ag,-1.6702723759,0.9643322821,-0.1147530868<br>F,-0.0000017587,-0.000000671,-1.5068433766
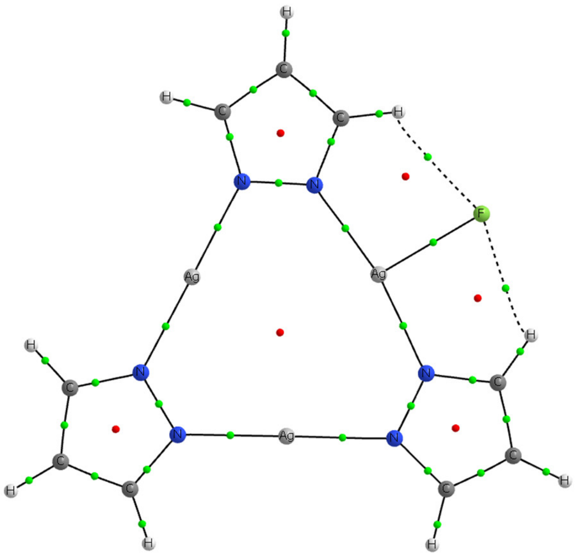	MP2= -1214.14614086 NIMAG= 0<br>N,-0.6834987362,0.,-2.9761170683<br>N,0.6834987362,0.,-2.9761170683<br>N,-2.821161438,0.,0.5078527881<br>N,-2.0678216748,0.,1.6492588191<br>N,2.0678216748,0.,1.6492588191<br>N,2.821161438,0.,0.5078527881<br>C,-1.1115199447,0.,-4.2736926823<br>C,1.1115199447,0.,-4.2736926823<br>C,-4.1441178715,0.,0.8617716116<br>C,-2.9060491826,0.,2.7228451794<br>C,2.9060491826,0.,2.7228451794<br>C,4.1441178715,0.,0.8617716116<br>C,0.,0.,-5.135767117<br>C,-4.2407397777,0.,2.2665202801<br>C,4.2407397777,0.,2.2665202801<br>H,-2.178578322,0.,-4.4944663742<br>H,2.178578322,0.,-4.4944663742<br>H,-4.9188089716,0.,0.0942224103<br>H,-2.4553216712,0.,3.7178573913<br>H,2.4553216712,0.,3.7178573913<br>H,4.9188089716,0.,0.0942224103<br>H,0.,0.,-6.2249525323<br>H,-5.1509032781,0.,2.8659655643<br>H,5.1509032781,0.,2.8659655643<br>Ag,-1.7507342177,0.,-1.2233459218<br>Ag,0.,0.,1.829050381<br>Ag,1.7507342177,0.,-1.2233459218

	F,0.,0.,4.0850777521
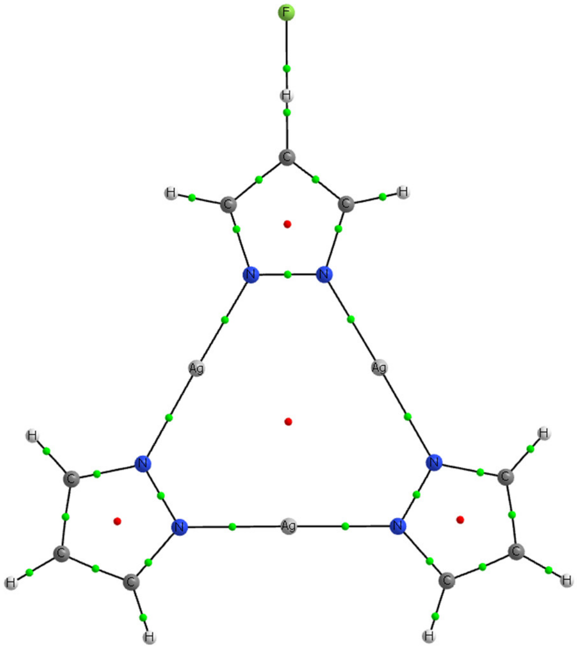	MP2= -1214.11580417 NIMAG= 0<br>N,-0.6862934234,0.,-11.0536548133<br>N,0.6862934234,0.,-11.0536548133<br>N,-2.7227886354,0.,-7.5330468534<br>N,-2.0413937235,0.,-6.3472561119<br>N,2.0413937235,0.,-6.3472561119<br>N,2.7227886354,0.,-7.5330468534<br>C,-1.1004084604,0.,-12.3648142217<br>C,1.1004084604,0.,-12.3648142217<br>C,-4.0593208349,0.,-7.255584776<br>C,-2.950887713,0.,-5.3265221376<br>C,2.950887713,0.,-5.3265221376<br>C,4.0593208349,0.,-7.255584776<br>C,0.,0.,-13.2433493619<br>C,-4.2505103053,0.,-5.8617984367<br>C,4.2505103053,0.,-5.8617984367<br>H,-2.169220503,0.,-12.5841318783<br>H,2.169220503,0.,-12.5841318783<br>H,-4.7838428552,0.,-8.0693241122<br>H,-2.6108541603,0.,-4.2912872004<br>H,2.6108541603,0.,-4.2912872004<br>H,4.7838428552,0.,-8.0693241122<br>H,0.,0.,-14.394599871<br>H,-5.1945262767,0.,-5.3190206376<br>H,5.1945262767,0.,-5.3190206376<br>Ag,-1.7090763326,0.,-9.3135118754<br>Ag,0.,0.,-6.3559214431<br>Ag,1.7090763326,0.,-9.3135118754<br>F,0.,0.,-15.9481424453

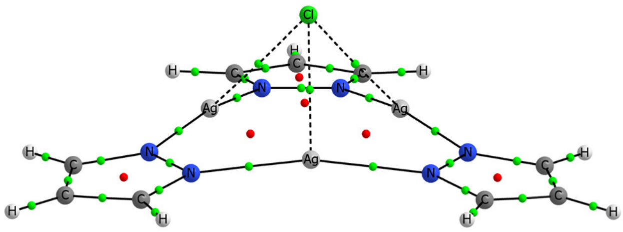	MP2= -1574.19903106 NIMAG= 0<br>N,0.6839192464,2.8001336842,0.024029476<br>5<br>N,-<br>0.6839155829,2.8001290572,0.0240455591<br>N,2.7669445954,-<br>0.8077740859,0.0240258614<br>N,2.0830253936,-<br>1.9923527544,0.0240288953<br>N,-2.0830263646,-<br>1.9923553868,0.0240354405

	N,-2.7669430716,-0.8077767967,0.0240417649<br>C,1.1114686414,4.0885396839,0.1735523904<br>C,-1.1114679643,4.088544489,0.1734789895<br>C,4.0965181585,-1.0817127979,0.173482467<br>C,2.9850439317,-3.0068290795,0.1735142785<br>C,-2.9850443511,-3.0068303836,0.1735323119<br>C,-4.0965174147,-1.0817135881,0.1734950531<br>C,-0.0000008222,4.9483007653,0.2695626533<br>C,4.2853527451,-2.4741510912,0.2695501305<br>C,-4.2853540377,-2.4741520727,0.2695570768<br>H,2.1788241702,4.3085496242,0.1996860126<br>H,-2.1788240978,4.308559391,0.1995435204<br>H,4.8207354565,-0.2673638349,0.1995632093<br>H,2.6418994988,-4.0411913281,0.1996256249<br>H,-2.6418998475,-4.0411923126,0.1996488928<br>H,-4.8207336259,-0.267363669,0.199576549<br>H,-0.0000020685,6.0318760785,0.3868884585<br>H,5.223755621,-3.0159401418,0.3868761316<br>H,-5.2237577836,-3.0159405623,0.3868794658<br>Ag,1.6550622361,0.9555522823,-0.1978392707<br>Ag,-0.0000010233,-1.9110913519,-0.1978272832

	Ag,-1.6550545057,0.9555442535,-0.1978173302<br>Cl,-0.0000071331,0.0000019217,-2.1860826093
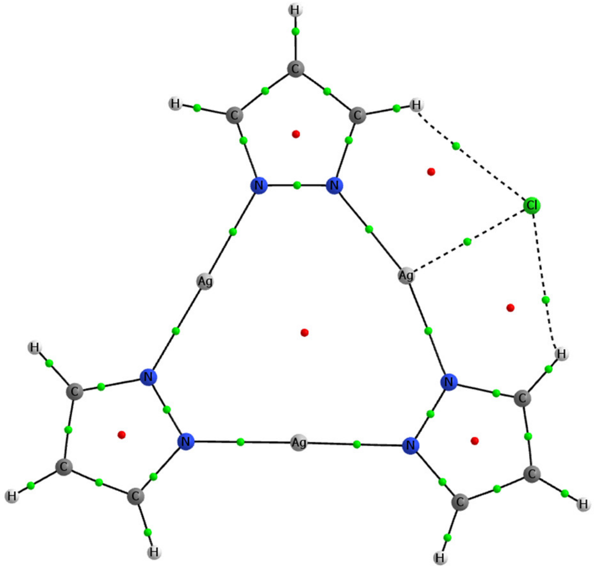	MP2= -1574.18904016 NIMAG= 0<br>N,-0.6833251219,0.,-2.9692456622<br>N,0.6833251219,0.,-2.9692456622<br>N,-2.7638998472,0.,0.5359922855<br>N,-2.0872335055,0.,1.7258839995<br>N,2.0872335055,0.,1.7258839995<br>N,2.7638998472,0.,0.5359922855<br>C,-1.112178376,0.,-4.2662456945<br>C,1.112178376,0.,-4.2662456945<br>C,-4.1088191725,0.,0.7959001435<br>C,-3.0050224915,0.,2.7344188175<br>C,3.0050224915,0.,2.7344188175<br>C,4.1088191725,0.,0.7959001435<br>C,0.,0.,-5.1275182469<br>C,-4.3044372289,0.,2.1885259553<br>C,4.3044372289,0.,2.1885259553<br>H,-2.1791341243,0.,-4.4873262999<br>H,2.1791341243,0.,-4.4873262999<br>H,-4.8267648508,0.,-0.0250595036<br>H,-2.6444070248,0.,3.7643505735<br>H,2.6444070248,0.,3.7643505735<br>H,4.8267648508,0.,-0.0250595036<br>H,0.,0.,-6.2166286351<br>H,-5.2528205387,0.,2.7250549727<br>H,5.2528205387,0.,2.7250549727<br>Ag,-1.7113395525,0.,-1.1978890657<br>Ag,0.,0.,2.0318295468<br>Ag,1.7113395525,0.,-1.1978890657<br>Cl,0.,0.,4.6543487698

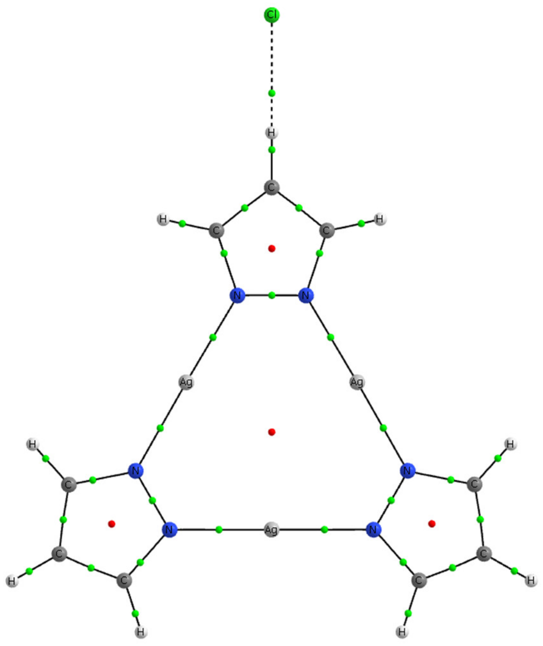

MP2= -1574.15919944 NIMAG= 0
N,-0.6856840736,0.,-11.0328638744
N,0.6856840736,0.,-11.0328638744
N,-2.7225595204,0.,-7.5087480286
N,-2.0408672471,0.,-6.3229482327
N,2.0408672471,0.,-6.3229482327
N,2.7225595204,0.,-7.5087480286
C,-1.1064446174,0.,-12.3381959093
C,1.1064446174,0.,-12.3381959093
C,-4.0594712743,0.,-7.2318026651
C,-2.9508274138,0.,-5.302840778
C,2.9508274138,0.,-5.302840778
C,4.0594712743,0.,-7.2318026651
C,0.,0.,-13.2039544404
C,-4.2502519215,0.,-5.8383534486
C,4.2502519215,0.,-5.8383534486
H,-2.1724918723,0.,-12.5653311538
H,2.1724918723,0.,-12.5653311538
H,-4.7841969491,0.,-8.0453467611
H,-2.6113232884,0.,-4.2674717821
H,2.6113232884,0.,-4.2674717821
H,4.7841969491,0.,-8.0453467611
H,0.,0.,-14.3051284283
H,-5.1942445204,0.,-5.2956972788
H,5.1942445204,0.,-5.2956972788
Ag,-1.7113574517,0.,-9.2879941689
Ag,0.,0.,-6.3260491668
Ag,1.7113574517,0.,-9.2879941689
Cl,0.,0.,-16.6715963889

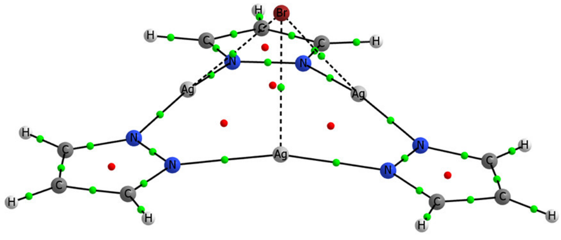

MP2= -1530.19018428 NIMAG= 0
N,0.6838514134,-0.0746029469,2.7998313808
N,-0.6838538219,-0.0746031547,2.799832474
N,2.7666522175,-0.0746016052,-0.8076827988
N,2.0827996199,-0.0745973849,-1.9921502441
N,-2.0827993148,-0.0745970385,-1.9921487977
N,-2.7666528484,-0.0746007975,-0.8076817176
C,1.1115985496,0.0917318681,4.0861026149
C,-1.1115983037,0.0917276911,4.0861050942
C,4.0944672405,0.0917501615,-1.0803781579
C,2.9828729653,0.0917201855,-3.0057256805
C,-2.9828712746,0.0917271656,-3.0057244436
C,-4.094468064,0.0917466948,-1.0803786672

	C,0.0000008919,0.1989460471,4.9444537249<br>C,4.2820231192,0.198951225,-2.47222652<br>C,-4.2820225653,0.1989507437,-2.4722269151<br>H,2.1789512697,0.1204163544,4.3059167202<br>H,-2.1789506144,0.1204079026,4.3059215127<br>H,4.8185069626,0.1204474483,-0.2659300283<br>H,2.6395643335,0.1203887801,-4.039988815<br>H,-2.6395612287,0.1204015176,-4.0399869204<br>H,-4.8185089623,0.1204396003,-0.2659314376<br>H,0.0000019028,0.3300717882,6.0264628105<br>H,5.2190706387,0.3300775574,-3.0132305794<br>H,-5.219069767,0.3300760113,-3.0132318126<br>Ag,1.6486505574,-0.3206982665,0.9518486223<br>Ag,0.0000001653,-0.3206936447,-1.9036994699<br>Ag,-1.6486539607,-0.3206968676,0.9518502211<br>Br,-0.0000011215,-2.4502870355,-0.0000021695
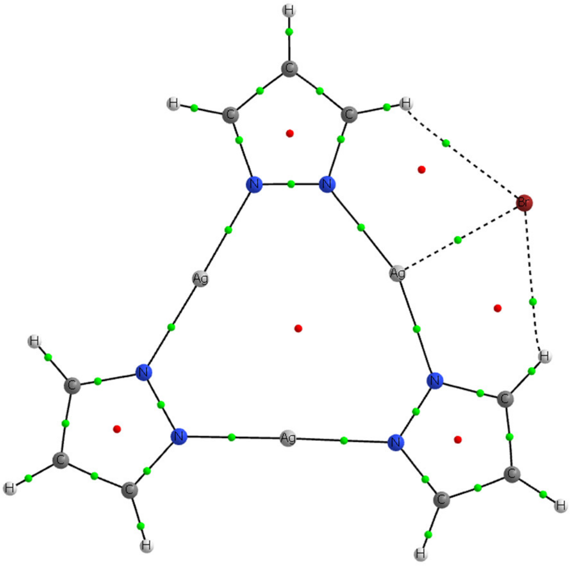	MP2= -1530.17909977 NIMAG= 0<br>N,-0.6832654207,0.,-2.972035821<br>N,0.6832654207,0.,-2.972035821<br>N,-2.7479078067,0.,0.5396675271<br>N,-2.0920254668,0.,1.7414005827<br>N,2.0920254668,0.,1.7414005827<br>N,2.7479078067,0.,0.5396675271<br>C,-1.1123713467,0.,-4.2688724104<br>C,1.1123713467,0.,-4.2688724104<br>C,-4.0975947407,0.,0.7745819789<br>C,-3.0300705368,0.,2.7316974133<br>C,3.0300705368,0.,2.7316974133<br>C,4.0975947407,0.,0.7745819789<br>C,0.,0.,-5.1299365448<br>C,-4.3192635575,0.,2.1628392081<br>C,4.3192635575,0.,2.1628392081<br>H,-2.1792972672,0.,-4.4900212035<br>H,2.1792972672,0.,-4.4900212035<br>H,-4.7999855152,0.,-0.0597674736<br>H,-2.6934849008,0.,3.7693379561<br>H,2.6934849008,0.,3.7693379561<br>H,4.7999855152,0.,-0.0597674736<br>H,0.,0.,-6.2190360851<br>H,-5.2770275092,0.,2.682338107<br>H,5.2770275092,0.,2.682338107<br>Ag,-1.6990965176,0.,-1.1947430262<br>Ag,0.,0.,2.0923742588

	Ag,1.6990965176,0.,-1.1947430262<br>Br,0.,0.,4.8145051728
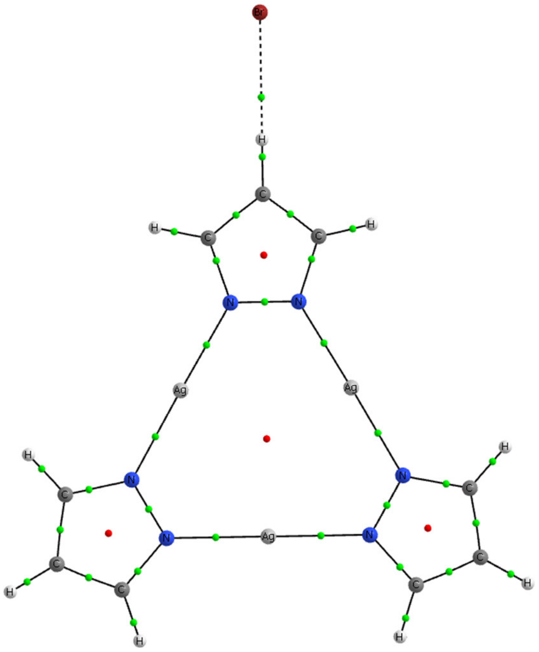	MP2= -1530.14938477 NIMAG= 0<br>N,-0.6856232851,0.,-11.0269202899<br>N,0.6856232851,0.,-11.0269202899<br>N,-2.7225435465,0.,-7.5023755717<br>N,-2.0408323992,0.,-6.3165210514<br>N,2.0408323992,0.,-6.3165210514<br>N,2.7225435465,0.,-7.5023755717<br>C,-1.1071291941,0.,-12.3314858688<br>C,1.1071291941,0.,-12.3314858688<br>C,-4.0595196231,0.,-7.2255166244<br>C,-2.9508719393,0.,-5.2965539889<br>C,2.9508719393,0.,-5.2965539889<br>C,4.0595196231,0.,-7.2255166244<br>C,0.,0.,-13.1958470574<br>C,-4.2502648684,0.,-5.8321289678<br>C,4.2502648684,0.,-5.8321289678<br>H,-2.1729415999,0.,-12.5592955383<br>H,2.1729415999,0.,-12.5592955383<br>H,-4.7843038795,0.,-8.0390117519<br>H,-2.6114527296,0.,-4.2611650215<br>H,2.6114527296,0.,-4.2611650215<br>H,4.7843038795,0.,-8.0390117519<br>H,0.,0.,-14.2938450907<br>H,-5.1942538977,0.,-5.2894915585<br>H,5.1942538977,0.,-5.2894915585<br>Ag,-1.71166461,0.,-9.2813016475<br>Ag,0.,0.,-6.3192655303<br>Ag,1.71166461,0.,-9.2813016475<br>Br,0.,0.,-16.8494231353

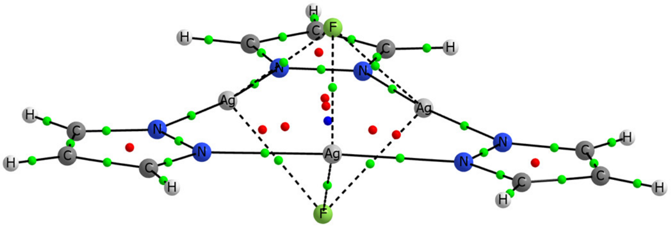	MP2= -1313.77137985 NIMAG= 0<br>X<br>X,1,r21<br>X,1,r21,2,120.<br>X,1,r21,2,120.,3,180.,0<br>N,2,r52,1,90.,3,0.,0<br>N,2,r52,1,90.,4,0.,0<br>N,3,r52,1,90.,2,0.,0<br>N,3,r52,1,90.,4,0.,0<br>N,4,r52,1,90.,3,0.,0

	N,4,r52,1,90.,2,0.,0<br>C,5,r115,2,a1152,1,180.,0<br>C,6,r115,2,a1152,1,180.,0<br>C,7,r115,3,a1152,1,180.,0<br>C,8,r115,3,a1152,1,180.,0<br>C,9,r115,4,a1152,1,180.,0<br>C,10,r115,4,a1152,1,180.,0<br>C,1,r171,3,120.,2,0.,0<br>C,1,r171,4,120.,3,0.,0<br>C,1,r171,2,120.,4,0.,0<br>H,11,r2011,5,a20115,2,180.,0<br>H,12,r2011,6,a20115,2,180.,0<br>H,13,r2011,7,a20115,3,180.,0<br>H,14,r2011,8,a20115,3,180.,0<br>H,15,r2011,9,a20115,4,180.,0<br>H,16,r2011,10,a20115,4,180.,0<br>H,1,r261,3,120.,2,0.,0<br>H,1,r261,4,120.,3,0.,0<br>H,1,r261,2,120.,4,0.,0<br>Ag,1,r291,3,60.,2,0.,0<br>Ag,1,r291,4,60.,3,0.,0<br>Ag,1,r291,2,60.,4,0.,0<br>F,1,rcl,2,90.,3,90.,0<br>F,1,rcl,2,90.,3,-90.,0<br><br>r21=2.87160594<br>r52=0.68467026<br>r115=1.36549193<br>r171=5.04193545<br>r2011=1.09128504<br>r261=6.13395387<br>r291=1.85382051<br>a1152=108.16910165<br>a20115=119.80782174<br>rcl=1.58245635

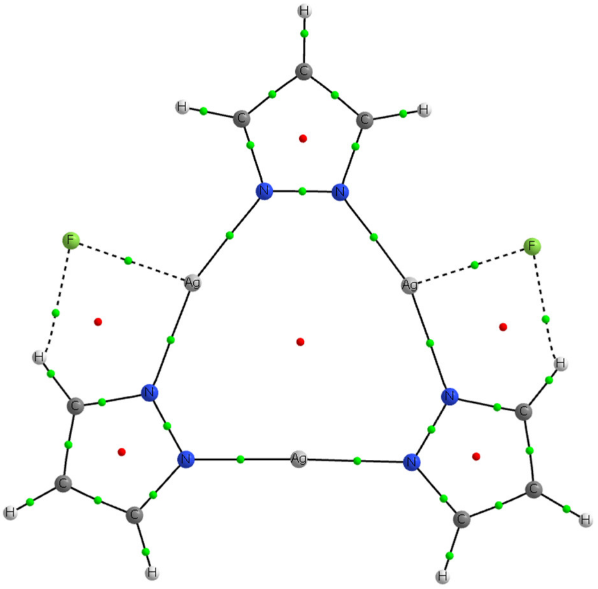	MP2= -1313.78379123 NIMAG= 0<br>N,0.6340192821,0.,2.7693317219<br>N,-0.7337615116,0.,2.7813064002<br>N,2.7849830503,0.,-0.816363517<br>N,2.0994830696,0.,-2.0036843122<br>N,-2.0813019816,0.,-1.9337426657<br>N,-2.7755627541,0.,-0.7551970908<br>C,1.0799539837,0.,4.0569536436<br>C,-1.155372962,0.,4.0851811306<br>C,4.123013411,0.,-1.0936246373<br>C,3.0086134236,0.,-3.0238220354<br>C,-2.9734479254,0.,-2.9637444066<br>C,-4.1155571191,0.,-1.0420082294<br>C,-0.0285365756,0.,4.9322596728<br>C,4.3150572906,0.,-2.4912994883<br>C,-4.2857304625,0.,-2.4414164371<br>H,2.1655580465,0.,4.1976493486<br>H,-2.2219152569,0.,4.3163401187<br>H,4.8325014489,0.,-0.2642845155<br>H,2.6451278287,0.,-4.0529267608<br>H,-2.5524919488,0.,-3.974252956<br>H,-4.8490178227,0.,-0.2339350018<br>H,-0.0210822566,0.,6.0232208091<br>H,5.2600272911,0.,-3.0368781725<br>H,-5.2268033616,0.,-2.9933526347<br>Ag,1.9808205748,0.,1.1228144764<br>Ag,0.0180244271,0.,-2.2768481764<br>Ag,-1.7284735485,0.,0.9979346685<br>F,3.7186453294,0.,2.6696607397<br>F,-0.4526713554,0.,-4.5552716928

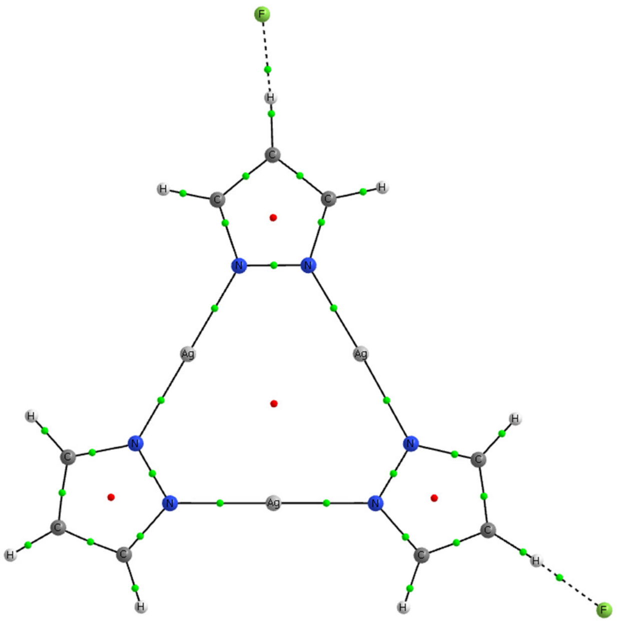	MP2= -1313.76077782 NIMAG= 0<br>N,0.6834719946,0.,2.706992012<br>N,-0.6834719946,0.,2.706992012<br>N,2.7117133194,0.,-0.8226119284<br>N,2.0369437493,0.,-2.0167850507<br>N,-2.0369437493,0.,-2.0167850507<br>N,-2.7117133194,0.,-0.8226119284<br>C,1.1125748895,0.,4.0036159513<br>C,-1.1125748895,0.,4.0036159513<br>C,4.0581026893,0.,-1.0997043486<br>C,2.9770234624,0.,-3.0187282969<br>C,-2.9770234624,0.,-3.0187282969<br>C,-4.0581026893,0.,-1.0997043486<br>C,0.,0.,4.8652668149<br>C,4.2836377419,0.,-2.4888538381<br>C,-4.2836377419,0.,-2.4888538381<br>H,2.1798587122,0.,4.2235837635<br>H,-2.1798587122,0.,4.2235837635<br>H,4.7739184331,0.,-0.2763690913<br>H,2.6434227894,0.,-4.0573978808<br>H,-2.6434227894,0.,-4.0573978808<br>H,-4.7739184331,0.,-0.2763690913<br>H,0.,0.,5.9545732176<br>H,5.2843267048,0.,-3.0222652324<br>H,-5.2843267048,0.,-3.0222652324<br>Ag,1.7007952942,0.,0.9270998121<br>Ag,0.,0.,-2.0434036517<br>Ag,-1.7007952942,0.,0.9270998121<br>F,6.8010654903,0.,-3.6967940582<br>F,-6.8010654903,0.,-3.6967940582

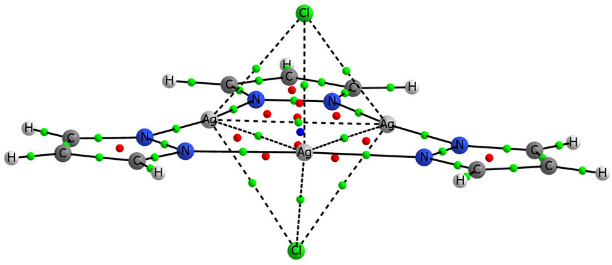	tri_pz_ag_cl_2_mp2<br>MP2= -2033.85844322 NIMAG= 0<br>X<br>X,1,r21<br>X,1,r21,2,120.<br>X,1,r21,2,120.,3,180.,0<br>N,2,r52,1,90.,3,0.,0<br>N,2,r52,1,90.,4,0.,0<br>N,3,r52,1,90.,2,0.,0<br>N,3,r52,1,90.,4,0.,0<br>N,4,r52,1,90.,3,0.,0<br>N,4,r52,1,90.,2,0.,0

	C,5,r115,2,a1152,1,180.,0<br>C,6,r115,2,a1152,1,180.,0<br>C,7,r115,3,a1152,1,180.,0<br>C,8,r115,3,a1152,1,180.,0<br>C,9,r115,4,a1152,1,180.,0<br>C,10,r115,4,a1152,1,180.,0<br>C,1,r171,3,120.,2,0.,0<br>C,1,r171,4,120.,3,0.,0<br>C,1,r171,2,120.,4,0.,0<br>H,11,r2011,5,a20115,2,180.,0<br>H,12,r2011,6,a20115,2,180.,0<br>H,13,r2011,7,a20115,3,180.,0<br>H,14,r2011,8,a20115,3,180.,0<br>H,15,r2011,9,a20115,4,180.,0<br>H,16,r2011,10,a20115,4,180.,0<br>H,1,r261,3,120.,2,0.,0<br>H,1,r261,4,120.,3,0.,0<br>H,1,r261,2,120.,4,0.,0<br>Ag,1,r291,3,60.,2,0.,0<br>Ag,1,r291,4,60.,3,0.,0<br>Ag,1,r291,2,60.,4,0.,0<br>Cl,1,rcl,2,90.,3,90.,0<br>Cl,1,rcl,2,90.,3,-90.,0<br><br>r21=2.8695558<br>r52=0.68373106<br>r115=1.36520889<br>r171=5.03777307<br>r2011=1.09116016<br>r261=6.12948423<br>r291=1.84527126<br>a1152=108.23116793<br>a20115=119.89868203<br>rcl=2.16522942

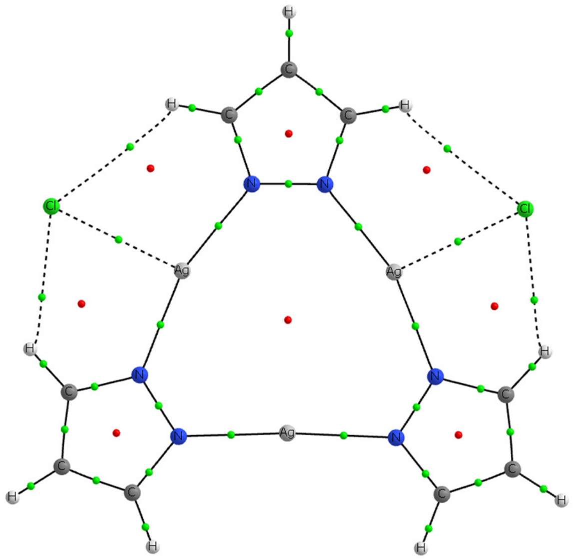	MP2= -2033.87713681 NIMAG= 0<br>N,0.6692784101,0.,2.8148164179<br>N,-0.6975983867,0.,2.7583331078<br>N,2.7525543877,0.,-0.7973134508<br>N,2.0667708971,0.,-1.9851252996<br>N,-2.1030633199,0.,-1.9870203143<br>N,-2.7375857368,0.,-0.7750286294<br>C,1.0424933976,0.,4.1261904613<br>C,-1.1870329475,0.,4.0374879226<br>C,4.0914462872,0.,-1.0735738859<br>C,2.9754654016,0.,-3.00650948<br>C,-3.0521390615,0.,-2.9659209963<br>C,-4.0900835822,0.,-0.9907432736<br>C,-0.1080451725,0.,4.9423995865<br>C,4.2793302699,0.,-2.4706724833<br>C,-4.3342661838,0.,-2.3776299291<br>H,2.1089799019,0.,4.3584665011<br>H,-2.2640682671,0.,4.2113899085<br>H,4.7986706896,0.,-0.2421162576<br>H,2.6090141746,0.,-4.0347125927<br>H,-2.7200527605,0.,-4.0056634216<br>H,-4.7792047796,0.,-0.1449543191<br>H,-0.1542858387,0.,6.0318495023<br>H,5.2240225345,0.,-3.0160908165<br>H,-5.3008778201,0.,-2.8823092954<br>Ag,1.980615552,0.,1.1509989694<br>Ag,-0.0064865712,0.,-2.2907628679<br>Ag,-1.6527433727,0.,0.9542118311<br>Cl,4.2480339112,0.,2.6778167723<br>Cl,-0.1950403959,0.,-5.0178136694
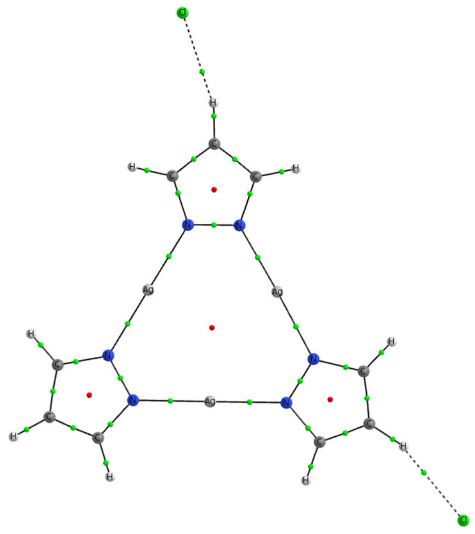	MP2= -2033.85619152 NIMAG= 0<br>N,0.6836020158,0.,2.6864675021<br>N,-0.6836020158,0.,2.6864675021<br>N,2.7130971773,0.,-0.8454242555<br>N,2.0379369488,0.,-2.0384224112<br>N,-2.0379369488,0.,-2.0384224112<br>N,-2.7130971773,0.,-0.8454242555<br>C,1.112651153,0.,3.9831782774<br>C,-1.112651153,0.,3.9831782774<br>C,4.0569240634,0.,-1.1156322219<br>C,2.9696968284,0.,-3.0444022067<br>C,-2.9696968284,0.,-3.0444022067<br>C,-4.0569240634,0.,-1.1156322219

	C,0.,0.,4.8439290569<br>C,4.2684960599,0.,-2.5039480128<br>C,-4.2684960599,0.,-2.5039480128<br>H,2.1797366064,0.,4.2037702504<br>H,-2.1797366064,0.,4.2037702504<br>H,4.7818462464,0.,-0.301991067<br>H,2.6390712975,0.,-4.0830915091<br>H,-2.6390712975,0.,-4.0830915091<br>H,-4.7818462464,0.,-0.301991067<br>H,0.,0.,5.9330474203<br>H,5.2415901892,0.,-3.0110219699<br>H,-5.2415901892,0.,-3.0110219699<br>Ag,1.7037047019,0.,0.9114268027<br>Ag,0.,0.,-2.0634322486<br>Ag,-1.7037047019,0.,0.9114268027<br>Cl,7.7418793241,0.,-3.4476812912<br>Cl,-7.7418793241,0.,-3.4476812912

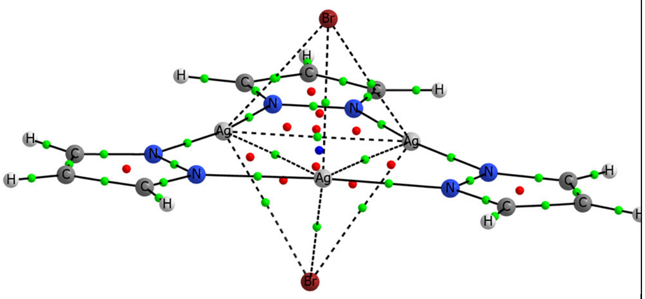	MP2= -1945.84431316 NIMAG= 0<br>N,0.6836420484,0.0000855586,2.86950194<br>83<br>N,-<br>0.6836387761,0.0000858494,2.869500323<br>N,2.8268793284,0.0000837422,-<br>0.8427016069<br>N,2.1432406546,0.0000808228,-<br>2.0268030038<br>N,-2.143241689,0.0000802372,-<br>2.0268015385<br>N,-2.8268805375,0.0000857215,-<br>0.8427015024<br>C,1.1110670304,-<br>0.0000194003,4.1659217533<br>C,-1.1110658242,-<br>0.0000165907,4.1659191363<br>C,4.1633246362,-0.0000199535,-<br>1.1207476111<br>C,3.0522610253,-0.0000185799,-<br>3.0451732505<br>C,-3.0522612772,-0.0000144392,-<br>3.0451722173<br>C,-4.1633260245,-0.000023942,-<br>1.1207472898

	C,-0.0000003046,-0.0000947432,5.0370626659<br>C,4.3622246972,-0.0000947999,-2.5185303202<br>C,-4.3622253369,-0.0000950881,-2.5185300577<br>H,2.1796010895,-0.0000356994,4.3868121971<br>H,-2.1796002296,-0.0000304713,4.3868076082<br>H,4.8888864843,-0.000034738,-0.3058135058<br>H,2.709291218,-0.0000333034,-4.080996256<br>H,-2.7092910098,-0.0000263941,-4.0809950713<br>H,-4.8888882212,-0.0000417024,-0.3058134537<br>H,-0.000001324,-0.000186619,6.1287500875<br>H,5.307654564,-0.0001851804,-3.0643725486<br>H,-5.307654891,-0.0001859255,-3.0643727697<br>Ag,1.58403495,0.0001201194,0.914543981<br>Ag,-0.0000000795,0.000109519,-1.8290868143<br>Ag,-1.5840323789,0.000122607,0.914539883<br>Br,0.0000004143,-2.3134894321,-0.0000025626<br>Br,-0.0000002371,2.3137928249,0.0000017969

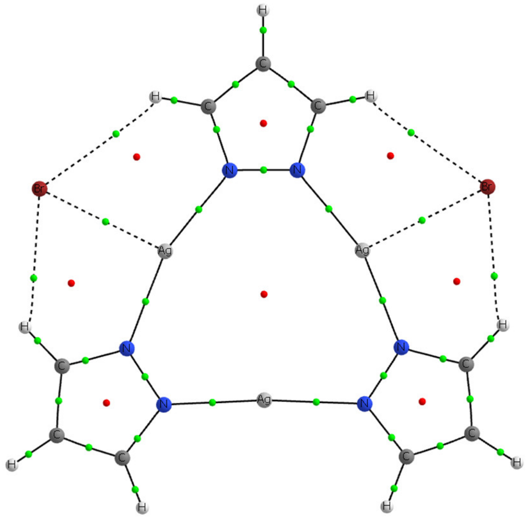

MP2= -1945.85929588 NIMAG= 0
N,0.6770060969,0.,2.8261660211
N,-0.6890223722,0.,2.7509393189
N,2.7500529682,0.,-0.7954035307
N,2.0638661479,0.,-1.9839139669
N,-2.1090285212,0.,-1.999387489
N,-2.7268945206,0.,-0.7787587814
C,1.0301882945,0.,4.1433774874
C,-1.1969830942,0.,4.0226829313
C,4.0891032452,0.,-1.0728660453
C,2.9736808727,0.,-3.0048342664
C,-3.0731760144,0.,-2.9638579774
C,-4.082237157,0.,-0.9747236982
C,-0.1315191742,0.,4.9428907954
C,4.2768110622,0.,-2.469218018
C,-4.3464285841,0.,-2.3575464518
H,2.0913615726,0.,4.3966365256
H,-2.2764225464,0.,4.1807053333
H,4.7985993818,0.,-0.243754698
H,2.6103974517,0.,-4.0338316182
H,-2.7619181361,0.,-4.0094905132
H,-4.7588082976,0.,-0.1189129118
H,-0.1926751989,0.,6.0314838369
H,5.2213702236,0.,-3.0145595041
H,-5.3197558247,0.,-2.8488803016
Ag,1.9929156,0.,1.1618628371
Ag,-0.0097449327,0.,-2.3068469558
Ag,-1.6338418257,0.,0.9432990179
Br,4.3727292982,0.,2.7272745535
Br,-0.1755243973,0.,-5.1505319329

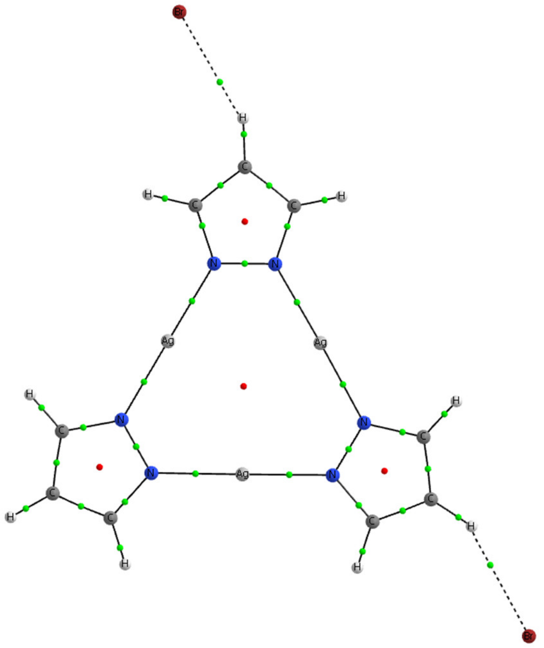	MP2= -1945.83825799 NIMAG= 0<br>N,0.6837306603,0.,2.6504223059<br>N,-0.6837306603,0.,2.6504223059<br>N,2.7169420021,0.,-0.8804843246<br>N,2.0372711729,0.,-2.071068092<br>N,-2.0372711729,0.,-2.071068092<br>N,-2.7169420021,0.,-0.8804843246<br>C,1.1126809577,0.,3.9471536898<br>C,-1.1126809577,0.,3.9471536898<br>C,4.0593644215,0.,-1.1535418219<br>C,2.9643604894,0.,-3.0809387432<br>C,-2.9643604894,0.,-3.0809387432<br>C,-4.0593644215,0.,-1.1535418219<br>C,0.,0.,4.8077176394<br>C,4.2633378613,0.,-2.5424596029<br>C,-4.2633378613,0.,-2.5424596029<br>H,2.1797534587,0.,4.1678867218<br>H,-2.1797534587,0.,4.1678867218<br>H,4.7919299349,0.,-0.3469880165<br>H,2.629407205,0.,-4.1182201216<br>H,-2.629407205,0.,-4.1182201216<br>H,-4.7919299349,0.,-0.3469880165<br>H,0.,0.,5.8967947659<br>H,5.2356565499,0.,-3.0433772421<br>H,-5.2356565499,0.,-3.0433772421<br>Ag,1.7076887209,0.,0.8780474971<br>Ag,0.,0.,-2.092108668<br>Ag,-1.7076887209,0.,0.8780474971<br>Br,8.0097158274,0.,-2.962634118<br>Br,-8.0097158274,0.,-2.962634118

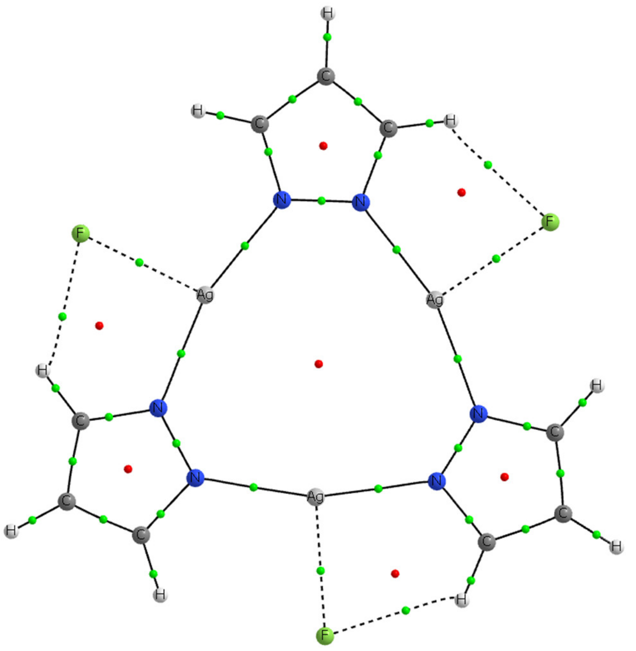

MP2= -1413.33410188 NIMAG= 0
N,2.0770234505,2.0065051895,0.
N,2.7877750217,0.8339166705,0.
N,-2.1161906449,1.9970951658,0.
N,-2.7766291359,0.7955427971,0.
N,-0.6717914492,-2.8312640559,0.
N,0.6990362856,-2.802046163,0.
C,2.9567938161,3.0509702582,0.
C,4.1193772809,1.1409895713,0.
C,-3.0475670163,2.997120365,0.
C,-4.1210150428,1.035593887,0.
C,-1.0716693873,-4.1380015281,0.
C,1.1637496209,-4.0861920796,0.
C,4.2774018335,2.5458760107,0.
C,-4.3434058262,2.4318904064,0.
C,0.0659988538,-4.9772860986,0.
H,2.5400814212,4.0618687343,0.
H,4.8424027577,0.3223866829,0.
H,-2.6997122779,4.0324017526,0.
H,-4.7881785659,0.1693542652,0.
H,-2.1420618506,-4.3550362123,0.
H,2.2475968949,-4.2304810244,0.
H,5.2114791522,3.1129020416,0.
H,-5.301392823,2.9576608309,0.
H,0.0897529614,-6.0697788229,0.
Ag,-0.0230337063,2.3036672882,0.
Ag,-1.9844364922,-1.172171348,0.
Ag,2.0068671051,-1.1321019238,0.
F,0.1654123113,4.7070373474,0.
F,-4.1612152342,-2.2089746233,0.
F,3.9925506858,-2.4994433848,0.

<table>
<tr>
<td>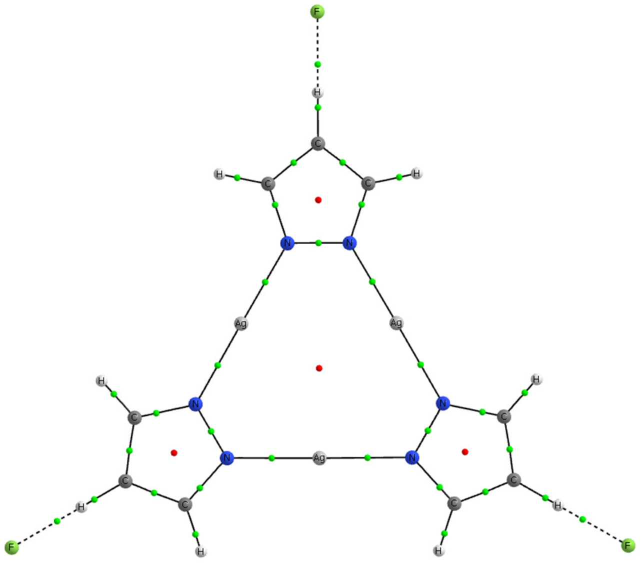
</td>
<td>MP2= -1413.35860701 NIMAG= 0
X
X,1,r21
X,1,r21,2,120.
X,1,r21,2,120.,3,180.,0
N,2,r52,1,90.,3,0.,0
N,2,r52,1,90.,4,0.,0
N,3,r52,1,90.,2,0.,0
N,3,r52,1,90.,4,0.,0
N,4,r52,1,90.,3,0.,0
N,4,r52,1,90.,2,0.,0
C,5,r115,2,a1152,1,180.,0
C,6,r115,2,a1152,1,180.,0
C,7,r115,3,a1152,1,180.,0
C,8,r115,3,a1152,1,180.,0
C,9,r115,4,a1152,1,180.,0
C,10,r115,4,a1152,1,180.,0
C,1,r171,3,120.,2,0.,0
C,1,r171,4,120.,3,0.,0
C,1,r171,2,120.,4,0.,0
H,11,r2011,5,a20115,2,180.,0
H,12,r2011,6,a20115,2,180.,0
H,13,r2011,7,a20115,3,180.,0
H,14,r2011,8,a20115,3,180.,0
H,15,r2011,9,a20115,4,180.,0
H,16,r2011,10,a20115,4,180.,0
H,1,r261,3,120.,2,0.,0
H,1,r261,4,120.,3,0.,0
H,1,r261,2,120.,4,0.,0
Ag,1,r291,3,60.,2,0.,0
Ag,1,r291,4,60.,3,0.,0
Ag,1,r291,2,60.,4,0.,0
F,1,rf,29,60.,2,180.,0
F,1,rf,30,60.,3,180.,0
F,1,rf,31,60.,4,180.,0

r21=2.74942384
r52=0.68545748
r115=1.37361623
r171=4.93672313
r2011=1.09087501
r261=6.05920163
r291=1.97479365</td>
</tr>
</table>

	a1152=107.65730918<br>a20115=119.23117844<br>rf=7.83637357

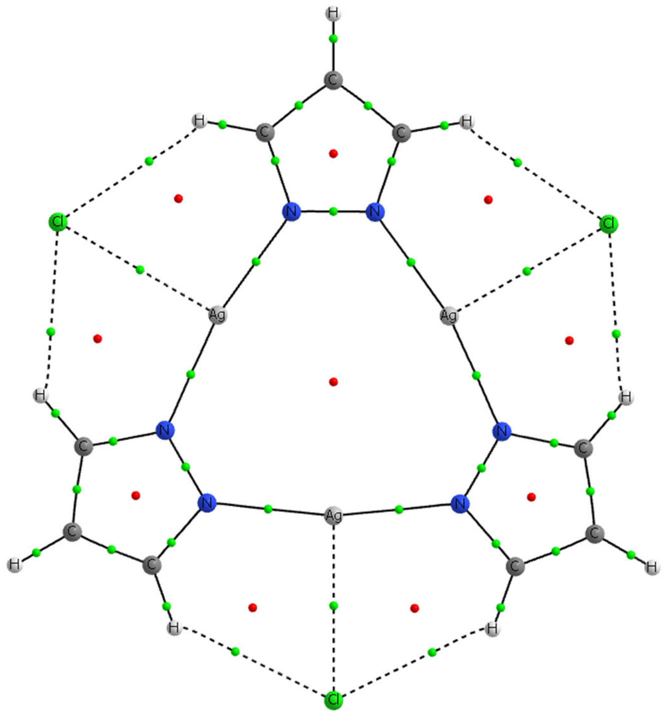	MP2= -2493.49098353 NIMAG= 0<br>X<br>X,1,r21<br>X,1,r21,2,120.<br>X,1,r21,2,120.,3,180.,0<br>N,2,r52,1,90.,3,0.,0<br>N,2,r52,1,90.,4,0.,0<br>N,3,r52,1,90.,2,0.,0<br>N,3,r52,1,90.,4,0.,0<br>N,4,r52,1,90.,3,0.,0<br>N,4,r52,1,90.,2,0.,0<br>C,5,r115,2,a1152,1,180.,0<br>C,6,r115,2,a1152,1,180.,0<br>C,7,r115,3,a1152,1,180.,0<br>C,8,r115,3,a1152,1,180.,0<br>C,9,r115,4,a1152,1,180.,0<br>C,10,r115,4,a1152,1,180.,0<br>C,1,r171,3,120.,2,0.,0<br>C,1,r171,4,120.,3,0.,0<br>C,1,r171,2,120.,4,0.,0<br>H,11,r2011,5,a20115,2,180.,0<br>H,12,r2011,6,a20115,2,180.,0<br>H,13,r2011,7,a20115,3,180.,0<br>H,14,r2011,8,a20115,3,180.,0<br>H,15,r2011,9,a20115,4,180.,0<br>H,16,r2011,10,a20115,4,180.,0<br>H,1,r261,3,120.,2,0.,0<br>H,1,r261,4,120.,3,0.,0<br>H,1,r261,2,120.,4,0.,0<br>Ag,1,r291,3,60.,2,0.,0<br>Ag,1,r291,4,60.,3,0.,0<br>Ag,1,r291,2,60.,4,0.,0<br>Cl,1,rf,3,60.,2,0.,0<br>Cl,1,rf,4,60.,3,0.,0<br>Cl,1,rf,2,60.,4,0.,0<br><br>r21=2.79397013<br>r52=0.68571669

	r115=1.36612675<br>r171=4.95001333<br>r2011=1.09202493<br>r261=6.04151947<br>r291=2.19264029<br>a1152=108.47621532<br>a20115=118.51123295<br>rf=5.21805156
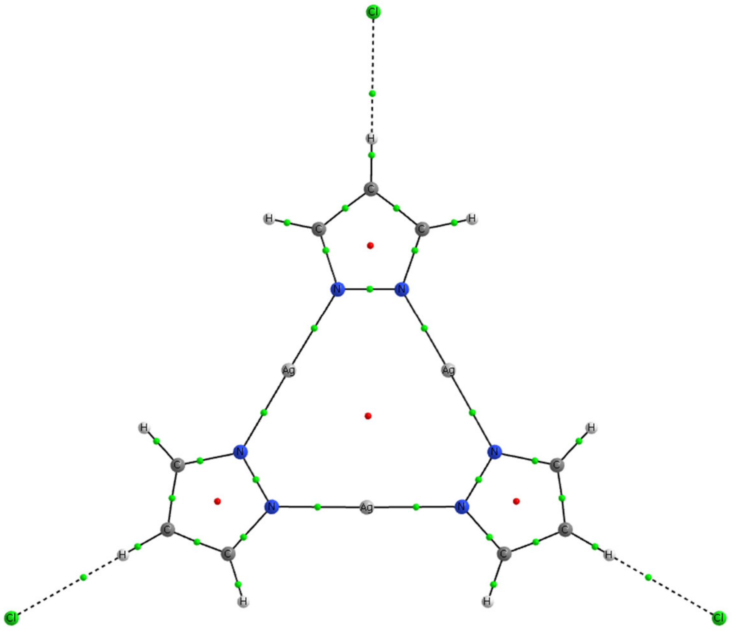	MP2= -2493.51272106 NIMAG= 0<br>N,0.6851598509,0.,2.7501859923<br>N,-0.6851598509,0.,2.7501859923<br>N,2.7243117377,0.,-0.781722259<br>N,2.0391442088,0.,-1.968445031<br>N,-2.0391442088,0.,-1.968445031<br>N,-2.7243117377,0.,-0.781722259<br>C,1.1072561745,0.,4.0540494066<br>C,-1.1072561745,0.,4.0540494066<br>C,4.0645351664,0.,-1.0681191336<br>C,2.9572600363,0.,-2.9859358616<br>C,-2.9572600363,0.,-2.9859358616<br>C,-4.0645351664,0.,-1.0681191336<br>C,0.,0.,4.9215300289<br>C,4.2621530781,0.,-2.460775003<br>C,-4.2621530781,0.,-2.460775003<br>H,2.1738111543,0.,4.2785608512<br>H,-2.1738111543,0.,4.2785608512<br>H,4.7922521737,0.,-0.2567172315<br>H,2.6184028184,0.,-4.0218514866<br>H,-2.6184028184,0.,-4.0218514866<br>H,-4.7922521737,0.,-0.2567172315<br>H,0.,0.,6.0179285632<br>H,5.2116562812,0.,-3.0089840097<br>H,-5.2116562812,0.,-3.0089840097<br>Ag,1.7123650959,0.,0.9886341926<br>Ag,0.,0.,-1.977205477

	Ag,-<br>1.7123650959,0.,0.9886341926<br>Cl,7.584478187,0.,-4.3789157976<br>Cl,-7.584478187,0.,-4.3789157976<br>Cl,0.,0.,8.7578186132

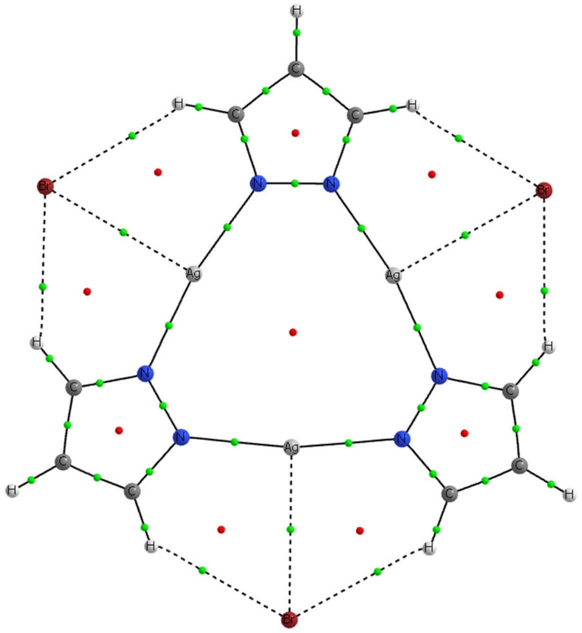	MP2= -2361.46823649 NIMAG= 0<br>X<br>X,1,r21<br>X,1,r21,2,120.<br>X,1,r21,2,120.,3,180.,0<br>N,2,r52,1,90.,3,0.,0<br>N,2,r52,1,90.,4,0.,0<br>N,3,r52,1,90.,2,0.,0<br>N,3,r52,1,90.,4,0.,0<br>N,4,r52,1,90.,3,0.,0<br>N,4,r52,1,90.,2,0.,0<br>C,5,r115,2,a1152,1,180.,0<br>C,6,r115,2,a1152,1,180.,0<br>C,7,r115,3,a1152,1,180.,0<br>C,8,r115,3,a1152,1,180.,0<br>C,9,r115,4,a1152,1,180.,0<br>C,10,r115,4,a1152,1,180.,0<br>C,1,r171,3,120.,2,0.,0<br>C,1,r171,4,120.,3,0.,0<br>C,1,r171,2,120.,4,0.,0<br>H,11,r2011,5,a20115,2,180.,0<br>H,12,r2011,6,a20115,2,180.,0<br>H,13,r2011,7,a20115,3,180.,0<br>H,14,r2011,8,a20115,3,180.,0<br>H,15,r2011,9,a20115,4,180.,0<br>H,16,r2011,10,a20115,4,180.,0<br>H,1,r261,3,120.,2,0.,0<br>H,1,r261,4,120.,3,0.,0<br>H,1,r261,2,120.,4,0.,0<br>Ag,1,r291,3,60.,2,0.,0<br>Ag,1,r291,4,60.,3,0.,0<br>Ag,1,r291,2,60.,4,0.,0<br>Br,1,rf,3,60.,2,0.,0<br>Br,1,rf,4,60.,3,0.,0<br>Br,1,rf,2,60.,4,0.,0<br><br>r21=2.78777393

	r52=0.68572162<br>r115=1.36626381<br>r171=4.94273653<br>r2011=1.09169169<br>r261=6.03388486<br>r291=2.16154697<br>a1152=108.47041342<br>a20115=118.71279244<br>rf=5.3999374
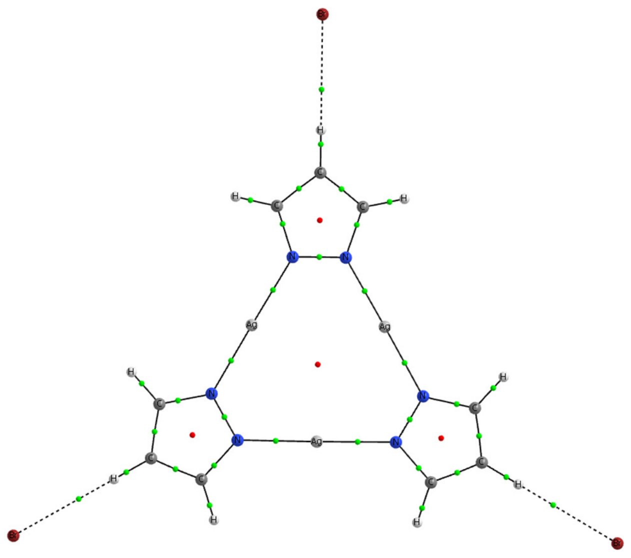	MP2= -2361.48741177 NIMAG= 0<br>N,0.6850495639,0.,2.7503041832<br>N,-0.6850495639,0.,2.7503041832<br>N,2.7243518812,0.,-0.7818772151<br>N,2.0393017978,0.,-1.9684153026<br>N,-2.0393017978,0.,-1.9684153026<br>N,-2.7243518812,0.,-0.7818772151<br>C,1.1079138977,0.,4.0534702125<br>C,-1.1079138977,0.,4.0534702125<br>C,4.0643589206,0.,-1.0672503843<br>C,2.9564411232,0.,-2.9862122949<br>C,-2.9564411232,0.,-2.9862122949<br>C,-4.0643589206,0.,-1.0672503843<br>C,0.,0.,4.9196369031<br>C,4.26052172,0.,-2.459816028<br>C,-4.26052172,0.,-2.459816028<br>H,2.1742791756,0.,4.2784256277<br>H,-2.1742791756,0.,4.2784256277<br>H,4.7923598312,0.,-0.2562296466<br>H,2.6180733793,0.,-4.0221887626<br>H,-2.6180733793,0.,-4.0221887626<br>H,-4.7923598312,0.,-0.2562296466<br>H,0.,0.,6.0143474095<br>H,5.2085675349,0.,-3.0071732989<br>H,-5.2085675349,0.,-3.0071732989<br>Ag,1.7127257571,0.,0.9888475796<br>Ag,0.,0.,-1.9776712736<br>Ag,-1.7127257571,0.,0.9888475796<br>Br,7.779709616,0.,-4.4916761175<br>Br,-7.779709616,0.,-4.4916761175<br>Br,0.,0.,8.9832708121

Table S3. Molecular graph, electronic energy and geometry of the $(Pz\text{-}Cu)_3{:}X^{-}_{n}$ complexes.

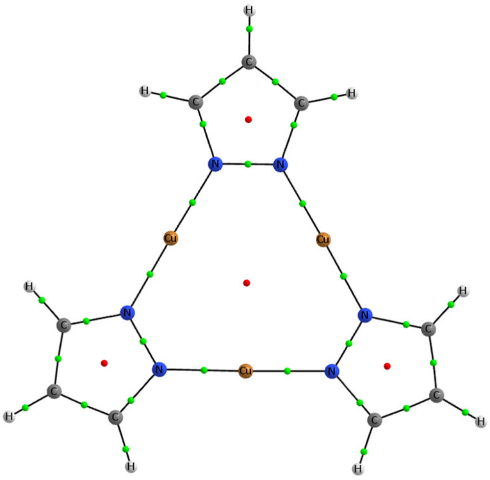	MP2= -1265.30943599 NIMAG= 0<br>X<br>X,1,r21<br>X,1,r21,2,120.<br>X,1,r21,2,120.,3,180.,0<br>N,2,r52,1,90.,3,0.,0<br>N,2,r52,1,90.,4,0.,0<br>N,3,r52,1,90.,2,0.,0<br>N,3,r52,1,90.,4,0.,0<br>N,4,r52,1,90.,3,0.,0<br>N,4,r52,1,90.,2,0.,0<br>C,5,r115,2,a1152,1,180.,0<br>C,6,r115,2,a1152,1,180.,0<br>C,7,r115,3,a1152,1,180.,0<br>C,8,r115,3,a1152,1,180.,0<br>C,9,r115,4,a1152,1,180.,0<br>C,10,r115,4,a1152,1,180.,0<br>C,1,r171,3,120.,2,0.,0<br>C,1,r171,4,120.,3,0.,0<br>C,1,r171,2,120.,4,0.,0<br>H,11,r2011,5,a20115,2,180.,0<br>H,12,r2011,6,a20115,2,180.,0<br>H,13,r2011,7,a20115,3,180.,0<br>H,14,r2011,8,a20115,3,180.,0<br>H,15,r2011,9,a20115,4,180.,0<br>H,16,r2011,10,a20115,4,180.,0<br>H,1,r261,3,120.,2,0.,0<br>H,1,r261,4,120.,3,0.,0<br>H,1,r261,2,120.,4,0.,0<br>Cu,1,r291,3,60.,2,0.,0<br>Cu,1,r291,4,60.,3,0.,0<br>Cu,1,r291,2,60.,4,0.,0<br><br>r21=2.49391542<br>r52=0.68517656<br>r115=1.36634178<br>r171=4.64884563<br>r2011=1.08892227<br>r261=5.73710754<br>r291=1.85459423<br>a1152=108.21748163<br>a20115=120.11537875

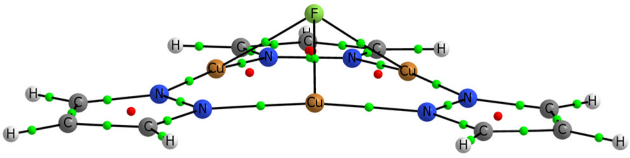	MP2= -1365.05306570 NIMAG= 0<br>N,0.686985083,2.5188796847,0.0162671161<br>N,-0.6870979556,2.5189761251,0.0162613917<br>N,2.5249251179,-0.6644761179,0.0162611276<br>N,1.8379307704,-1.8544851676,0.0162503429<br>N,-1.8379506548,-1.8544124181,0.016244903<br>N,-2.52503866,-0.6644688411,0.0162354006<br>C,1.1112828867,3.8119968856,0.1235501368<br>C,-1.1111932548,3.8121479414,0.1235451682<br>C,3.8569335789,-0.943640824,0.1234868565<br>C,2.745763744,-2.8683944107,0.1234699896<br>C,-2.7457029753,-2.8683995782,0.1234366781<br>C,-3.8570335117,-0.9437281407,0.1234249828<br>C,0.0001070475,4.6736328286,0.1879851395<br>C,4.0474872087,-2.336767153,0.1880844424<br>C,-4.0474662678,-2.3368657435,0.1881454783<br>H,2.1778568194,4.0302603416,0.1494872982<br>H,-2.1777389769,4.0305464578,0.1494779514<br>H,4.5792678287,-0.1291147408,0.1493971196<br>H,2.4015980576,-3.9012402285,0.1493621317<br>H,-2.4014471189,-3.901212967,0.1493089606<br>H,-4.5794390187,-0.1292627477,0.1492876056<br>H,0.0001809541,5.7601237179,0.2705724078<br>H,4.9884313658,-2.8799707239,0.2707702184

	H,-4.9883612942,-2.8801457192,0.2708904207<br>Cu,1.5696330879,0.906253833,-0.102368143<br>Cu,-0.0000129908,-1.8125702477,-0.1024046546<br>Cu,-1.5698380526,0.9063165117,-0.1023856193<br>F,-0.0000628182,0.0000214356,-1.2313911312
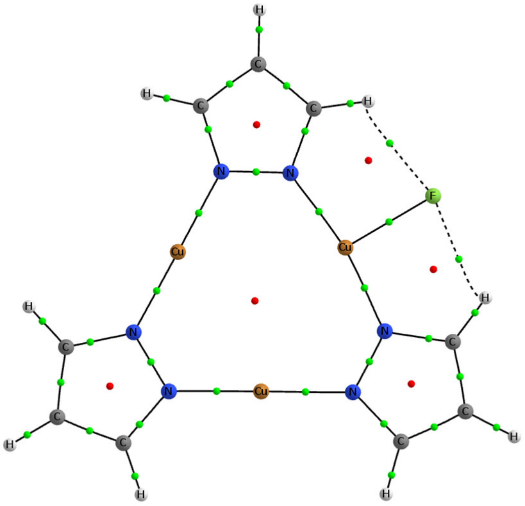	MP2= -1365.04081154 NIMAG= 0<br>N,-0.6843596385,0.,-2.6945780783<br>N,0.6843596385,0.,-2.6945780783<br>N,-2.5563172697,0.,0.4248884925<br>N,-1.8303680365,0.,1.586102275<br>N,1.8303680365,0.,1.586102275<br>N,2.5563172697,0.,0.4248884925<br>C,-1.1120075647,0.,-3.9920762603<br>C,1.1120075647,0.,-3.9920762603<br>C,-3.8877630448,0.,0.7433775147<br>C,-2.7008930959,0.,2.6353328471<br>C,2.7008930959,0.,2.6353328471<br>C,3.8877630448,0.,0.7433775147<br>C,0.,0.,-4.8525783728<br>C,-4.0213397566,0.,2.1439512732<br>C,4.0213397566,0.,2.1439512732<br>H,-2.1783817174,0.,-4.2126404155<br>H,2.1783817174,0.,-4.2126404155<br>H,-4.6403662067,0.,-0.0451031743<br>H,-2.2741711033,0.,3.6389095102<br>H,2.2741711033,0.,3.6389095102<br>H,4.6403662067,0.,-0.0451031743<br>H,0.,0.,-5.9415104408<br>H,-4.9466073638,0.,2.7193073255<br>H,4.9466073638,0.,2.7193073255<br>Cu,-1.6159896501,0.,-1.1266289386<br>Cu,0.,0.,1.763661584<br>Cu,1.6159896501,0.,-1.1266289386<br>F,0.,0.,3.7794949636

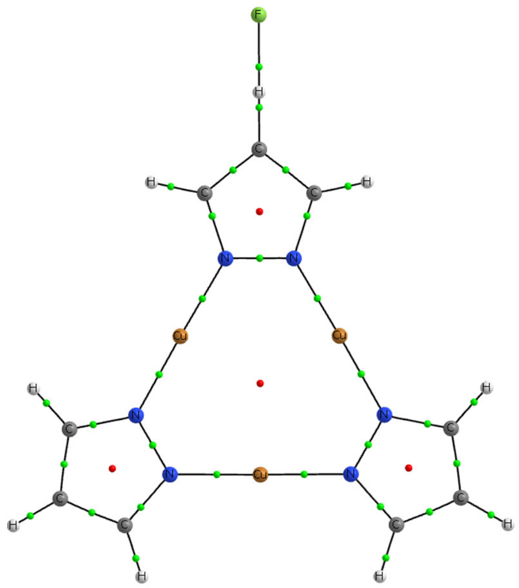	MP2= -1365.00462497 NIMAG= 0<br>N,-0.6875858922,0.,-10.8121894917<br>N,0.6875858922,0.,-10.8121894917<br>N,-2.4978420479,0.,-7.6673964034<br>N,-1.8181290769,0.,-6.4782832058<br>N,1.8181290769,0.,-6.4782832058<br>N,2.4978420479,0.,-7.6673964034<br>C,-1.0999707452,0.,-12.1237625737<br>C,1.0999707452,0.,-12.1237625737<br>C,-3.834747142,0.,-7.392550068<br>C,-2.73124605,0.,-5.4604908244<br>C,2.73124605,0.,-5.4604908244<br>C,3.834747142,0.,-7.392550068<br>C,0.,0.,-13.0015554681<br>C,-4.0279779934,0.,-5.9997283154<br>C,4.0279779934,0.,-5.9997283154<br>H,-2.1678655477,0.,-12.3450011184<br>H,2.1678655477,0.,-12.3450011184<br>H,-4.5570298688,0.,-8.2074944785<br>H,-2.3944357188,0.,-4.4248601848<br>H,2.3944357188,0.,-4.4248601848<br>H,4.5570298688,0.,-8.2074944785<br>H,0.,0.,-14.1523338568<br>H,-4.9731067249,0.,-5.459581118<br>H,4.9731067249,0.,-5.459581118<br>Cu,-1.6007735225,0.,-9.2547657289<br>Cu,0.,0.,-6.4724583933<br>Cu,1.6007735225,0.,-9.2547657289<br>F,0.,0.,-15.7033644919

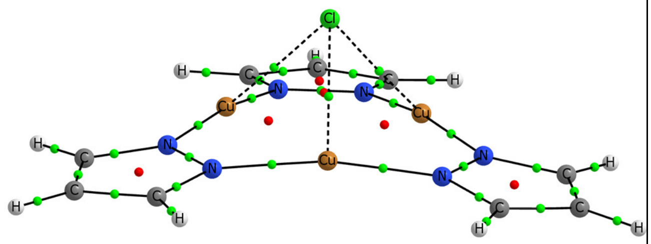	MP2= -1725.07834839 NIMAG= 0<br>N,0.6855128138,2.5150704687,-<br>0.0163992513<br>N,-0.6855251099,2.5150421796,-<br>0.0164166807<br>N,2.5209067205,-0.6638627454,-<br>0.0164221605<br>N,1.8353617657,-1.851192956,-<br>0.0163991263<br>N,-1.8353481588,-1.8511667539,-<br>0.0164009983<br>N,-2.5208854105,-0.6638278683,-<br>0.0164350464

	C,1.1117090782,3.799213522,0.1655606944<br>C,-<br>1.1117558667,3.7991800173,0.1654977579<br>C,3.8461109931,-<br>0.9368466984,0.165478738<br>C,2.7343339116,-<br>2.8623957084,0.1655231827<br>C,-2.7343272514,-<br>2.8623624191,0.1655501789<br>C,-3.8460869076,-<br>0.9368016751,0.1654884751<br>C,-<br>0.0000372457,4.6552260663,0.2788529366<br>C,4.0315592536,-<br>2.3276526081,0.2788084276<br>C,-4.0315437151,-<br>2.3276052234,0.2788459422<br>H,2.1783438726,4.0167363529,0.20146320<br>41<br>H,-<br>2.1783967966,4.0166790739,0.2013399501<br>H,4.5678178658,-<br>0.1218818096,0.201337721<br>H,2.3893559374,-<br>3.8948754762,0.2014261644<br>H,-2.3893641947,-<br>3.8948477201,0.2014556463<br>H,-4.5677857165,-<br>0.1218297369,0.2013292212<br>H,-<br>0.0000539965,5.7357110181,0.4193819536<br>H,4.9672759559,-2.8679159516,0.41932989<br>H,-4.967263956,-<br>2.8678596075,0.4193790553<br>Cu,1.5573106226,0.8990846545,-<br>0.2020603201<br>Cu,0.0000097313,-1.7981096418,-<br>0.2020478598<br>Cu,-1.5572738174,0.8990967436,-<br>0.2021172133<br>Cl,0.0000396216,-0.0000055038,-<br>2.0186967624

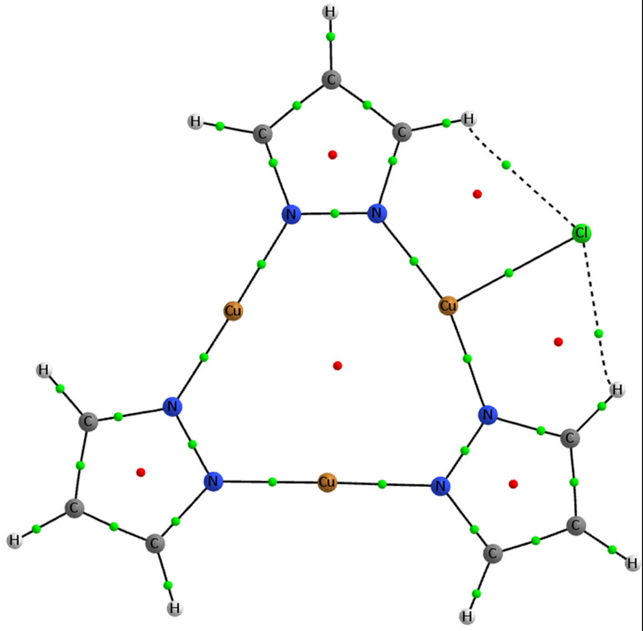	MP2= -1725.07480464 NIMAG= 0<br>N,-0.6840256023,0.,-2.7052222836<br>N,0.6840256023,0.,-2.7052222836<br>N,-2.4853905617,0.,0.4457624952<br>N,-1.8438486766,0.,1.6587192415<br>N,1.8438486766,0.,1.6587192415<br>N,2.4853905617,0.,0.4457624952<br>C,-1.1129627489,0.,-4.0017992679<br>C,1.1129627489,0.,-4.0017992679<br>C,-3.8381517048,0.,0.6615803732<br>C,-2.8003285729,0.,2.6331216235<br>C,2.8003285729,0.,2.6331216235<br>C,3.8381517048,0.,0.6615803732<br>C,0.,0.,-4.861249712<br>C,-4.0790673725,0.,2.0450727069<br>C,4.0790673725,0.,2.0450727069<br>H,-2.1791978152,0.,-4.2229894623<br>H,2.1791978152,0.,-4.2229894623<br>H,-4.527112547,0.,-0.1831869739<br>H,-2.4776374526,0.,3.6735267945<br>H,2.4776374526,0.,3.6735267945<br>H,4.527112547,0.,-0.1831869739<br>H,0.,0.,-5.9500606632<br>H,-5.0436216973,0.,2.5513404874<br>H,5.0436216973,0.,2.5513404874<br>Cu,-1.5688183432,0.,-1.1140439207<br>Cu,0.,0.,1.9442950537<br>Cu,1.5688183432,0.,-1.1140439207<br>Cl,0.,0.,4.3740041705

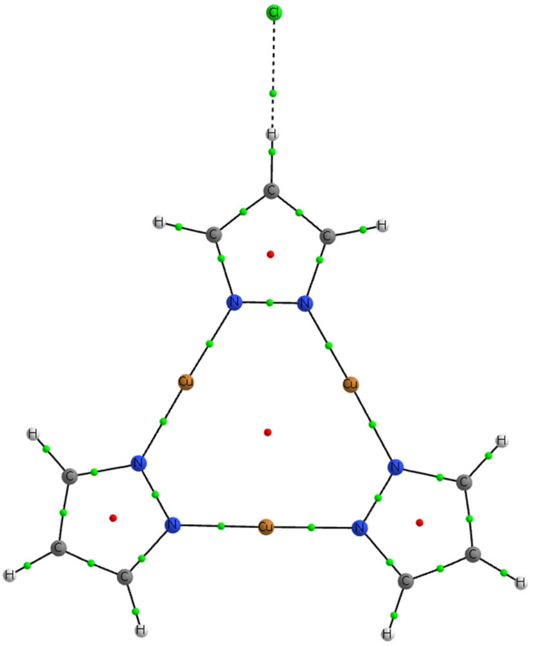

MP2= -1725.04771008 NIMAG= 0
N,-0.6870325399,0.,-10.790425632
N,0.6870325399,0.,-10.790425632
N,-2.4981865692,0.,-7.6440384859
N,-1.8178503724,0.,-6.4551119499
N,1.8178503724,0.,-6.4551119499
N,2.4981865692,0.,-7.6440384859
C,-1.1060969794,0.,-12.0960771275
C,1.1060969794,0.,-12.0960771275
C,-3.8353234421,0.,-7.3692525211
C,-2.7309738311,0.,-5.4377094503
C,2.7309738311,0.,-5.4377094503
C,3.8353234421,0.,-7.3692525211
C,0.,0.,-12.9609294259
C,-4.0277667659,0.,-5.9767406202
C,4.0277667659,0.,-5.9767406202
H,-2.1713200717,0.,-12.3246215867
H,2.1713200717,0.,-12.3246215867
H,-4.55813137,0.,-8.1837287593
H,-2.394365026,0.,-4.4020370526
H,2.394365026,0.,-4.4020370526
H,4.55813137,0.,-8.1837287593
H,0.,0.,-14.0619893265
H,-4.9726854107,0.,-5.4364145387
H,4.9726854107,0.,-5.4364145387
Cu,-1.6020043835,0.,-9.2302271682
Cu,0.,0.,-6.4470263381
Cu,1.6020043835,0.,-9.2302271682
Cl,0.,0.,-16.4192017066

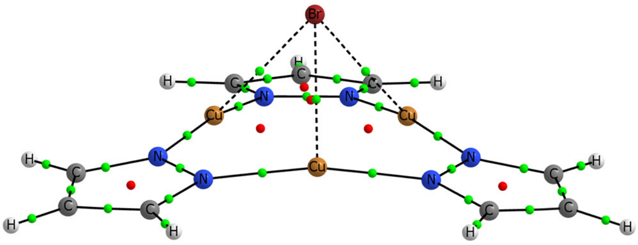

MP2= -1681.06662494 NIMAG= 0
N,0.6850400295,-0.1101182553,2.5133088822
N,-0.6850889835,-0.1101440945,2.5133342746
N,2.5191687567,-0.1101320505,-0.6633909732
N,1.8341193884,-0.1101203042,-1.8499536979
N,-1.8341003149,-0.1100912801,-1.8499527345
N,-2.5191546542,-0.1101125597,-0.6633773526

	C,1.1118605567,0.0833335596,3.795628152<br>4<br>C,-<br>1.1118766369,0.0832705109,3.7956700094<br>C,3.8430990349,0.0833215868,-<br>0.9349112672<br>C,2.7312583983,0.0832412541,-<br>2.8607411969<br>C,-2.7312369306,0.0833161254,-<br>2.8607314987<br>C,-3.8430853065,0.0833454321,-<br>0.934907023<br>C,0.0000043666,0.2041648035,4.650464128<br>4<br>C,4.0274643469,0.2042266979,-<br>2.3252220902<br>C,-4.0274559987,0.204198838,-<br>2.3252204202<br>H,2.178529695,0.1196123206,4.013000289<br>H,-<br>2.1785404847,0.1194910432,4.0130794414<br>H,4.5646856711,0.1195834832,-<br>0.1198360288<br>H,2.3862089605,0.1194295479,-<br>3.8932048337<br>H,-2.3861808554,0.1195473075,-<br>3.8931917303<br>H,-4.5646773709,0.1196012414,-<br>0.1198360633<br>H,0.0000203977,0.3532915075,5.729790142<br>3<br>H,4.9621867085,0.3533939083,-<br>2.8648756497<br>H,-4.9621852111,0.3533236344,-<br>2.8648739979<br>Cu,1.5562299583,-<br>0.3060171617,0.89848021<br>Cu,0.0000147106,-0.3059815271,-<br>1.7970269509<br>Cu,-1.5562929714,-<br>0.306042057,0.898516872<br>Br,-0.000015261,-2.3109335117,-<br>0.0000188923

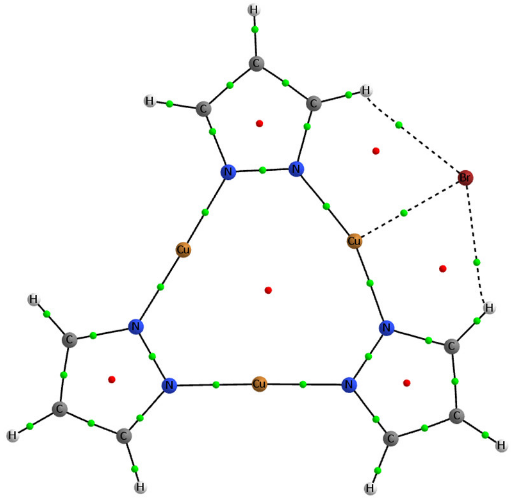	MP2= -1681.06247072 NIMAG= 0<br>N,-0.6839129762,0.,-2.7091083677<br>N,0.6839129762,0.,-2.7091083677<br>N,-2.4697199599,0.,0.4487525566<br>N,-1.8459707228,0.,1.671739356<br>N,1.8459707228,0.,1.671739356<br>N,2.4697199599,0.,0.4487525566<br>C,-1.113201974,0.,-4.0054672217<br>C,1.113201974,0.,-4.0054672217<br>C,-3.8257128408,0.,0.6438020175<br>C,-2.8190847386,0.,2.6300074155<br>C,2.8190847386,0.,2.6300074155<br>C,3.8257128408,0.,0.6438020175<br>C,0.,0.,-4.86466821<br>C,-4.0883477706,0.,2.0227594208<br>C,4.0883477706,0.,2.0227594208<br>H,-2.1794123351,0.,-4.226719722<br>H,2.1794123351,0.,-4.226719722<br>H,-4.5011673301,0.,-0.2118372021<br>H,-2.5185260383,0.,3.6766480173<br>H,2.5185260383,0.,3.6766480173<br>H,4.5011673301,0.,-0.2118372021<br>H,0.,0.,-5.9534556105<br>H,-5.0600874531,0.,2.5148664302<br>H,5.0600874531,0.,2.5148664302<br>Cu,-1.5572171373,0.,-1.1123627598<br>Cu,0.,0.,1.9740946813<br>Cu,1.5572171373,0.,-1.1123627598<br>Br,0.,0.,4.5486217363

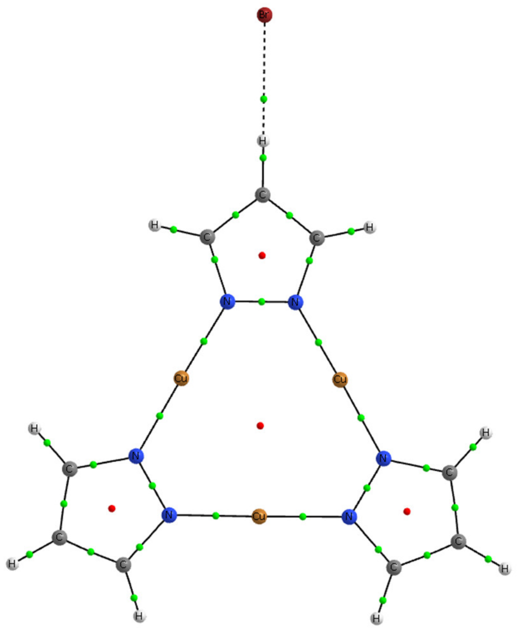	MP2= -1681.03783540 NIMAG= 0<br>N,-0.6868875416,0.,-10.7843400592<br>N,0.6868875416,0.,-10.7843400592<br>N,-2.4986172386,0.,-7.6378798611<br>N,-1.8179093903,0.,-6.4491386349<br>N,1.8179093903,0.,-6.4491386349<br>N,2.4986172386,0.,-7.6378798611<br>C,-1.1068506079,0.,-12.0892274237<br>C,1.1068506079,0.,-12.0892274237<br>C,-3.835790008,0.,-7.3628324554<br>C,-2.7308856168,0.,-5.4316056356<br>C,2.7308856168,0.,-5.4316056356<br>C,3.835790008,0.,-7.3628324554<br>C,0.,0.,-12.9525787217<br>C,-4.0277723418,0.,-5.97036245<br>C,4.0277723418,0.,-5.97036245<br>H,-2.1718351127,0.,-12.3184215305<br>H,2.1718351127,0.,-12.3184215305<br>H,-4.5588709084,0.,-8.1770696907<br>H,-2.3941291424,0.,-4.3959897279<br>H,2.3941291424,0.,-4.3959897279<br>H,4.5588709084,0.,-8.1770696907<br>H,0.,0.,-14.0503572796<br>H,-4.9725520185,0.,-5.4298067155<br>H,4.9725520185,0.,-5.4298067155<br>Cu,-1.6026166437,0.,-9.223906773<br>Cu,0.,0.,-6.4404417938<br>Cu,1.6026166437,0.,-9.223906773<br>Br,0.,0.,-16.5973768637

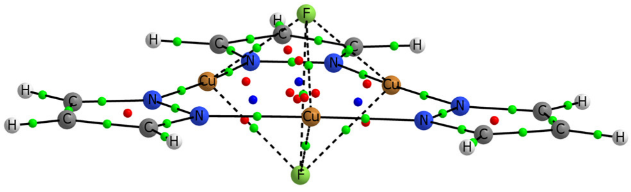	MP2= -1464.65453414 NIMAG= 0<br>X<br>X,1,r21<br>X,1,r21,2,120.<br>X,1,r21,2,120.,3,180.,0<br>N,2,r52,1,90.,3,0.,0<br>N,2,r52,1,90.,4,0.,0<br>N,3,r52,1,90.,2,0.,0<br>N,3,r52,1,90.,4,0.,0<br>N,4,r52,1,90.,3,0.,0<br>N,4,r52,1,90.,2,0.,0<br>C,5,r115,2,a1152,1,180.,0<br>C,6,r115,2,a1152,1,180.,0

	C,7,r115,3,a1152,1,180.,0<br>C,8,r115,3,a1152,1,180.,0<br>C,9,r115,4,a1152,1,180.,0<br>C,10,r115,4,a1152,1,180.,0<br>C,1,r171,3,120.,2,0.,0<br>C,1,r171,4,120.,3,0.,0<br>C,1,r171,2,120.,4,0.,0<br>H,11,r2011,5,a20115,2,180.,0<br>H,12,r2011,6,a20115,2,180.,0<br>H,13,r2011,7,a20115,3,180.,0<br>H,14,r2011,8,a20115,3,180.,0<br>H,15,r2011,9,a20115,4,180.,0<br>H,16,r2011,10,a20115,4,180.,0<br>H,1,r261,3,120.,2,0.,0<br>H,1,r261,4,120.,3,0.,0<br>H,1,r261,2,120.,4,0.,0<br>Cu,1,r291,3,60.,2,0.,0<br>Cu,1,r291,4,60.,3,0.,0<br>Cu,1,r291,2,60.,4,0.,0<br>F,1,rcl,2,90.,3,90.,0<br>F,1,rcl,2,90.,3,-90.,0<br>r21=2.53606214<br>r52=0.68717892<br>r115=1.36470525<br>r171=4.70335136<br>r2011=1.08937051<br>r261=5.79505867<br>r291=1.75190017<br>a1152=108.11361618<br>a20115=119.54685991<br>rcl=1.3487393

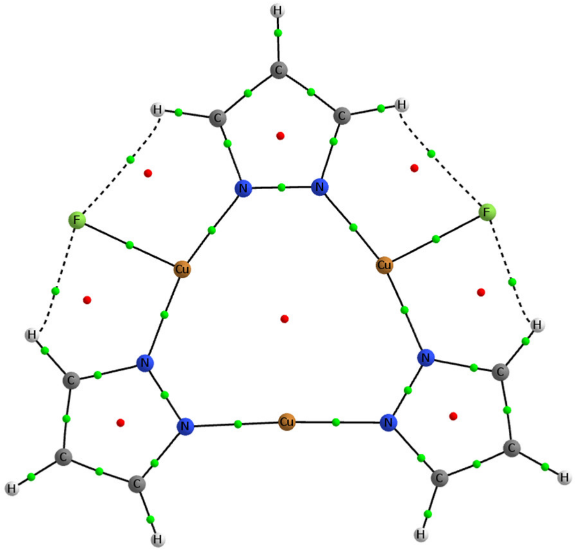

MP2= -1464.67268461 NIMAG= 0
N,0.6867279058,0.,2.5179912093
N,-0.6825049132,0.,2.500369
N,2.5117690541,0.,-0.6574584142
N,1.8252602157,0.,-1.8465266022
N,-1.8372804008,0.,-1.8537194166
N,-2.5066355295,0.,-0.6591179071
C,1.1006344469,0.,3.8164786151
C,-1.1327118858,0.,3.7944577166
C,3.8504478917,0.,-0.9330566956
C,2.7332747475,0.,-2.8680573423
C,-2.7548502102,0.,-2.861416699
C,-3.852452719,0.,-0.9162715902
C,-0.0257314844,0.,4.6663883114
C,4.037921737,0.,-2.3312952018
C,-4.0540765637,0.,-2.3109100367
H,2.1783254272,0.,3.9893293367
H,-2.2035044489,0.,4.0008596095
H,4.5434671965,0.,-0.0905160651
H,2.3501228101,0.,-3.8894999809
H,-2.365697836,0.,-3.8811498261
H,-4.5665982832,0.,-0.0921389748
H,-0.0421725102,0.,5.7570279473
H,4.9831465557,0.,-2.8770210052
H,-5.0068187077,0.,-2.8419915086
Cu,1.8143024254,0.,1.0509451009
Cu,-0.0029939426,0.,-2.096704541
Cu,-1.5507032994,0.,0.8952989673
F,3.5592554765,0.,2.2003778423
F,-0.1259553711,0.,-4.1825945824

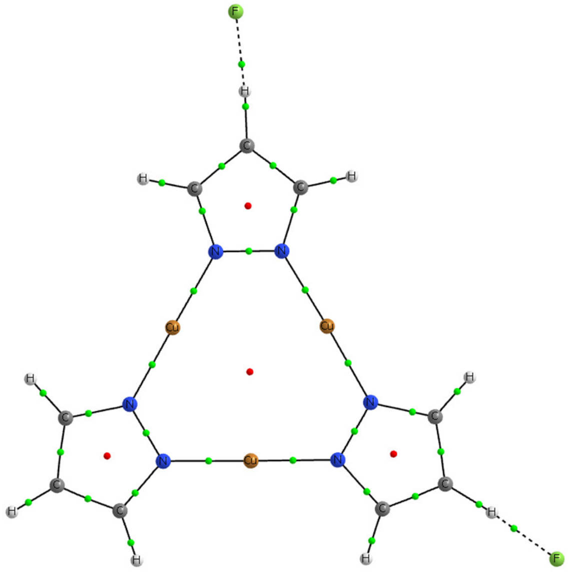	MP2= -1464.64906893 NIMAG= 0<br>N,0.6844123785,0.,2.4433849046<br>N,-0.6844123785,0.,2.4433849046<br>N,2.5002460766,0.,-0.6997477987<br>N,1.818912451,0.,-1.8931441253<br>N,-1.818912451,0.,-1.8931441253<br>N,-2.5002460766,0.,-0.6997477987<br>C,1.1127954752,0.,3.7403016669<br>C,-1.1127954752,0.,3.7403016669<br>C,3.8448249593,0.,-0.9850728636<br>C,2.7548468699,0.,-2.8983373141<br>C,-2.7548468699,0.,-2.8983373141<br>C,-3.8448249593,0.,-0.9850728636<br>C,0.,0.,4.6002711458<br>C,4.0632156604,0.,-2.3743463307<br>C,-4.0632156604,0.,-2.3743463307<br>H,2.1794343494,0.,3.9601717331<br>H,-2.1794343494,0.,3.9601717331<br>H,4.565711037,0.,-0.1669647838<br>H,2.4175714148,0.,-3.9352140873<br>H,-2.4175714148,0.,-3.9352140873<br>H,-4.565711037,0.,-0.1669647838<br>H,0.,0.,5.6892173764<br>H,5.0618506598,0.,-2.9107254496<br>H,-5.0618506598,0.,-2.9107254496<br>Cu,1.6011623667,0.,0.8671410811<br>Cu,0.,0.,-1.9164068818<br>Cu,-1.6011623667,0.,0.8671410811<br>F,6.5781771841,0.,-3.583991933<br>F,-6.5781771841,0.,-3.583991933

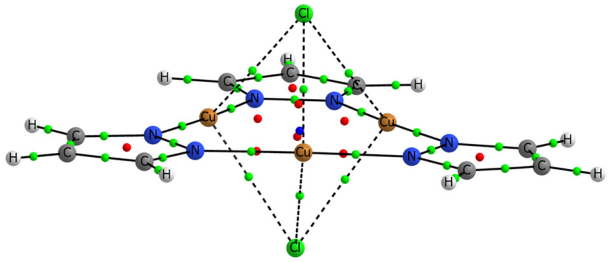	MP2= -2184.71936737 NIMAG= 0<br>X<br>X,1,r21<br>X,1,r21,2,120.<br>X,1,r21,2,120.,3,180.,0<br>N,2,r52,1,90.,3,0.,0<br>N,2,r52,1,90.,4,0.,0<br>N,3,r52,1,90.,2,0.,0<br>N,3,r52,1,90.,4,0.,0<br>N,4,r52,1,90.,3,0.,0<br>N,4,r52,1,90.,2,0.,0<br>C,5,r115,2,a1152,1,180.,0

	C,6,r115,2,a1152,1,180.,0<br>C,7,r115,3,a1152,1,180.,0<br>C,8,r115,3,a1152,1,180.,0<br>C,9,r115,4,a1152,1,180.,0<br>C,10,r115,4,a1152,1,180.,0<br>C,1,r171,3,120.,2,0.,0<br>C,1,r171,4,120.,3,0.,0<br>C,1,r171,2,120.,4,0.,0<br>H,11,r2011,5,a20115,2,180.,0<br>H,12,r2011,6,a20115,2,180.,0<br>H,13,r2011,7,a20115,3,180.,0<br>H,14,r2011,8,a20115,3,180.,0<br>H,15,r2011,9,a20115,4,180.,0<br>H,16,r2011,10,a20115,4,180.,0<br>H,1,r261,3,120.,2,0.,0<br>H,1,r261,4,120.,3,0.,0<br>H,1,r261,2,120.,4,0.,0<br>Cu,1,r291,3,60.,2,0.,0<br>Cu,1,r291,4,60.,3,0.,0<br>Cu,1,r291,2,60.,4,0.,0<br>Cl,1,rcl,2,90.,3,90.,0<br>Cl,1,rcl,2,90.,3,-90.,0<br>r21=2.54497958<br>r52=0.68522631<br>r115=1.36423941<br>r171=4.71051379<br>r2011=1.08973696<br>r261=5.80182159<br>r291=1.77597194<br>a1152=108.19693856<br>a20115=119.70143651<br>rcl=2.04332573

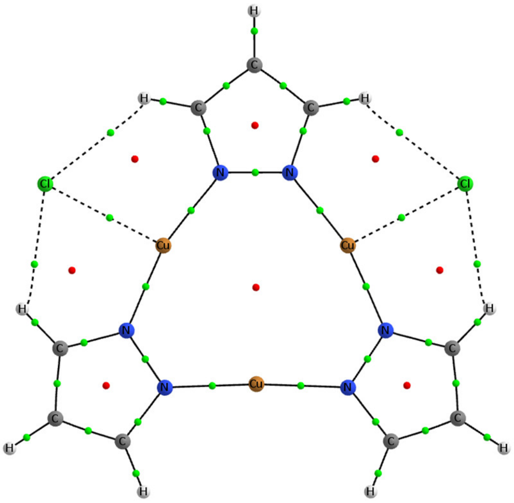	MP2= -2184.75417433 NIMAG= 0<br>N,0.7106717196,0.,2.5552085806<br>N,-0.6575304768,0.,2.476269808<br>N,2.510318538,0.,-0.65385345<br>N,1.821412967,0.,-1.8470729005<br>N,-1.8575396829,0.,-1.8930640532<br>N,-2.4732777987,0.,-0.6686968074<br>C,1.0581217499,0.,3.8750016858<br>C,-1.169216918,0.,3.7461131759<br>C,3.8495251073,0.,-0.9344670254<br>C,2.7340347366,0.,-2.8665530227<br>C,-2.8267890246,0.,-2.8538611586<br>C,-3.8288376348,0.,-0.8604850344<br>C,-0.1065249215,0.,4.6680635264<br>C,4.0340888282,0.,-2.3290822709<br>C,-4.0959240611,0.,-2.2417784751<br>H,2.1187047465,0.,4.1262126374<br>H,-2.2486923008,0.,3.8988780029<br>H,4.554198113,0.,-0.1030664731<br>H,2.3663572405,0.,-3.8925180232<br>H,-2.5140525921,0.,-3.8979584523<br>H,-4.5008735472,0.,-0.0020143437<br>H,-0.1703945117,0.,5.7563332671<br>H,4.9785630358,0.,-2.8743747089<br>H,-5.0703280978,0.,-2.7306006578<br>Cu,1.8178413009,0.,1.0623716145<br>Cu,-0.011120156,0.,-2.1054825539<br>Cu,-1.5031726613,0.,0.8678571406<br>Cl,4.053175986,0.,2.4655466819
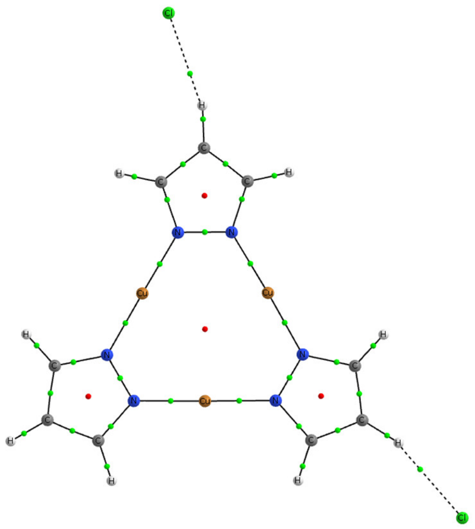	MP2= -2184.74426696 NIMAG= 0<br>N,0.6845829697,0.,2.4234402938<br>N,-0.6845829697,0.,2.4234402938<br>N,2.499640814,0.,-0.7222221747<br>N,1.8187363245,0.,-1.9148471021<br>N,-1.8187363245,0.,-1.9148471021<br>N,-2.499640814,0.,-0.7222221747<br>C,1.1128299442,0.,3.7204594977<br>C,-1.1128299442,0.,3.7204594977<br>C,3.842013863,0.,-0.9994484106<br>C,2.7471323694,0.,-2.9234615078<br>C,-2.7471323694,0.,-2.9234615078<br>C,-3.842013863,0.,-0.9994484106<br>C,0.,0.,4.5795374915

	C,4.0472053496,0.,-2.3877562736
	C,-4.0472053496,0.,-2.3877562736
	H,2.1792701299,0.,3.9411612076
	H,-2.1792701299,0.,3.9411612076
	H,4.5708850911,0.,-0.1900494603
	H,2.4136725845,0.,-3.9606730566
	H,-2.4136725845,0.,-3.9606730566
	H,-4.5708850911,0.,-0.1900494603
	H,0.,0.,5.6682888006
	H,5.0180968728,0.,-2.8985250395
	H,-5.0180968728,0.,-2.8985250395
	Cu,1.6019874969,0.,0.8494690479
	Cu,0.,0.,-1.9376702754
	Cu,-1.6019874969,0.,0.8494690479
	Cl,7.5113542459,0.,-3.3426250284
	Cl,-7.5113542459,0.,-3.3426250284

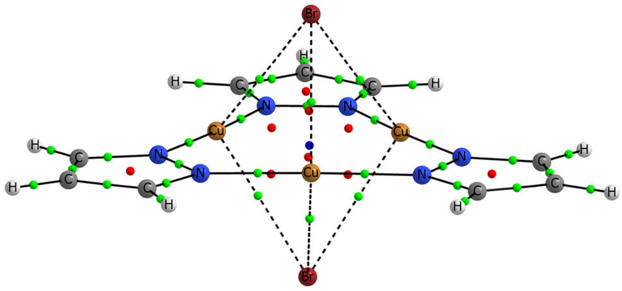	MP2= -2096.70196565 NIMAG= 0
	X
	X,1,r21
	X,1,r21,2,120.
	X,1,r21,2,120.,3,180.,0
	N,2,r52,1,90.,3,0.,0
	N,2,r52,1,90.,4,0.,0
	N,3,r52,1,90.,2,0.,0
	N,3,r52,1,90.,4,0.,0
	N,4,r52,1,90.,3,0.,0
	N,4,r52,1,90.,2,0.,0
	C,5,r115,2,a1152,1,180.,0
	C,6,r115,2,a1152,1,180.,0
	C,7,r115,3,a1152,1,180.,0
	C,8,r115,3,a1152,1,180.,0
	C,9,r115,4,a1152,1,180.,0
	C,10,r115,4,a1152,1,180.,0
	C,1,r171,3,120.,2,0.,0
	C,1,r171,4,120.,3,0.,0
	C,1,r171,2,120.,4,0.,0
	H,11,r2011,5,a20115,2,180.,0
	H,12,r2011,6,a20115,2,180.,0
	H,13,r2011,7,a20115,3,180.,0
	H,14,r2011,8,a20115,3,180.,0
	H,15,r2011,9,a20115,4,180.,0
	H,16,r2011,10,a20115,4,180.,0

	H,1,r261,3,120.,2,0.,0<br>H,1,r261,4,120.,3,0.,0<br>H,1,r261,2,120.,4,0.,0<br>Cu,1,r291,3,60.,2,0.,0<br>Cu,1,r291,4,60.,3,0.,0<br>Cu,1,r291,2,60.,4,0.,0<br>Br,1,rcl,2,90.,3,90.,0<br>Br,1,rcl,2,90.,3,-90.,0<br>r21=2.5388156<br>r52=0.6843305<br>r115=1.36410625<br>r171=4.70280233<br>r2011=1.08968088<br>r261=5.79387384<br>r291=1.77861966<br>a1152=108.25139646<br>a20115=119.75793801<br>rcl=2.28093476
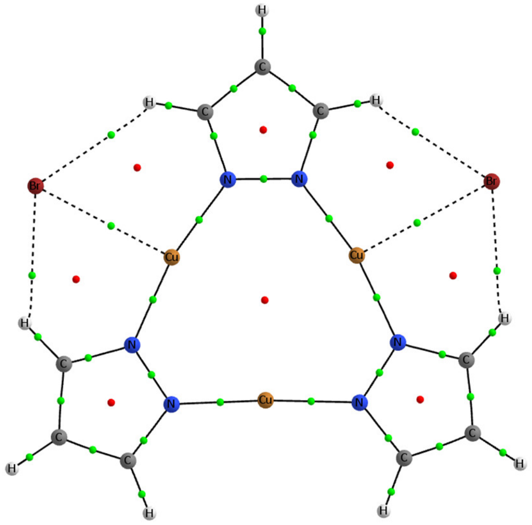	MP2= -2096.73373352 NIMAG= 0<br>N,0.7393114761,0.,2.5344823242<br>N,-0.6294307014,0.,2.4591910189<br>N,2.5348832805,0.,-0.6684485026<br>N,1.8463350247,0.,-1.8610490652<br>N,-1.8252703401,0.,-1.9075036818<br>N,-2.4444372458,0.,-0.6844925322<br>C,1.0928980976,0.,3.8525930866<br>C,-1.135305385,0.,3.7309509228<br>C,3.8739311705,0.,-0.9477480452<br>C,2.7577394689,0.,-2.8810487835<br>C,-2.7899944346,0.,-2.8727740597<br>C,-3.7987509719,0.,-0.8822721571<br>C,-0.0693571591,0.,4.6482677876<br>C,4.0566076977,0.,-2.3420835462<br>C,-4.0601965672,0.,-2.2640688322<br>H,2.1536097496,0.,4.104014218<br>H,-2.2140332558,0.,3.887961268<br>H,4.5807292503,0.,-0.1181438731<br>H,2.3926802207,0.,-3.907955962<br>H,-2.4773756954,0.,-3.9170878621<br>H,-4.4740898549,0.,-0.0265715898<br>H,-0.128454298,0.,5.7364900757<br>H,5.0006614837,0.,-2.8871332537<br>H,-5.032173283,0.,-2.7570003527

	Cu,1.8024639863,0.,1.0211923735<br>Cu,0.0168534556,0.,-2.0715757883<br>Cu,-1.4819831498,0.,0.8556233704<br>Br,4.3156784248,0.,2.5895444584<br>Br,-0.0847720727,0.,-5.0322593797
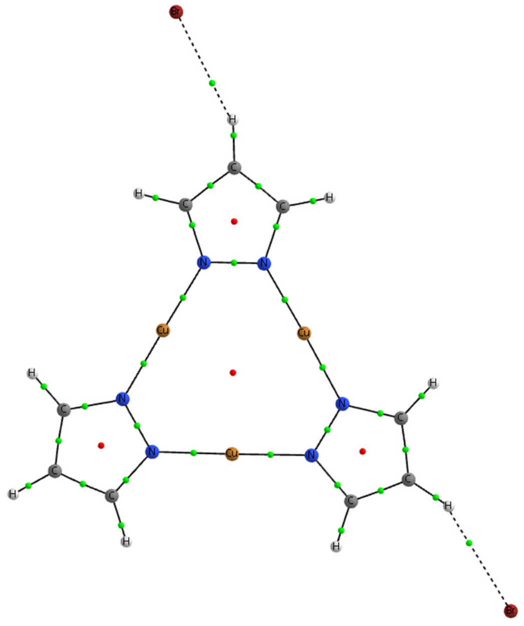	MP2= -2096.72625880 NIMAG= 0<br>N,0.684640668,0.,2.3970091193<br>N,-0.684640668,0.,2.3970091193<br>N,2.5015972041,0.,-0.7480925616<br>N,1.8183891553,0.,-1.9393115352<br>N,-1.8183891553,0.,-1.9393115352<br>N,-2.5015972041,0.,-0.7480925616<br>C,1.1128247039,0.,3.6939915846<br>C,-1.1128247039,0.,3.6939915846<br>C,3.8430691449,0.,-1.0260485163<br>C,2.7438497634,0.,-2.9501104246<br>C,-2.7438497634,0.,-2.9501104246<br>C,-3.8430691449,0.,-1.0260485163<br>C,0.,0.,4.5529452313<br>C,4.0434354688,0.,-2.4146671922<br>C,-4.0434354688,0.,-2.4146671922<br>H,2.1792525612,0.,3.9147735387<br>H,-2.1792525612,0.,3.9147735387<br>H,4.5767367049,0.,-0.221179178<br>H,2.4081246109,0.,-3.9865194215<br>H,-2.4081246109,0.,-3.9865194215<br>H,-4.5767367049,0.,-0.221179178<br>H,0.,0.,5.6416634889<br>H,5.0133071047,0.,-2.9206231268<br>H,-5.0133071047,0.,-2.9206231268<br>Cu,1.6036652184,0.,0.8242932915<br>Cu,0.,0.,-1.9597541948<br>Cu,-1.6036652184,0.,0.8242932915<br>Br,7.7686406869,0.,-2.990942837<br>Br,-7.7686406869,0.,-2.990942837

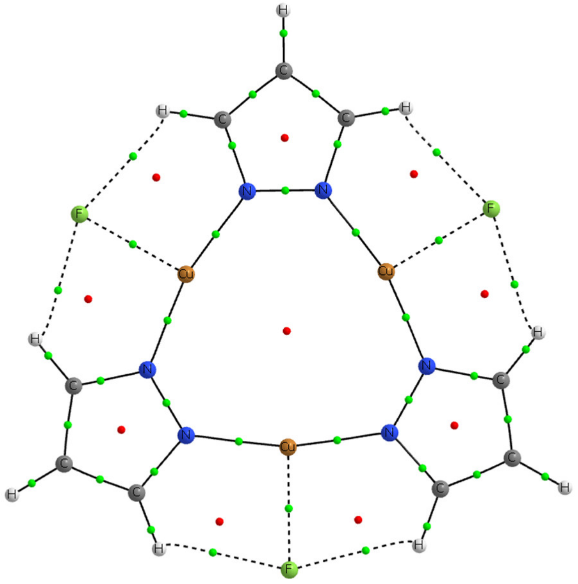	MP2= -1564.21127878 NIMAG= 0<br>N,0.6868406381,0.,2.5221144355<br>N,-0.6868220739,0.,2.5220744169<br>N,2.5276064594,0.,-0.6662517715<br>N,1.8407655217,0.,-1.855850362<br>N,-1.8407546557,0.,-1.8558404671<br>N,-2.5275795104,0.,-0.6662161327<br>C,1.11921296,0.,3.8185088708<br>C,-1.1192561722,0.,3.8184561587<br>C,3.8665084152,0.,-0.9399840166<br>C,2.7472505743,0.,-2.8785306177<br>C,-2.7472604596,0.,-2.878509292<br>C,-3.8664839711,0.,-0.9399458364<br>C,-0.0000413589,0.,4.6814803811<br>C,4.0542540979,0.,-2.3407676893<br>C,-4.0542543435,0.,-2.340727392<br>H,2.1981862998,0.,3.9860698522<br>H,-2.1982392338,0.,3.9859711076<br>H,4.5510985176,0.,-0.0893373683<br>H,2.3528331693,0.,-3.8967136965<br>H,-2.3528647563,0.,-3.8967015357<br>H,-4.551067021,0.,-0.0892948804<br>H,-0.000066779,0.,5.7745154992<br>H,5.0008418941,0.,-2.8872980468<br>H,-5.000851175,0.,-2.88724372<br>Cu,1.8103057642,0.,1.0451731775<br>Cu,0.0000136829,0.,-2.0903045361<br>Cu,-1.8102420265,0.,1.0451580954<br>F,3.7249181008,0.,2.1505530285<br>F,0.0000020429,0.,-4.3011040213<br>F,-3.7248546014,0.,2.1505495099

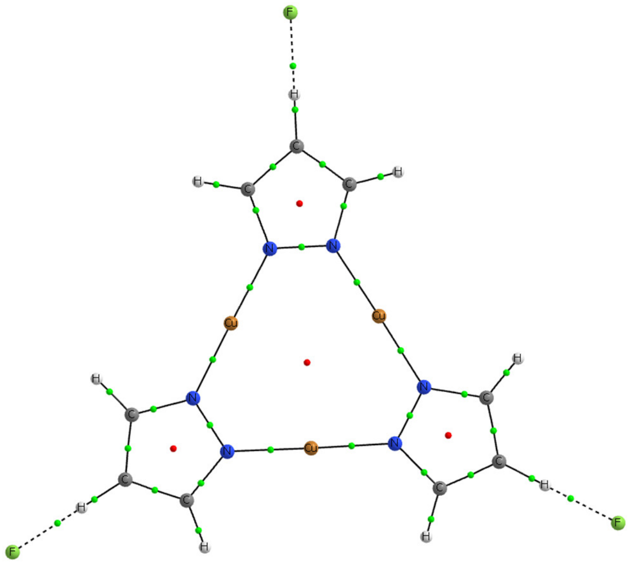

MP2= -1564.24484018 NIMAG= 0
X
X,1,r21
X,1,r21,2,120.
X,1,r21,2,120.,3,180.,0
N,2,r52,1,90.,3,0.,0
N,2,r52,1,90.,4,0.,0
N,3,r52,1,90.,2,0.,0
N,3,r52,1,90.,4,0.,0
N,4,r52,1,90.,3,0.,0
N,4,r52,1,90.,2,0.,0
C,5,r115,2,a1152,1,180.,0
C,6,r115,2,a1152,1,180.,0
C,7,r115,3,a1152,1,180.,0
C,8,r115,3,a1152,1,180.,0
C,9,r115,4,a1152,1,180.,0
C,10,r115,4,a1152,1,180.,0
C,1,r171,3,120.,2,0.,0
C,1,r171,4,120.,3,0.,0
C,1,r171,2,120.,4,0.,0
H,11,r2011,5,a20115,2,180.,0
H,12,r2011,6,a20115,2,180.,0
H,13,r2011,7,a20115,3,180.,0
H,14,r2011,8,a20115,3,180.,0
H,15,r2011,9,a20115,4,180.,0
H,16,r2011,10,a20115,4,180.,0
H,1,r261,3,120.,2,0.,0
H,1,r261,4,120.,3,0.,0
H,1,r261,2,120.,4,0.,0
Cu,1,r291,3,60.,2,0.,0
Cu,1,r291,4,60.,3,0.,0
Cu,1,r291,2,60.,4,0.,0
F,1,rf,29,60.,2,180.,0
F,1,rf,30,60.,3,180.,0
F,1,rf,31,60.,4,180.,0
r21=2.49741417
r52=0.68666322
r115=1.37336524
r171=4.6838587
r2011=1.09024204
r261=5.80563649
r291=1.85581634
a1152=107.60013957

	a20115=119.23623403
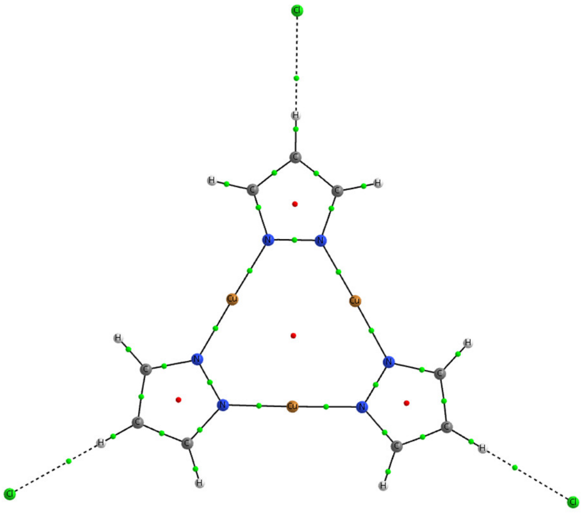	MP2= -2644.39916814 NIMAG= 0<br>N,0.6862226141,0.,2.4967553998<br>N,-0.6862226141,0.,2.4967553998<br>N,2.505331815,0.,-0.6540730086<br>N,1.8191048601,0.,-1.8426329634<br>N,-1.8191048601,0.,-1.8426329634<br>N,-2.505331815,0.,-0.6540730086<br>C,1.1071898609,0.,3.8008056819<br>C,-1.1071898609,0.,3.8008056819<br>C,3.8451448468,0.,-0.9415667973<br>C,2.7379179971,0.,-2.859266084<br>C,-2.7379179971,0.,-2.859266084<br>C,-3.8451448468,0.,-0.9415667973<br>C,0.,0.,4.6671088777<br>C,4.0417588179,0.,-2.3335856418<br>C,-4.0417588179,0.,-2.3335856418<br>H,2.1729974458,0.,4.0259735117<br>H,-2.1729974458,0.,4.0259735117<br>H,4.5730645483,0.,-0.1311485806<br>H,2.3999912066,0.,-3.894861969<br>H,-2.3999912066,0.,-3.894861969<br>H,-4.5730645483,0.,-0.1311485806<br>H,0.,0.,5.7632050926<br>H,4.9909904468,0.,-2.8816600017<br>H,-4.9909904468,0.,-2.8816600017<br>Cu,1.6071467473,0.,0.927901612<br>Cu,0.,0.,-1.8556229381<br>Cu,-1.6071467473,0.,0.927901612<br>Cl,7.3666453966,0.,-4.253152124<br>Cl,-7.3666453966,0.,-4.253152124<br>Cl,0.,0.,8.5063318427

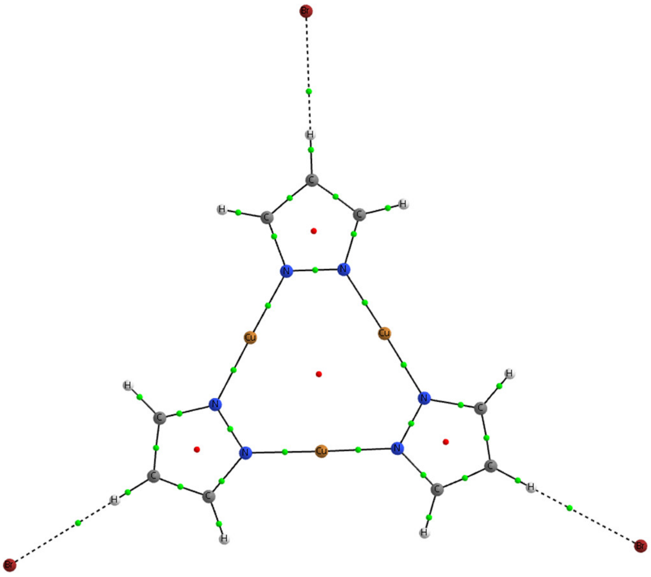	MP2= -2512.37387779 NIMAG= 0<br>N,0.686148984,0.,2.4965483731<br>N,-0.6861489841,0.,2.4965483731<br>N,2.5051641689,0.,-0.6540553825<br>N,1.8190090963,0.,-1.842495185<br>N,-1.8190090962,0.,-1.842495185<br>N,-2.5051641689,0.,-0.6540553826<br>C,1.107867224,0.,3.7998914873<br>C,-1.1078672241,0.,3.7998914873<br>C,3.8447506165,0.,-0.9405169572<br>C,2.7368696491,0.,-2.8593924462<br>C,-2.736869649,0.,-2.8593924462<br>C,-3.8447506165,0.,-0.9405169573<br>C,-0.0000000001,0.,4.6648517161<br>C,4.0398843321,0.,-2.3324402451<br>C,-4.0398843321,0.,-2.3324402452<br>H,2.1734841324,0.,4.0255790749<br>H,-2.1734841325,0.,4.0255790748<br>H,4.5730163782,0.,-0.1305148332<br>H,2.3995043024,0.,-3.8950849854<br>H,-2.3995043024,0.,-3.8950849855<br>H,-4.5730163782,0.,-0.1305148333<br>H,-0.0000000001,0.,5.7592642285<br>H,4.9876693278,0.,-2.8796528293<br>H,-4.9876693277,0.,-2.8796528294<br>Cu,1.6071310012,0.,0.9278723798<br>Cu,0.,0.,-1.8557285226<br>Cu,-1.6071310012,0.,0.9278723798<br>Br,7.5597621472,0.,-4.3645300885<br>Br,-7.5597621471,0.,-4.3645300886<br>Br,-0.0000000001,0.,8.7291967801

Table S4. Electron density properties (au) at the intermolecular BCPs in the 1:1 complexes.

1:1 Cu complexes

Configuration	Atoms	Dist	Rho	Lap	H
Apical	Cu25-F28	2.1354	0.0522	0.2611	-0.0071
Planar	F28-Cu26	2.0158	0.0683	0.3678	-0.0121
Planar	H19-F28	2.2785	0.0148	0.0541	0.0004
CH(4)	H22-F28	1.5510	0.0608	0.1809	-0.0107
Apical	Cl28-Cu25	2.5561	0.0392	0.1336	-0.0034
Planar	Cl28-Cu26	2.4297	0.0507	0.1711	-0.0084
Planar	H19-Cl28	2.5748	0.0141	0.0429	0.0009
CH(4)	H22-Cl28	2.3572	0.0192	0.0495	0.0005
Apical	Br28-Cu25	2.6924	0.0362	0.1012	-0.0040
Planar	Br28-Cu26	2.5745	0.0455	0.1243	-0.0080
Planar	H19-Br28	2.6652	0.0140	0.0404	0.0008
CH(4)	Br28-H22	2.5470	0.0160	0.0379	0.0003

1: 1 Ag complexes

Configuration	Atoms	Dist	Rho	Lap	H
Apical	F28-Ag25	2.379	0.0422	0.1823	-0.0045
Planar	F28-Ag26	2.256	0.0545	0.2623	-0.0048
Planar	H19-F28	2.483	0.0094	0.0355	0.0009
CH(4)	H22-F28	1.554	0.0605	0.1800	-0.0105
Apical	Cl28-Ag25	2.758	0.0341	0.1118	-0.0026
Planar	Cl28-Ag26	2.623	0.0443	0.1474	-0.0051
Planar	H19-Cl28	2.790	0.0094	0.0272	0.0007
CH(4)	H22-Cl28	2.366	0.0188	0.0486	0.0005
Apical	Br28-Ag25	2.856	0.0337	0.0935	-0.0039
Planar	Br28-Ag26	2.722	0.0434	0.1177	-0.0072
Planar	H19-Br28	2.889	0.0093	0.0263	0.0008
CH(4)	H22-Br28	2.556	0.0157	0.0373	0.0003

1: 1 Au complexes

Configuration	Atoms	Dist	Rho	Lap	H
Apical	Au25-F28	2.558	0.0358	0.1296	-0.0036
Planar	F28-Au26	2.434	0.0454	0.1776	-0.0046
Planar	H19-F28	2.336	0.0126	0.0433	0.0004
CH(4)	H22-F28	1.508	0.0676	0.1866	-0.0155
Apical	Cl28-Au25	3.017	0.0253	0.0738	-0.0005
Planar	Cl28-Au26	2.956	0.0283	0.0817	-0.0008

Planar	H19-Cl28	2.614	0.0127	0.0351	0.0006
CH(4)	H22-Cl28	2.317	0.0208	0.0537	0.0005
Apical	Br28-Au25	3.127	0.0246	0.0637	-0.0010
Planar	Br28-Au26	3.088	0.0265	0.0666	-0.0014
Planar	H19-Br28	2.706	0.0127	0.0336	0.0006
CH(4)	H22-Br28	2.507	0.0173	0.0406	0.0003

Table S5. Barriers ($kJ\ mol^{-1}$) and interhalogen distance (Å) in the maximum of the dissociation curve (Figure S1) of the 1:2 apical complexes.

	**Barrier**			X···X distance		
	**F^-**	**Cl^-**	**Br^-**	F^-	Cl^-	Br^-
Au	33.0	7.5	8.7	4.9	6.2	6.7
Ag	88.6	56.4	59.0	5.4	6.3	6.8
Cu	95.6	29.6	23.7	4.7	5.7	6.2

Table S6. Interatomic X···X distances (Å) in the 1:2 complexes.

F^-	1:2 apical	1:2 planar	1:2 CH(4)
Au	3.534	12.219*	13.360
Ag	3.165	8.343	13.602
Cu	2.697	7.370	13.156
**Cl^-**			
Au	5.206	9.323	15.182
Ag	4.330	8.886	15.484
Cu	4.087	8.324	15.023
**Br^-**			
Au	5.483	9.510	15.699
Ag	4.627	9.097	16.019
Cu	4.562	8.801	15.537

* In this complex, the F(-) anions are interacting with the C(3)-H.

Table S7. Anion-Anion repulsion corrected binding energies (kJ/mol) for the 1:2 complexes.

F^-	1:2 apical	1:2 planar	1:2 CH(4)
Au	-280.1	-149.4	-143.6
Ag	-485.4	-245.5	-120.7
Cu	-549.9	-270.8	-125.9
**Cl^-**			
Au	-127.4	-125.1	-75.7
Ag	-297.6	-182.2	-60.5
Cu	-246.5	-164.8	-64.4
**Br^-**			
Au	-133.0	-125.9	-69.7
Ag	-285.0	-176.7	-55.5
Cu	-210.5	-147.3	-59.1

Table S8. Electron density properties (au) at the intermolecular BCPs in the 1:2 complexes.

1:2 Cu complexes

Configuration	Atoms	Dist	Rho	Lap	H
Apical	F28-F29	2.6975	0.0197	0.0744	-0.0010
Apical	Cu25-F28	2.2109	0.0442	0.2059	-0.0055
Planar	F28-Cu25	2.0895	0.0577	0.2947	-0.0087
Planar	F28-H18	2.4934	0.0095	0.0383	0.0011
Planar	H16-F28	2.2599	0.0153	0.0538	0.0002
CH(4)	F28-H23	1.6591	0.0471	0.1519	-0.0043
Apical	Cl28-Cu25	2.7073	0.0293	0.0928	-0.0012
Planar	Cl28-Cu25	2.6392	0.0333	0.1072	-0.0020
Planar	Cl28-H18	2.6170	0.0127	0.0376	0.0008
Planar	H16-Cl28	2.5495	0.0146	0.0430	0.0008
CH(4)	Cl28-H23	2.5325	0.0138	0.0352	0.0004
Apical	Br28-Cu25	2.8924	0.0252	0.0650	-0.0014
Planar	Cu25-Br28	2.9624	0.0222	0.0539	-0.0011
Planar	H18-Br28	2.7206	0.0123	0.0340	0.0007
Planar	H16-Br28	2.6397	0.0144	0.0392	0.0006
CH(4)	Br28-H23	2.7562	0.0110	0.0275	0.0005

1:2 Ag complexes

Configuration	Atoms	Dist	Rho	Lap	H
Apical	Ag26-F28	2.437	0.0372	0.1526	-0.0042
Planar	F28-Ag25	2.327	0.0467	0.2100	-0.0046
Planar	H16-F28	2.179	0.0173	0.0555	-0.0004
CH(4)	F28-H23	1.660	0.0470	0.1514	-0.0043
Apical	Cl28-Ag25	2.845	0.0287	0.0923	-0.0015
Planar	Cl28-Ag25	2.734	0.0354	0.1156	-0.0028
Planar	H16-Cl28	2.720	0.0106	0.0301	0.0006
Planar	Cl28-H18	2.971	0.0068	0.0200	0.0007
CH(4)	Cl28-H23	2.538	0.0137	0.0348	0.0004
Apical	Br28-Ag25	2.949	0.0282	0.0785	-0.0023
Planar	Br28-Ag25	2.849	0.0340	0.0932	-0.0039
Planar	Br28-H18	3.001	0.0075	0.0216	0.0008
Planar	H16-Br28	2.827	0.0103	0.0286	0.0007
CH(4)	Br28-H23	2.775	0.0107	0.0269	0.0005

1:2 Au complexes

Configuration	Atoms	Dist	Rho	Lap	H
Apical	Au25-F28	2.613	0.0320	0.1118	-0.0032

Planar	F28-H16	1.592	0.0552	0.1725	-0.0074
CH(4)	F28-H23	1.619	0.0517	0.1635	-0.0061
Apical	Cl28-Au25	3.226	0.0174	0.0482	0.0002
Planar	Au26-Cl29	3.325	0.0148	0.0391	0.0003
Planar	H16-Cl28	2.591	0.0130	0.0340	0.0005
Planar	Cl28-H18	2.977	0.0065	0.0179	0.0005
CH(4)	Cl28-H23	2.478	0.0153	0.0391	0.0005
Apical	Br28-Au25	3.339	0.0169	0.0436	0.0001
Planar	Br29-Au26	3.455	0.0140	0.0349	0.0003
Planar	H16-Br28	2.727	0.0120	0.0304	0.0005
Planar	H18-Br28	2.996	0.0074	0.0198	0.0006
CH(4)	Br28-H23	2.696	0.0123	0.0304	0.0005

Table S9. Interatomic distances (Å) of the anions in the 1:3 complexes.

**F(-)**	**Planar**	**CH(4)**
Au		13.361
Ag	8.158	13.573
Cu	7.450	13.141
**Cl(-)**	planar	CH(4)
Au		14.866
Ag	9.038	15.169
Cu		14.733
**Br(-)**	planar	CH(4)
Au		15.226
Ag	9.353	15.559
Cu		15.120

Table S10. Anion-Anion repulsion corrected binding energies (kJ/mol) in the 1:3 complexes.

**F(-)**	**Planar**	**CH(4)**
Au		-175.8
Ag	-286.3	-146.8
Cu	-307.4	-153.3
**Cl(-)**	planar	CH(4)
Au		-92.3
Ag	-201.0	-71.7
Cu		-76.6
**Br(-)**	planar	CH(4)
Au		-85.6
Ag	-193.4	-66.0
Cu		-70.7

Table S11. Electron density properties at the intermolecular BCPs in the 1:3 complexes.

1:3 Cu complexes

Configuration	Atoms	Dist	Rho	Lap	H
Planar	F28-Cu25	2.2108	0.0443	0.2049	-0.0053
Planar	F28-H16	2.3875	0.0116	0.0425	0.0007
CH(4)	F30-H22	1.7813	0.0357	0.1149	-0.0018
CH(4)	H22-Cl30	2.7431	0.0093	0.0231	0.0003
CH(4)	Br30-H22	2.9699	0.0074	0.0182	0.0005

1:3 Ag complexes

Configuration	Atoms	Dist	Rho	Lap	H
Planar	Ag27-F30	2.411	0.0392	0.1624	-0.0042
Planar	F28-H16	2.461	0.0098	0.0350	0.0007
CH(4)	F30-H22	1.777	0.0360	0.1160	-0.0019
Planar	Cl30-Ag27	3.025	0.0204	0.0615	-0.0002
Planar	H16-Cl28	2.863	0.0081	0.0225	0.0006
CH(4)	Cl28-H23	2.740	0.0094	0.0232	0.0003
Planar	Br30-Ag27	3.238	0.0166	0.0448	0.0001
Planar	H16-Br28	2.942	0.0082	0.0223	0.0007
CH(4)	H24-Br29	2.969	0.0074	0.0182	0.0005

1:3 Au complexes

Configuration	Atoms	Dist	Rho	Lap	H
CH(4)	F30-H22	1.735	0.0395	0.1280	-0.0025
CH(4)	H22-Cl30	2.651	0.0110	0.0273	0.0003
CH(4)	H22-Br30	2.864	0.0089	0.0217	0.0005

Table S12. Anion-Metal distances in the CSD search.

Cu-F

Refcode	Distances (Å)		
HUXWUU	2.383	2.579	2.482
HUXWUU	2.605	2.536	2.448
	Average	Maximum	Minimum
	2.506	2.605	2.383

Cu-Cl

Refcode	Distances (Å)		
JALKIT	2.622	2.424	2.486
OBOQAY	2.359	2.545	2.923
OBOQEC	2.361	2.549	2.931
OBOQIG	2.354	2.560	2.977
RETQIR	2.500	2.584	2.558
RETQIR	2.628	2.501	2.614
RUYGUN	2.461	2.453	3.056
RUYGUN	2.641	2.965	2.350
RUYGUN	2.486	2.398	3.039
RUYGUN	2.756	2.756	2.341
RUYHAU	2.600	2.986	2.357
UWOMAW	2.626	2.502	2.630
UWOMAW	2.648	2.761	2.581
VADYAB	2.623	2.603	2.603
VAZCUX	2.527	2.631	2.666
VAZCUX	2.604	2.527	2.564
	Average	Maximum	Minimum
	2.609	3.056	2.341

Cu-Br

Refcode	Distances (Å)		
ELODIS	2.722	3.063	2.504
ELODOY	2.652	2.750	2.645
ELODOY	2.698	2.638	2.806
	Average	Maximum	Minimum
	2.720	3.063	2.504

Figure S1. Energy profiles (kJ/mol) as a function of the X-X distance (Å) in the 1:2 apical complexes. Only one of the X anions in move further away from the $(Pz\text{-}M)_3{:}X^-$ system.

X= F(-) Cl(-) Br(-)

M= Au

Ag

Cu

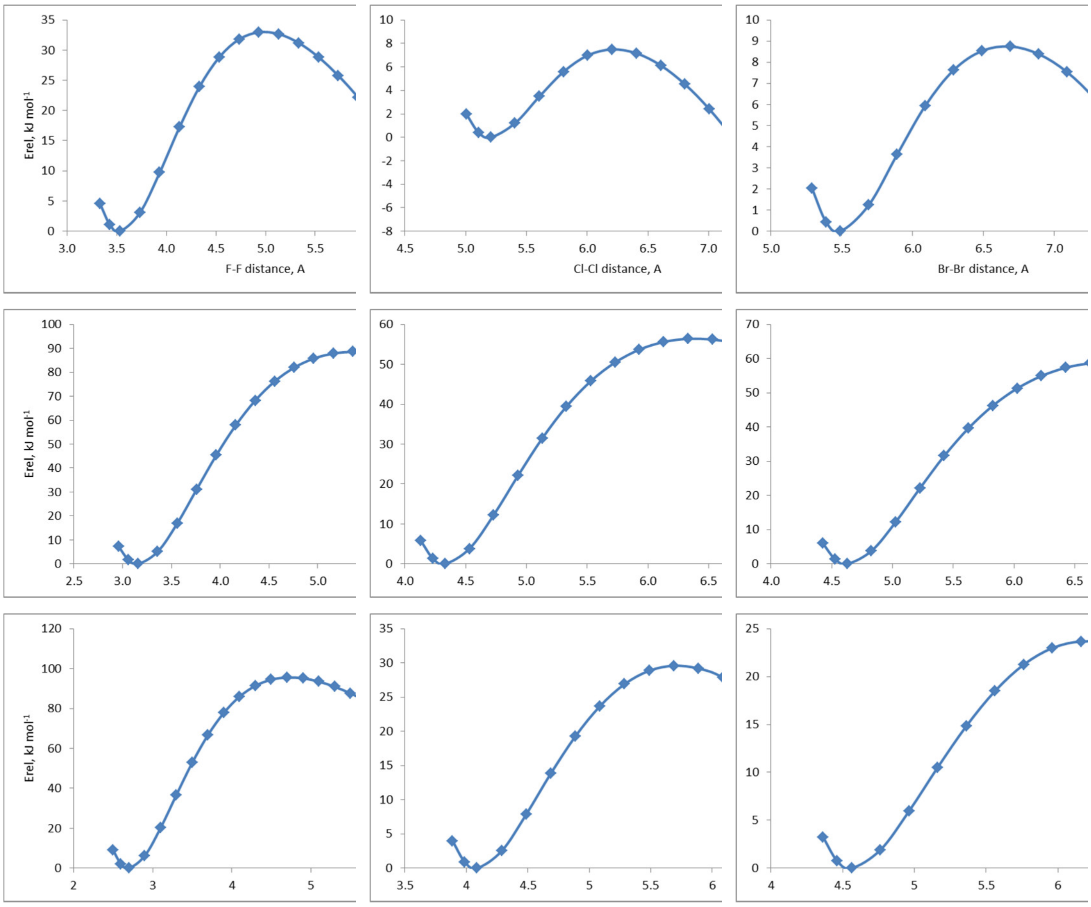

Figure S2. Electron density at the BCP (au) vs. the interatomic distance (Å).

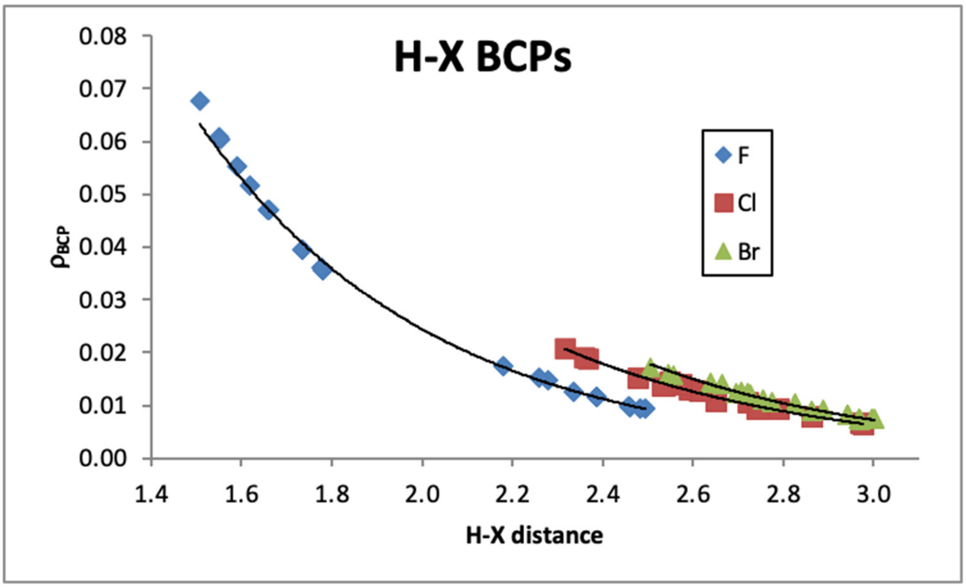

The exponential relationships show R^2 values of 0.998 (H-F), 0.98 (H-Cl) and 0.98 (H-Br).

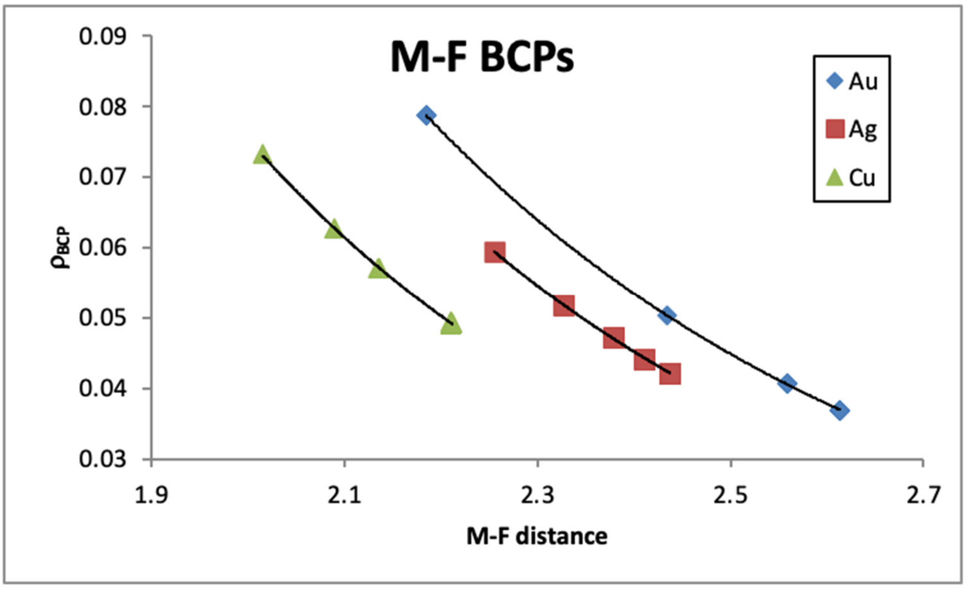

The exponential relationships show R^2 values > 0.999.

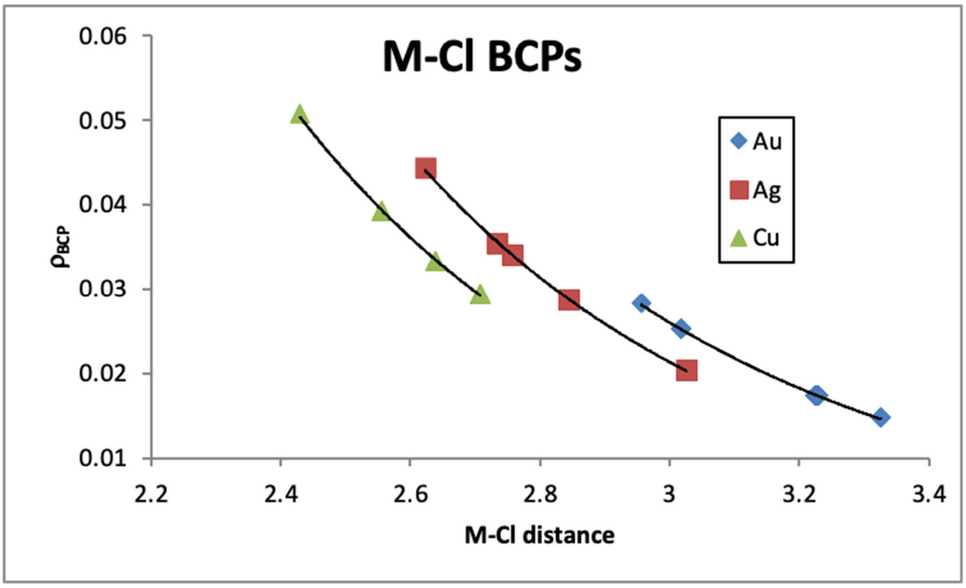

The exponential relationships show R^2 values > 0.999.

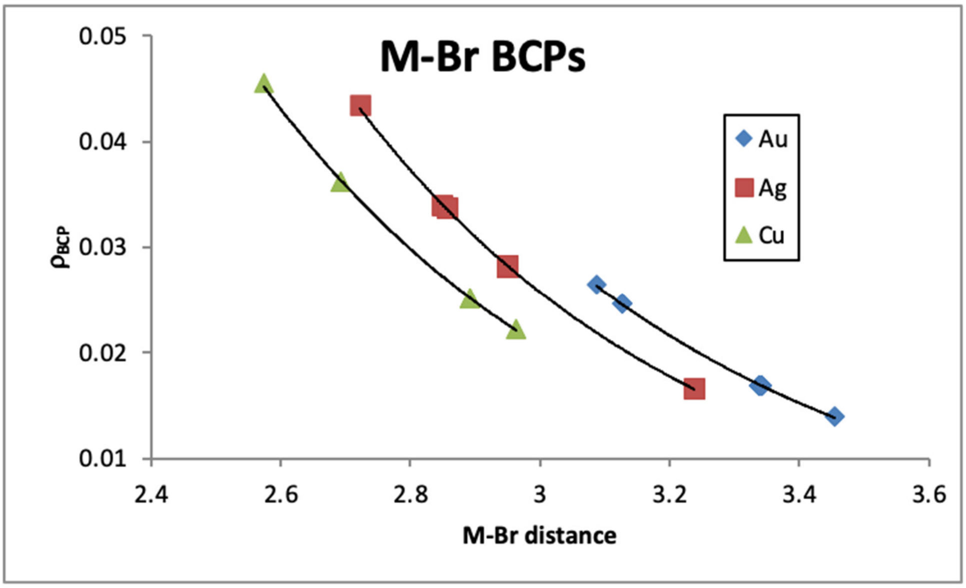

The exponential relationships show R^2 values > 0.99.
